# Elucidation of the Antimycobacterial Activity of D-Form Human Lactoferricin 1–11 (D-Form hLF 1–11) Against *Mycobacterium smegmatis* Through Proteomics and Imaging Analysis

**DOI:** 10.3390/antibiotics15060607

**Published:** 2026-06-15

**Authors:** Sorasak Intorasoot, Whichayanan Doung-Arpai, Amornrat Intorasoot, Khajornsak Tragoolpua, Sirikwan Sangboonruang, Bordin Butr-Indr, Usanee Wattananandkul, Ponrut Phunpae, Chayada Sitthidet Tharinjaroen

**Affiliations:** 1 Division of Clinical Microbiology, Department of Medical Technology, Faculty of Associated Medical Sciences, Chiang Mai University, Chiang Mai 50200, Thailand; 691135903@cmu.ac.th (W.D.-A.); khajornsak.tr@cmu.ac.th (K.T.); sirikwan.sang@cmu.ac.th (S.S.); bordin.b@cmu.ac.th (B.B.-I.); usanee.anukool@cmu.ac.th (U.W.); ponrut.p@cmu.ac.th (P.P.); chayada.si@cmu.ac.th (C.S.T.); 2 Infectious Diseases Research Unit, Faculty of Associated Medical Sciences, Chiang Mai University, Chiang Mai 50200, Thailand; 3 Department of Microbiology, Faculty of Medicine, Chiang Mai University, Chiang Mai 50200, Thailand; amornrat.in@cmu.ac.th

**Keywords:** mycobacteria, Mycobacterium smegmatis, human lactoferricin 1–11, proteomics

## Abstract

**Background/Objectives**: Tuberculosis (TB), caused by *Mycobacterium tuberculosis* complex, remains a major global health challenge. Recently, D-enantiomer of human lactoferricin 1–11 (D-form hLF 1–11), a short peptide derived from the N-terminal region of lactoferrin, has demonstrated potent antimycobacterial activity. However, its direct mechanism of action has not yet been elucidated. **Methods & Results**: In the present study, *M. smegmatis* was employed as a model organism to investigate the mechanism underlying D-form hLF 1–11 activity. Initially, the minimum inhibitory concentration (MIC) was determined and the results revealed growth inhibition at 400 µg/mL. Live/dead fluorescence staining demonstrated mycobactericidal activity, as indicated by increased propidium iodide (PI) uptake relative to the untreated control. Scanning electron microscopy and high-resolution fluorescence microscopy revealed membrane disruption and substantial morphological deformation, along with a time-dependent accumulation of the peptide at the membrane and inside the cells. Furthermore, label-free quantitative proteomic analysis of peptide-treated cells revealed extensive metabolic alterations in carbon metabolism, acetyl-CoA-dependent lipid biosynthesis, oxidative stress defense, translational machinery, and energy production systems. **Conclusions**: Collectively, these findings provide mechanistic insights into the antimycobacterial activity of D-form hLF 1–11 against *M. smegmatis*.

## 1. Introduction

Tuberculosis (TB), caused by the *Mycobacterium tuberculosis* complex (MTC), remains one of the leading infectious causes of mortality worldwide [1,2,3]. The pathogen primarily affects the lungs but can also impact other parts of the body. According to the World Health Organization (WHO), an estimated 10.7 million developed TB and approximately 1.23 million TB-related deaths were reported globally in 2024 [4]. Combination drug therapy is the standard regimen for TB treatment. Isoniazid and rifampicin are the first-line anti-TB drugs and are highly effective against drug-susceptible *M. tuberculosis*. Nevertheless, their clinical effectiveness is hindered by prolonged treatment, poor penetration into granulomatous lesions and the occurrence of several adverse effects. Moreover, inappropriate or incomplete anti-TB treatment has accelerated the emergence and dissemination of multidrug-resistant (MDR) and extensively drug-resistant (XDR) TB [5]. These challenges contribute to treatment failure, disease relapse, and continued transmission. Therefore, the development of novel therapeutic agents is urgently needed.

Antimicrobial peptides (AMPs) have emerged as a promising class of therapeutic agents for combating infectious diseases. Although AMPs exhibit potent antimicrobial activity, their clinical application is limited by a short half-life resulting from rapid degradation by endogenous proteolytic enzymes. Substitution with D-enantiomer peptide significantly improves stability in biological environments by enhancing resistance to protease-mediated cleavage. The increased stability of the D-form peptide prolongs systemic persistence, reduces dosing frequency, and enhances its suitability for therapeutic applications [6,7]. Over the past decade, several AMPs with potent antimycobacterial properties have been identified, such as LL-37 [8] and human β-defensin [9]. Human lactoferricin 1–11 (hLF 1–11), an 11-amino-acid peptide derived from the N-terminal region of the iron-binding glycoprotein lactoferrin, has been extensively studied for its broad-spectrum antimicrobial activity [10,11].

Recently, the antimycobacterial activity of D-form hLF 1–11 against susceptible and MDR *M. tuberculosis*, as well as the non-tuberculous mycobacteria, *M. avium* and *M. intracellulare*, has been explored in our laboratory [12]. The immunomodulatory activity of D-form hLF 1–11-loaded niosomes against *M. tuberculosis* residing within alveolar macrophages was investigated through proteomic analysis [13]. Consequently, the direct mechanism of action of D-form hLF 1–11 against mycobacterium remains unexplored.

Due to its significant genetic and phenotypic similarities with *M. tuberculosis*, the fast-growing and non-pathogenic *M. smegmatis* was used as a model organism in this study [14]. The MIC of D-form hLF 1–11 against *M. smegmatis* was determined using the resazurin microplate assay (REMA). Live/dead fluorescent staining and the time–kill kinetics assay were subsequently evaluated to assess its bactericidal activity. Changes in the structural morphology of *M. smegmatis* following peptide treatment were examined by scanning electron microscopy (SEM), while peptide localization within the bacterial cells was visualized using high-resolution fluorescent microscopy. Finally, the cellular response of *M. smegmatis* to D-form hLF 1–11 was investigated through label-free quantitative proteomic analysis.

## 2. Results

### 2.1. The Antimycobacterial Activity of D-Form hLF 1–11 Against M. smegmatis

**Figure 1:**
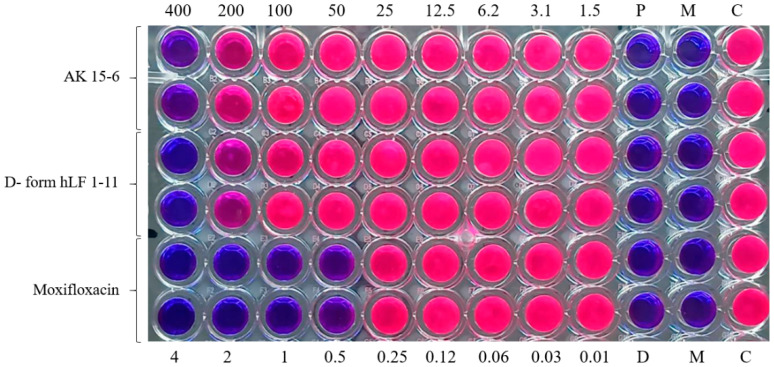
Antimycobacterial activity determination of D-form hLF 1–11 against *M. smegmatis* using REMA.

The antimycobacterial activity of D-form hLF 1–11 against *M. smegmatis* ATCC 14468 was assessed in accordance with CLSI guidelines, and the MIC was determined using the REMA. The peptide effectively inhibited the growth of *M. smegmatis*, with an MIC of 400 µg/mL. This MIC value was comparable to that of AK 15-6, a previously reported anti-tubercular peptide. Moxifloxacin was included as a positive control, and its MIC was within the acceptable CLSI range (≤1 µg/mL). The results are presented in Figure 1.

The antimycobacterial activity of D-form hLF 1–11 was determined using the REMA. Peptide was two-fold serially diluted from 1.5 to 400 µg/mL. Peptide control and media control were included as negative controls, whereas untreated cells served as the growth control. In addition, the anti-tubercular peptide AK15-6 and the antibiotic moxifloxacin were used as positive controls in this study. All experiments were performed in duplicate and repeated independently three times.

### 2.2. Co-Fluorescent Staining for Live/Dead Determination of Bacterial Cells

**Figure 2:**
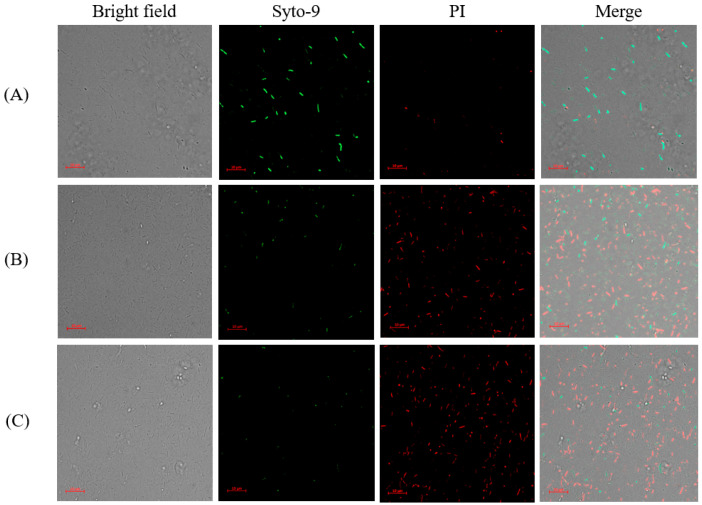

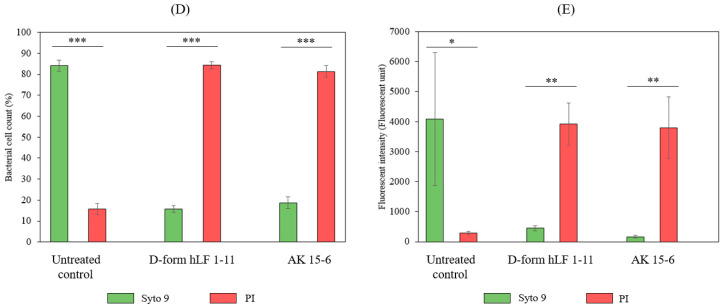
Live–dead fluorescent staining for bactericidal activity determination of D-form hLF 1–11 against *M. smegmatis.* Bactericidal activity of D-form hLF 1–11 against *M. smegmatis* was evaluated using SYTO 9 and PI co-fluorescent staining. Significant differences in bacterial viability (green) and cell death (red) between the untreated control (**A**) and D-form hLF 1–11-treated group (**B**) were analyzed based on the percentage of cell counts (**D**) and fluorescence intensity (**E**). *M. smegmatis* treated with AK 15-6 peptide was used as a positive control (**C**). Statistical significance was defined as follows: * *p* < 0.05, ** *p* < 0.01, and *** *p* < 0.0001. Scale bars represent 10 µm.

The bactericidal activity of D-form hLF 1–11 against *M. smegmatis* was evaluated using SYTO 9/PI dual-fluorescence staining. After incubation, the PI fluorescence intensity in the D-form hLF 1–11-treated group was significantly higher, both in terms of the number of cells and fluorescent intensity, compared to the untreated control. AK15-6 was included as a positive control. The fluorescence images of the untreated control and *M. smegmatis* treated with D-form hLF 1–11 or AK-15-6 are shown in Figure 2A–C, respectively. Quantitative data for cell counts and fluorescence intensity are presented in Figure 2D,E.

### 2.3. The Time–Kill Assay of D-Form hLF 1–11 Against M. smegmatis

**Figure 3:**
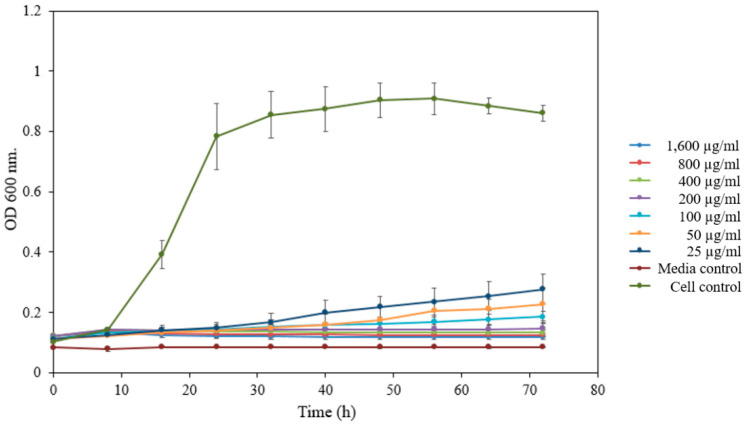
The time–kill kinetic of D-form hLF 1–11 against *M. smegmatis*.

The effect of D-form hLF 1–11 on *M. smegmatis* growth was evaluated by monitoring OD_600_ over time. A dose-dependent growth profile was observed at D-form hLF 1–11 concentrations ranging from 0 to 1600 µg/mL. As shown in Figure 3, concentrations of D-form hLF 1–11 ≥ 400 µg/mL completely inhibited *M. smegmatis* growth. For subsequent proteomic analysis, the optimal inhibitory condition was determined to be 400 µg/mL for 20 h.

Approximately 10^6^ CFU/mL of *M. smegmatis* was incubated with D-form hLF 1–11 at final concentrations ranging from 25 to 1600 µg/mL, at 37 °C for 72 h. Bacterial growth was monitored kinetically at 8 h intervals by measuring OD_600_. Media and untreated cells were used as negative and growth controls, respectively. Growth curves were plotted as mean ± SD of OD_600_ versus incubation time. Experiments were performed in duplicate and repeated independently three times.

### 2.4. SEM Analysis of Morphological Alterations in D-Form hLF 1–11-Treated M. smegmatis

**Figure 4:**
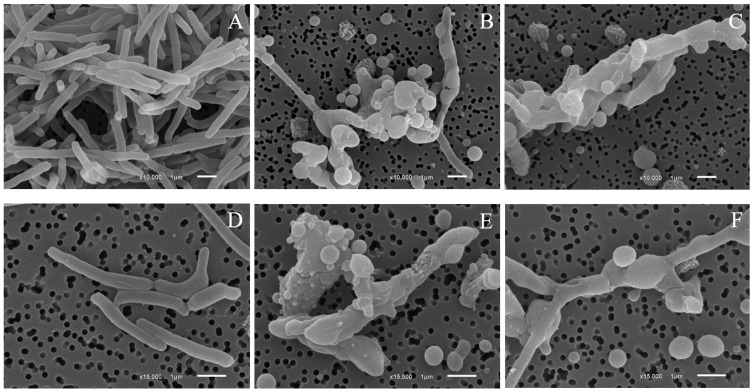
Scanning electron microscopy (SEM) images of surface morphological alterations of *M. smegmatis* following D-form hLF 1–11 treatment. SEM was used to examine surface morphological alterations in *M. smegmatis* following treatment with D-form hLF 1–11. Approximately 10^8^ CFU/mL *M. smegmatis* was incubated with 400 µg/mL (1 × MIC) or 800 µg/mL (2 × MIC) D-form peptide. Untreated cell control exhibited well-defined morphology, appearing as straight, slender rods with smooth and intact surfaces (**A**,**D**). In contrast, cells treated with 400 µg/mL (**B**,**E**) and 800 µg/mL (**C**,**F**) D-form hLF 1–11 displayed pronounced morphological alterations, including warped cell shapes, apparent cell fusion, and the presence of small spherical structures. Scale bars indicate 1 µm.

SEM was used to evaluate surface morphological alterations in *M. smegmatis* following exposure to D-form hLF 1–11. Isopore filters served as the attachment surface during bacterial growth. The morphology of *M. smegmatis* treated with 400 µg/mL (1 × MIC) or 800 µg/mL (2 × MIC) D-form hLF 1–11 was examined, with untreated cells serving as the control. SEM images are presented in Figure 4. Control cells exhibited well-defined morphology, appearing as straight, slender rods with smooth and intact surfaces (Figure 4A,D). In contrast, D-form hLF 1–11-treated cells displayed pronounced morphological alterations, including warped cell shapes, apparent cell fusion, and the presence of small spherical structures, as illustrated in Figure 4B,E (400 µg/mL) and Figure 4C,F (800 µg/mL).

### 2.5. Fluorescent Microscopy for Peptide Localization

**Figure 5:**
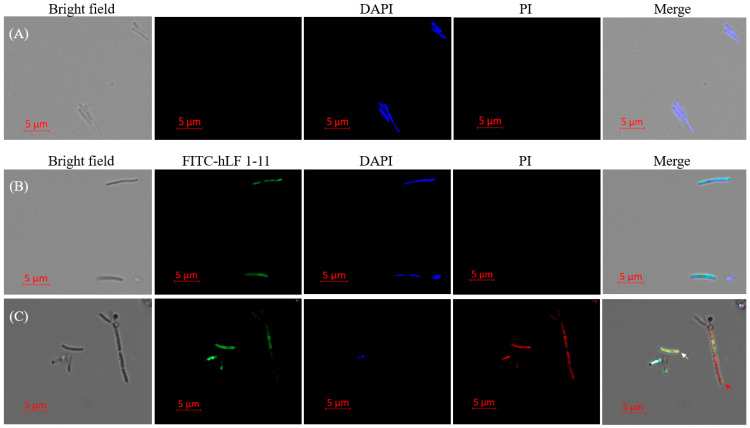
Localization of D-form hLF 1–11 peptide in *M. smegmatis.* Localization of D-form hLF 1–11 was assessed by tricolor fluorescent staining using FITC-D-form hLF 1–11 (green), DAPI (blue) and PI (red). Approximately 10^8^ CFU/mL of *M. smegmatis* was incubated with peptide-solubilizing buffer (**A**) or 1600 µg/mL (4 × MIC) FITC-D-form hLF 1–11 for 10 min (**B**) or 1 h (**C**), following by imaging with a high-resolution fluorescence microscope using Z-stacks for colocalization analysis. Red arrow indicates peptide accumulation on bacterial surface membrane, whereas white arrow indicates intracellular peptide localization. Scale bars represent 5 µm.

The interaction between the D-form hLF 1–11 antimicrobial peptide and the bacterial membrane was preliminarily examined using tricolor fluorescence labeling combined with high-resolution fluorescence microscopy. FITC-labeled D-form hLF 1–11 (green) was used to visualize the spatial distribution of the peptide in *M. smegmatis*. Bacterial nucleic acids were stained with DAPI (blue), while membrane damage was assessed using the membrane-impermeant intercalating dye PI (red), which penetrates cells with compromised membranes. Untreated cells were included as a negative control for fluorescence staining (Figure 5A). At early time points, FITC-labeled D-form hLF 1–11 was mainly localized on the bacterial surface (Figure 5B). With prolonged incubation, increased peptide accumulation on the membrane and within the cells was observed, together with enhanced PI uptake and nucleic acid staining (Figure 5C). These results suggest that D-form hLF 1–11 disrupts the membrane integrity of *M. smegmatis* in a time-dependent manner.

### 2.6. Proteomic Profiling of M. Smegmatis in Response to D-Form hLF 1–11

**Figure 6:**
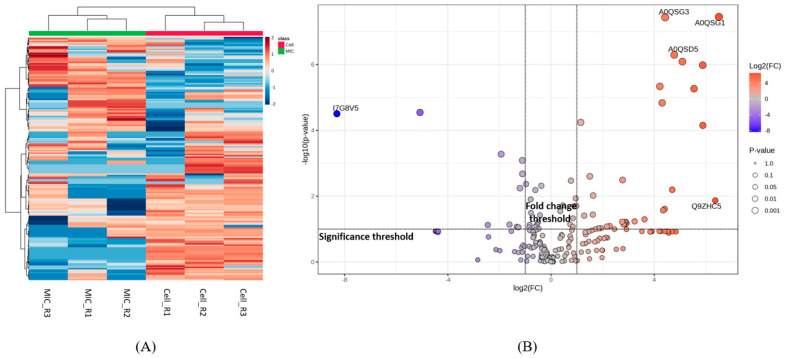
Heatmap and volcano plot of proteins from untreated cell control compared to D-form hLF 1–11-treated cells. The heatmap represents the abundance of proteins in *M. smegmatis* in response to D-form hLF 1–11 treatment compared with the untreated cell control (**A**). Protein abundance was indicated by a color gradient ranging from high (red) to low (blue). Cell R1–R3 and MIC R1–R3 represent untreated control cells and D-form hLF 1–11-treated cells from three independent experiments, respectively. The volcano plot illustrates protein fold changes between untreated control and D-form hLF 1–11-treated cells (Cell/MIC) (**B**). The *x*-axis represents the log2 fold change whereas the *y*-axis represents the -log10 *p*-value. Thirty-eight proteins that exceeded the significance and fold-change thresholds are highlighted.

*Mycobacterium smegmatis* cell pellets were extracted with ABC buffer and a homogenizer. Overall, protein concentrations obtained from D-form hLF 1–11-treated groups were lower than the untreated control, with means of protein concentrations of 0.206 and 0.510 mg/mL, respectively. Hence, the protein concentrations were adjusted to an equivalent optical density (OD) prior to LC-MS analysis. Based on heatmap analysis, protein profiles between the D-form hLF 1–11-treated groups and cell control groups were highly different (Figure 6A). Among the 223 protein groups detected (Table S1), a volcano plot indicated 38 significantly different protein groups between the two groups (*p*-value < 0.05) (Figure 6B). In addition, a principal component analysis (PCA) score plot, partial least squares discriminant analysis (PLS-DA) and fifteen proteins with variable importance in projection (VIP) scores analyzed by orthogonal partial least squares discriminant analysis (OPLSDA) are illustrated in Figure S1.

### 2.7. Protein–Protein–Chemical Interaction (PPCI) Network Analysis of Bacterial Proteins in Response to D-Form hLF 1–11

Most significantly altered proteins were more abundant in the untreated control group than in the D-form hLF 1–11-treated groups. These included ribosomal proteins rpsG (P41193), rpsQ (A0QSE0), rpsH (A0QSG3), rpsR2 (A0R7F7), rpsS (A0QSD5), rpsT (A0R102), rplV (O06115), rplB (A0QSD4), rplW (A0QSD3), rplM (A0QSP8), and rplE (A0QSG1), as well as chaperonins (A0QQU5), the DNA-binding protein HupB (Q9ZHC5), ATP-binding proteins (I7G6W0), and several metabolic enzymes, including methanol:N,N-dimethyl-4-nitrosoaniline oxidoreductase (A0A2U9PZ50), ATP synthase (A0R200), biotin-dependent acyl-coenzyme A carboxylase (I7G6G9), ribulokinase (Q9LBQ3), L-erythrulose 1-kinase (A0R758) and isocitrate lyase (I7G4E1). In contrast, several enzymes, predominantly dehydrogenase enzymes, were more abundant in the D-form hLF 1–11-treated group than in the untreated control. For example, L-glutamate ϒ-semialdehyde dehydrogenase (A0A2U9PVX0), glyceraldehyde-3-phosphate dehydrogenase (A0A2U9PQ15), acetyl-CoA acetyltransferase (I7FRX4), fumarase (I7GDR8), aldehyde dehydrogenase (NAD (+)) (A0QT96), isocitrate dehydrogenase (NADP) (A0QSZ3) and erythritol/L-threitol dehydrogenase (A0R758). A total of 38 significantly different proteins (*p* value < 0.05) between the untreated control and D-form hLF 1–11-treated groups are summarized in Table S2.

**Figure 7:**
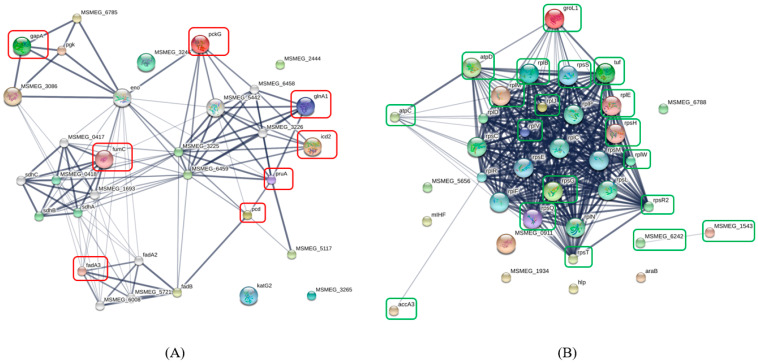
The protein–protein–chemical interaction (PPCI) network analysis of the upregulated proteins (**A**) and downregulated proteins (**B**) of *M. smegmatis* after D-form hLF 1–11 exposure. The PPCI network generated using the STITCH database identified eight upregulated and 19 downregulated proteins of *M. smegmatis* following D-form hLF 1–11 treatment. The network highlights interactions among proteins possibly involved in central carbon metabolism, lipid- and acetyl-CoA-dependent pathways, nitrogen assimilation, redox balance, oxidative stress defense, energy production, translation, and cell growth. Nodes represent individual proteins, whereas edges indicate protein–protein interactions, with line thickness proportional to confidence scores. Upregulated proteins are highlighted with red boxes, and downregulated proteins are highlighted with green boxes.

The PPCI network proteins of *M. smegmatis* following D-form hLF 1–11 treatment were constructed using the STITCH database [15]. The analysis highlighted significant relationships among both upregulated and downregulated proteins. Of these 38 identified proteins, eight of 12 upregulated proteins in the D-form hLF 1–11-treated group were associated with glycolysis, gluconeogenesis, nitrogen metabolism, oxidative TCA cycle, and lipid metabolism (Figure 7A). In contrast, 19 of 26 downregulated proteins were strongly associated with translation, protein folding, and energy production (Figure 7B).

Abbreviations: icd2, isocitrate dehydrogenase [NADP]; glnA1, glutamine synthetase; pruA, L-glutamate ϒ-semialdehyde dehydrogenase; pckG, phosphoenolpyruvate carboxykinase; pcd, aldehyde dehydrogenase (NAD(+)); fumC, fumarate hydratase class II; gapA, glyceraldehyde-3-phosphate dehydrogenase; fadA3, acetyl-CoA acetyltransferase; katG2, catalase-peroxidase 2; rps, ribosomal protein small subunit (rpsG, rpsQ, rpsH, rpsR2, rpsS, and rpsT); rpl, ribosomal protein large subunit (rplV, rplB, rplW, rplM, rplJ, and rplE); atp, ATP synthase subunit (atpC, atpD); tuf, elongation factor Tu; and gloL1, chaperonin-60.

## 3. Discussion

Tuberculosis remains one of the leading causes of infectious-disease-related mortality worldwide. Currently, MDR and XDR TB represent major global health challenges, highlighting the urgent need for new therapeutic strategies. Antimicrobial peptides (AMPs) have emerged as promising therapeutic candidates for combating antimicrobial-resistant pathogens. The hLF 1–11 is a short cationic AMP derived from the N-terminal region of human lactoferrin. The antimicrobial activity of hLF 1–11 against bacteria, fungi, and viruses has been previously reported [16,17,18].

Recently, the antimycobacterial activity of D-form hLF 1–11 against drug-susceptible and MDR *M. tuberculosis*, as well as non-tuberculous mycobacteria, was investigated in our laboratory. Notably, D-form hLF 1–11 exhibited an additive effect in combination with isoniazid and a synergistic effect when combined with rifampicin against *M. tuberculosis* H37Rv [12]. Proteomic analysis of *M. tuberculosis*-infected alveolar macrophages treated with D-form hLF 1–11-loaded niosomes revealed the upregulation of two key host proteins: complement component 6 (C6), a component of the classical complement pathway, and insulin-like growth factor 1 receptor (IGF1R), which is involved in inflammatory signaling [13]. Increased expression of C6 may promote formation of the membrane attack complex (MAC), leading to microbial membrane disruption and lysis [19]. In addition, activation of IGF1R can stimulate TNF receptor-associated factor 6 (TRAF6), a central mediator of inflammatory responses that plays an important role in autophagy induction in mycobacteria-infected host cells [20]. Although the host-directed mechanisms of D-form hLF 1–11-loaded niosomes in *M. tuberculosis*-infected macrophages have been partially elucidated, the direct antimycobacterial mechanism of D-form hLF 1–11 remains unexplored.

Because *M. smegmatis* shares more than 2800 homologous protein-coding genes with *M. tuberculosis*, this fast-growing and non-pathogenic mycobacterial species was employed as a surrogate model in the present study. *M. smegmatis* is widely used for investigating mycobacterial physiology, evaluating drug susceptibility, elucidating mechanisms of action of antimycobacterial agents, and supporting genetic engineering applications [14]. Initially, the MIC of D-form hLF 1–11 against *M. smegmatis* was determined using the REMA, yielding an MIC value of 400 µg/mL. Interestingly, D-form hLF 1–11 exhibits potent antimycobacterial activity against *M. tuberculosis* at low micromolar concentrations, whereas a substantially four-fold higher concentration is required to inhibit *M. smegmatis* [12]. This differential susceptibility may be attributed to species-specific differences in cell envelope, lipid composition, and membrane permeability, which influence peptide penetration and activity [21,22]. Regarding toxicity concerns, although relatively high concentrations were required to achieve antimycobacterial activity against *M. smegmatis*, previous data demonstrated that D-form hLF 1–11 exhibited no detectable hemolytic activity against red blood cells, even at concentrations up to 4000 µg/mL [10], indicating favorable biocompatibility with mammalian cell membranes. Furthermore, D-form hLF 1–11-loaded niosomes showed no cytotoxic effects toward rat alveolar macrophages at the MIC [13]. Consistent with these findings, cytotoxicity evaluation using the human alveolar epithelial cell line (A549) revealed no detectable cytotoxicity, even at concentrations up to 1000 µg/mL (Figure S2).

To evaluate bactericidal activity, live/dead fluorescent staining was subsequently performed. Compared with the untreated bacterial control, D-form hLF 1–11-treated cells exhibited increased PI incorporation, indicating compromised membrane integrity and mycobactericidal activity. Therefore, the bacterial killing activity of D-form hLF 1–11 using live/dead fluorescent staining remains an indirect assumption. Further confirmation using the gold-standard colony-forming unit (CFU) enumeration assay is required to distinguish between bactericidal and bacteriostatic activity. Time–kill kinetic assays further demonstrated that treatment with sub-MICs (25–200 µg/mL) resulted in transient growth inhibition during the logarithmic growth phase. In contrast, higher concentrations (≥400 µg/mL) maintained OD values near baseline throughout the incubation period, indicating pronounced growth suppression. Collectively, these findings demonstrate a dose-dependent antimycobacterial effect of D-form hLF 1–11 against *M. smegmatis*.

To preliminarily investigate the mechanism of action of the peptide in mycobacteria, SEM was performed to examine morphological alterations in *M. smegmatis* following D-form hLF 1–11 treatment. Compared with the untreated control, which displayed well-defined morphology and characteristic cell aggregation, peptide-treated cells exhibited marked structural abnormalities, including distorted cell morphology, apparent cell fusion, and the presence of small spherical structures. Previous SEM analysis of *M. avium* treated with hLF 1–11 demonstrated increased surface roughness and extracellular material accumulation [23]. The differences observed between these studies may be attributable to variations in bacterial species and treatment duration. To further confirm membrane disruption in mycobacteria following exposure to D-form hLF 1–11, additional analyses such as transmission electron microscopy (TEM), the membrane potential assay, and membrane leakage assay would be valuable for elucidating its membrane-disruptive effects [24]. Peptide localization within bacterial cells was visualized using high-resolution fluorescence microscopy. FITC-conjugated D-form hLF 1–11 enabled direct observation of peptide localization in *M. smegmatis*. Bacterial nucleic acids were stained with DAPI, whereas membrane disruption was assessed using the membrane-impermeant intercalating dye PI. Over time, increased accumulation of FITC-D-form hLF 1–11 was observed both on the membrane and within the intracellular compartment. These findings suggest that D-form hLF 1–11 disrupts membrane integrity and possibly translocates into bacterial cells. Several previous studies support this observation. Biophysical analyses have demonstrated that hLF 1–11 interacts with membrane-mimicking environments, indicating that its antimicrobial activity is mediated, at least in part, through membrane disruption [25]. Moreover, hLF 1–11 has been shown to bind specifically to lipid A of lipopolysaccharide in Gram-negative bacteria, suggesting targeted interactions with bacterial membranes [26]. Huo and colleagues demonstrated, using an electrophoretic gel mobility shift (EMSA) assay, that hLF 1–11 is capable of binding to double-stranded DNA. It has been hypothesized that hLF 1–11 would translocate across the cytoplasmic membrane and interact with intracellular DNA [27]. More recently, hLF 1–11 treatment of KPC-producing *K. pneumoniae* was reported to induce transient membrane depolarization and increased membrane permeability, further supporting its membrane-disruptive properties [28]. Although our data support the presence of intracellular target(s) of D-form hLF 1–11, as indicated by the intracellular accumulation of the fluorescent peptide, this finding alone is insufficient to definitively identify a specific intracellular target. Additional mechanistic studies, including nucleic acid- or protein-binding assays, transcriptomic analysis, and intracellular enzyme inhibition assays, are required to confirm intracellular activity [24,29].

The cellular response of *M. smegmatis* to D-form hLF 1–11 exposure was further investigated using proteomic analysis. Following protein extraction, the total protein concentration in peptide-treated groups was significantly lower than that in untreated controls. To ensure that the observed proteomic differences reflected biological regulation rather than differences in biomass, protein concentrations from treated and untreated groups were normalized prior to enzymatic digestion and LC–MS analysis. Variations in protein loading have previously been reported to confound comparative proteomic analyses [30]. Following LC–MS analysis, only 223 protein groups were identified. The relatively limited proteomic depth may be attributed to several factors. First, the protein database available for *M. smegmatis* is less comprehensive than those of more extensively studied mycobacterial species, such as *M. tuberculosis* and *M. avium*, which may restrict protein identification coverage. Second, the use of data-dependent acquisition (DDA) mode may have limited the detection of low-abundance proteins, as precursor ion selection in DDA is generally biased toward highly abundant peptides [31]. In addition, the complex cell wall structure and high lipid content of mycobacteria may reduce protein extraction efficiency and peptide recovery, thereby affecting overall proteomic coverage [32].

Exposure to D-form hLF 1–11 markedly altered several metabolic pathways in *M. smegmatis*, particularly central carbon metabolism. Increased abundance of key metabolic enzymes, including glyceraldehyde-3-phosphate dehydrogenase (GAPDH), isocitrate dehydrogenase (NADP-dependent), fumarate hydratase, and phosphoenolpyruvate carboxykinase (PEPCK), was observed. GAPDH is a central glycolytic enzyme that regulates carbon flux and generates NADH, thereby linking carbon metabolism with energy production. Isocitrate dehydrogenase and fumarate hydratase are integral tricarboxylic acid (TCA) cycle enzymes involved in maintaining carbon flow and energy generation, whereas PEPCK is a key gluconeogenic enzyme that reflects metabolic flexibility. Collectively, these coordinated responses suggest enhanced glycolytic activity to meet elevated energy demands and/or a metabolic shift toward gluconeogenesis to conserve carbon under antimicrobial stress. GAPDH has previously been reported to undergo regulatory modulation during oxidative and metabolic stress conditions [33].

Consistent with SEM and fluorescence microscopy findings, membrane disruption induced by D-form hLF 1–11 was associated with increased abundance of acetyl-CoA acetyltransferase and decreased abundance of biotin-dependent acyl-CoA carboxylase. In mycobacteria, acetyl-CoA functions not only as an energy metabolite but also as a precursor for lipid biosynthesis, which is essential for maintaining the integrity of the lipid-rich mycobacterial cell envelope [34]. These changes likely reflect adaptive remodeling of acetyl-CoA-dependent lipid metabolism to support membrane repair and cell envelope restructuring following peptide-induced damage. Oxidative stress and lipid metabolism can generate toxic aldehyde intermediates; accordingly, increased expression of methanol dehydrogenase and NAD+-dependent aldehyde dehydrogenase highlights the importance of aldehyde detoxification and redox regulation [35]. Efficient oxidation of aldehydes not only prevents cellular damage but also contributes to the regeneration of NADH or NADPH, thereby supporting energy production and redox homeostasis. Furthermore, induction of catalase-peroxidase 2 (KatG2) and chaperonin-60 indicates activation of oxidative stress defense pathways in response to D-form hLF 1–11 exposure. KatG2 is a critical component of the oxidative stress response system in *M. smegmatis*, functioning to decompose hydrogen peroxide and organic peroxides, thereby protecting cells from reactive oxygen species (ROS) [36]. Its functional association with NADPH-producing enzymes, such as isocitrate dehydrogenase, further supports protection against oxidative damage. The concurrent upregulation of aldehyde dehydrogenases reinforces this protective network by eliminating reactive aldehydes generated during oxidative stress and lipid peroxidation. Nitrogen metabolism is also closely linked to central carbon metabolism in *M. smegmatis*. Glutamine synthetase catalyzes ATP-dependent ammonia incorporation into glutamine, serving as a major nitrogen donor for numerous biosynthetic pathways [37]. L-glutamate-γ-semialdehyde dehydrogenase connects glutamate and proline metabolism. Coordinated regulation of these enzymes reflects integration of nitrogen assimilation and amino acid biosynthesis under peptide-induced stress conditions.

Two proteins, dienelactone hydrolase and the transcriptional regulatory protein PdtaR, displayed markedly increased fold changes following D-form hLF 1–11 exposure, with approximately 120-fold and 32,000-fold increases, respectively. Although these proteins were not interconnected within the PPCI network generated using the STITCH database, their functional roles are well established. In *M. smegmatis*, dienelactone hydrolase catalyzes the hydrolytic cleavage of dienelactone, a key intermediate generated during the degradation of aromatic compounds [38]. This process contributes to the detoxification of reactive intermediates and incorporation of carbon derived from environmental aromatics into central metabolic pathways, including the TCA cycle. PdtaR is a phosphorylation-dependent response regulator containing an ANTAR RNA-binding domain that modulates gene expression at the post-transcriptional level [39]. As a component of the PdtaS–PdtaR two-component regulatory system, it plays an essential role in adaptation to nutrient limitation by regulating central carbon metabolism and biosynthetic pathways [40]. Collectively, the upregulation of these proteins suggests coordinated metabolic adaptation that supports both energy production and biomass maintenance under stress conditions. A summary of upregulated proteins in *M. smegmatis* following D-form hLF 1–11 exposure is presented in Figure 7A.

Proteins involved in translation and energy production, including elongation factor Tu (EF-Tu), multiple ribosomal subunit proteins (rpsG, rpsQ, rpsH, rpsR2, rpsS, rpsT, rplV, rplB, rplW, rplM, rplJ, and rplE), and ATP synthase subunits beta and epsilon, were abundantly detected in untreated control samples, reflecting active protein synthesis and bacterial growth. Coordination between translational machinery and metabolic enzymes ensures that biosynthetic capacity is aligned with available energy and nutrients. Downregulation of these proteins following D-form hLF 1–11 treatment indicates suppression of global biosynthetic activity and reduced abundance of proteins associated with amino acid biosynthesis, energy production, translation, and cell growth [41]. The PPCI network of downregulated proteins following D-form hLF 1–11 treatment is shown in Figure 7B.

Although the exposure to D-form hLF 1–11 markedly altered several metabolic pathways in *M. smegmatis*, additional studies should be performed to confirm the metabolic alterations induced by the peptide, such as ROS quantification, ATP measurements, metabolomic and lipidomic analysis, and RT-qPCR validation of specific transcripts [42,43,44].

Overall, SEM and fluorescence imaging analysis demonstrated that D-form hLF 1–11 induces membrane perturbation and possible peptide translocation across the mycobacterial membrane. Proteomic profiling further revealed alterations in coordinated networks involving central carbon metabolism, alternative carbon utilization, nitrogen and amino acid biosynthesis, redox homeostasis, oxidative stress defense, and global growth regulation. These changes ultimately resulted in suppression of biosynthetic activity and reduced abundance of proteins involved in amino acid biosynthesis, energy production, translation, and cell growth. Interestingly, several differentially expressed proteins identified in this study were consistent with previous proteomic analyses of *M. smegmatis* treated with anti-tuberculosis drugs such as isoniazid and ethambutol [45]. These findings suggest that similar responses may also occur in pathogenic mycobacteria, including members of the *M. tuberculosis* complex. Therefore, further proteomic investigation of D-form hLF 1–11 against *M. tuberculosis* is warranted.

## 4. Materials and Methods

### 4.1. Bacterial Strain and Antimicrobial Peptides

*Mycobacterium smegmatis* ATCC 14468 was purchased from Biomedia company (Biomedia (Thailand) Co., Ltd., Nonthaburi, Thailand). The bacteria were initially grown on Lowenstein–Jensen (LJ) medium and subsequently subcultured in Middlebrook 7H9 broth supplemented with oleic acid–albumin–dextrose–catalase (OADC) (BD BBL Middlebrook OADC Enrichment, Becton Dickinson, Sparks, MD, USA) and incubated at 37 °C for 3 days. The D-form of hLF 1–11, FITC-conjugated D-form hLF 1–11, and L-form of AK15-6 [46] were obtained from Synpeptide (Synpeptide Co., Ltd., Shanghai, China). Peptides with the purity > 95% were dissolved in sterile PBS (pH 7.0), filtered through a 0.2 μm syringe filter (Sartorius, Göttingen, Germany), aliquoted, and stored at –20 °C.

### 4.2. Antimycobacterial Activity Testing

The antimycobacterial activity of the AMPs was assessed using a protocol adapted from the Clinical and Laboratory Standards Institute (CLSI) M24-A2 guidelines [47]. Briefly, two-fold serial dilutions of each peptide prepared in cation-adjusted Mueller–Hinton broth (CAMHB) were incubated with *M. smegmatis* ATCC 14468 (final concentration ~5 × 10^5^ CFU/mL) under ambient atmospheric conditions for 3 days. Bacterial, peptide, and media controls were included in each experiment, and AK15-6 served as the positive control. In accordance with the CLSI guidelines, moxifloxacin was also included in this study to ensure standardized susceptibility testing and reliable interpretation of antimicrobial activity. Following incubation, 10 µL of 0.02% resazurin (Sigma-Aldrich, Darmstadt, Germany) was added, and plates were incubated overnight at 37 °C. A color shift from blue (resazurin) to pink (resorufin) indicated bacterial growth. The MIC was defined as the lowest peptide concentration that prevented this color change. All experiments were performed in duplicate and repeated independently three times.

### 4.3. Live–Dead Fluorescent Staining

The bactericidal activity of D-form hLF 1–11 against *M. smegmatis* was evaluated using SYTO 9 and PI co-fluorescent staining. SYTO 9 is a green fluorescent nucleic acid dye that labels all bacterial cells, whereas PI is a red fluorescent dye that penetrates only cells with compromised membranes, thereby indicating cell death. Briefly, log-phase *M. smegmatis* cultures (1 × 10^8^ CFU/mL) were treated with 2 × MIC of D-form hLF 1–11 and incubated at 37 °C for 1 h. AK15-6 served as the positive control, while cells treated with the peptide-solubilizing buffer alone served as the negative control. Following incubation, bacterial cells were harvested, washed twice with sterile PBS, and resuspended in 1 mL of sterile PBS. Fluorescent staining was performed by incubating the cells with SYTO 9 (3.34 mM) and PI (20 mM) (Lumiprobe Ltd., Wan Chai, Hong Kong) in the dark at room temperature for 15 min, as described by Deng et al. [48]. Stained cells were washed twice with 1 mL of sterile PBS, and centrifuged. The resulting pellet was resuspended in 50 µL of ProLong™ Glass Antifade Mountant (Invitrogen by Thermo Fisher Scientific, Hillsboro, OR, USA), applied onto a microscope slide, and visualized using a Zeiss Apotome 3 fluorescence microscope (Carl Zeiss Microscopy GmbH, Jena, Germany). The public-domain software ImageJ version 1.44 was used to quantify bacterial cell counts and measure the fluorescence intensities of SYTO 9- and PI-labeled cells. A 435 × 435-pixel region of interest (ROI) was defined for analysis. Approximately 1000 cells per condition were evaluated from 10 to 15 randomly captured images. Differences in cell death or viability, expressed as both the percentage of cell counts and fluorescence intensities, were statistically analyzed based on the corresponding *p*-values [49]. The statistical significance was defined as follows: * *p* < 0.05; ** *p* < 0.01, and *** was *p* < 0.0001.

### 4.4. Time–Kill Assay

The time–kill assay of D-form hLF 1–11 against *M. smegmatis* was conducted as follows [50]. A fresh culture of *M. smegmatis* was adjusted to approximately 10^8^ CFU/mL using McFarland standard No. 0.5 (SIA Biosan, Riga, Latvia), then diluted to 10^6^ CFU/mL and incubated with variation concentrations of D-form hLF 1–11; the final concentrations ranged from 25 to 1600 µg/mL. The 96-well plates were incubated at 37 °C for 3 days, and bacterial growth was monitored kinetically at 8 h intervals using an Eon™ Microplate Spectrophotometer (BioTek Instruments Inc., Winooski, VT, USA). Bacterial cell controls and media controls were included in parallel. All assays were performed in duplicate and repeated independently three times. Growth curves were plotted with the means of turbidity at OD 600 nm versus incubation time.

### 4.5. Scanning Electron Microscopy (SEM)

SEM was used to examine surface morphological alterations in *M. smegmatis* following treatment with D-form hLF 1–11. The experimental procedure was adapted from Silva et al. [23]. Log-phase bacteria (~10^8^ CFU/mL), adjusted to McFarland standard 0.5, were exposed to 400 µg/mL (1 × MIC) or 800 µg/mL (2 × MIC) of D-form hLF 1–11 on sterile isopore filters (0.2 µm PC membrane, Merck Millipore Ltd., County Cork, Ireland) placed in 24-well culture plates (Corning Incorporated, Corning, NY, USA) and incubated at 37 °C for 2 days. Untreated bacteria served as a negative control. After incubation, culture supernatants were removed and the membrane-adhered bacteria were fixed with 2.5% glutaraldehyde in 0.1 M sodium cacodylate buffer at 4 °C overnight. Samples were washed twice with 0.1 M sodium cacodylate buffer and post-fixed with 2% osmium tetroxide for 2 h. Dehydration was carried out using an ethanol series (50%, 75%, 85%, 95%, and 100%), each for 10 min at room temperature. The membranes were then dried using a critical point dryer (K850, Quorum Technologies Ltd., East Sussex, UK), sputter-coated with a gold/palladium film, and examined using a JSM-6610LV scanning electron microscope (JEOL Ltd., Tokyo, Japan).

### 4.6. Peptide Localization

Localization of the D-form hLF 1–11 peptide was assessed by fluorescent staining using a method modified from Deng et al. [48] and Phillips et al. [51]. *M. smegmatis* was cultured in CAMHB and incubated at 37 °C for 1–2 days. Cells were collected by centrifugation at 6000× *g* for 15 min and washed twice with sterile phosphate-buffered saline (PBS; pH 7.4). The pellet was resuspended in CAMHB and adjusted to McFarland standard No. 0.5. Approximately 10^8^ CFU/mL of *M. smegmatis* was then incubated in the dark with 1600 µg/mL (4 × MIC) of FITC-conjugated D-form hLF 1–11 at 37 °C for either 10 min or 1 h, with gentle mixing every 15 min. *M. smegmatis* treated with the peptide-solubilizing buffer for 1 h served as the negative control. Following incubation, cells were washed twice with sterile PBS and stained with 1.5 µL of DAPI (1 µg/mL) for 15 min at room temperature in the dark. After another wash with PBS, cells were stained with 1.5 µL of membrane-impermeant PI (20 mM) and incubated under the same conditions for 15 min. Finally, the cells were washed twice with PBS, resuspended in 50 µL of ProLong™ Glass Antifade Mountant (Invitrogen by Thermo Fisher Scientific, OR, USA), and imaged using a Zeiss Apotome 3 fluorescence microscope (Carl Zeiss Microscopy GmbH, Jena, Germany). Interpretation was performed as previously described [52].

### 4.7. Proteomic Analysis

The mechanism of action of D-form hLF 1–11 against *M. smegmatis* was assessed by proteomic analysis. Fresh culture of bacteria was adjusted to McFarland No. 4 (1.2 × 10^9^ CFU/mL) and incubated with 400 µg/mL for 20 h. Bacterial cells incubated with peptide solubilization buffer were used as the cell control. Bacteria were examined in three independent experiments. Bacteria were centrifuged at 5000 rpm for 20 min, washed twice with sterile PBS and pelleted by centrifugation. Cell pellets were added to 100 µL of 50 mM ammonium bicarbonate (ABC) buffer and homogenized using metal beads in a tissue lyser (SWE-FP, Wuhan Servicebio Technology Co., Ltd., Wuhan, Hubei, China) for 3 min at 50 Hz twice. The protein concentration of each sample was measured using the Bradford assay and normalized to 150 µg/mL. Protein solution was reduced with 100 mM dithiothreitol (DTT) at 65 °C for 30 min and alkylated with 500 mM iodoacetamide (IAA) at room temperature (RT) in the dark for 20 min. Proteins were digested with 2.5 µL of trypsin (0.1 mg/mL) at 37 °C overnight. The digestion reaction was terminated by adding 10% formic acid (FA) and centrifuged at 14,000 rpm for 10 min. The supernatant was collected and transferred to an LC-MS vial for subsequent LC-MS analysis.

### 4.8. LC-MS/MS Acquisition

Peptide samples were analyzed using Agilent 1290 Infinity II LC system coupled with an Agilent 6545XT Q-TOF mass spectrometer (Agilent Technologies, Inc., Santa Clara, CA, USA). The procedure was followed as previously described [53,54]. The LC separation was conducted on Agilent peptide mapping column (120 Å, 2.1  ×  150 mm, 2.7 µm) at 60 °C. The injection volume was 20 µL. Mobile phase A was 0.1% FA in water and mobile phase B was 0.1% FA in acetonitrile. The LC gradient was set as follows, 0% B for 2 min, 0–20% B in 33 min, 20–30% B in 20 min, 30–50% B in 10 min, 50–90% B in 5 min, 90% B for 5 min, 90–0% B in 5 min, and 0% B for 5 min, with a constant flow rate of 0.4 mL/min. MS analysis was conducted in positive ionization mode with a mass range of 100–1700 m/z. MS parameters were set as follows: gas temperature at 325 °C, nebulizer at 35 psi, dying gas at 13 L/min, sheath gas temperature at 350 °C, sheath gas flow at 12 L/min, capillary voltage at 4000 V, nozzle voltage at 500 V, fragmentor voltage at 175 V and skimmer voltage at 65 V. Acquisition time was 1 spectrum per s. A maximum of 2 precursor ions per cycle were selected for MS/MS fragmentation. Collision energy (CE) was varied according to the charge state of the peptide. For peptides with charge + 1 and + 2, the CE was calculated using a formula of (3.1 × ((*m*/*z*)/100) + 1), while for peptides with charge >+ 3, the CE was calculated using a formula of (3.6 × ((*m*/*z*)/100) − 4.8).

### 4.9. Data Processing

Raw LC-MS/MS data were converted to mzXML format using MSConvert (ProteoWizard version 3.0.23299) and OpenMS software version 3.0.0. Database searching and label-free quantification were performed using MaxQuant software version 2.6.3. Spectra were searched against the UniProt *Mycobacterium smegmatis* protein database (taxonomy ID: 1772). Trypsin was specified as the proteolytic enzyme. Carbamidomethylation of cysteine residues was set as a fixed modification, while N-terminal protein acetylation and methionine oxidation were included as variable modifications. The false discovery rate (FDR) for both peptide spectrum matches and proteins was set to 10% (0.1). Label-free quantification (LFQ) was enabled with a minimum ratio count of 1. Proteins identified only by site or reverse sequences were excluded from further analysis. Potential contaminant proteins were also removed. In total, 223 protein groups were confidently identified. The LFQ intensity data were log10-transformed and mean-centered to reduce right skewness and to achieve homoscedasticity. The transformed data were used for principal component analysis (PCA), partial least squares discriminant analysis (PLS-DA), and hierarchical clustering heatmap construction using the online MetaboAnalyst version 6.0 software. Features with all zero values or a single non-zero value across samples (n = 38) were excluded. Remaining zero values were imputed using one-fifth of the minimum positive value for each variable. Volcano plots and Student’s *t*-test were also performed with MetaboAnalyst software to assess proteome change between control and MIC peptide treatment.

## 5. Conclusions

In summary, this study provides new mechanistic insights into the antimycobacterial activity of D-form hLF 1–11 using *M. smegmatis* as a model organism. Exposure to sub-MICs resulted in only transient growth inhibition, highlighting the importance of adequate peptide concentrations for sustained antimicrobial efficacy. SEM analysis and fluorescence-based peptide localization supported a mechanism involving membrane perturbation and possible intracellular targeting. Proteomic analysis revealed extensive metabolic alterations in central carbon metabolism, lipid- and acetyl-CoA-dependent pathways, nitrogen assimilation, redox balance, and oxidative stress defense. These coordinated responses likely reflect an adaptive response of *M. smegmatis* to peptide-induced envelope damage, metabolic stress, and oxidative injury. However, D-form hLF 1–11 ultimately suppressed global biosynthetic activity, including proteins involved in energy production, translation, amino acid biosynthesis, and cell growth. Collectively, these findings suggest that D-form hLF 1–11 exerts antimycobacterial activity against *M. smegmatis* through membrane disruption and possible intracellular interference. Ultimately, although *M. smegmatis* shares numerous homologous protein-coding genes with *M. tuberculosis* and has been widely used as model organism for antimycobacterial drug discovery, substantial biological differences exist between the two species, particularly with respect to pathogenicity- and virulence-associated genes in *M. tuberculosis*. Unlike the non-pathogenic and predominantly extracellular *M. smegmatis*, *M. tuberculosis* is an intracellular pathogen capable of surviving and replicating within host cells. Hence, further studies will be conducted to evaluate the ability of D-form hLF 1–11 to penetrate host cell membranes, reach intracellular compartments such as phagosomes, and exert antimycobacterial activity in infected cells and animal models. These investigations will be crucial for determining the therapeutic potential of D-form hLF 1–11 against *M. tuberculosis*, including drug-resistant strains.

## Data Availability

The proteomic raw data have been deposited in ProteomeXchange under the accession number PXD073501 and the jPOST repository under project ID JPST004349. (The URL for reviewer: https://repository.jpostdb.org/preview/49508610269798554bc155 (accessed on 24 January 2026); access key: 7281).

## Supplementary Materials

The following supporting information can be downloaded at: https://www.mdpi.com/article/10.3390/antibiotics15060607/s1, Figure S1. PCA and PLS-DA Analyses; Figure S2. Cytotoxicity assessment of D-form and L-form hLF 1–11 against human alveolar epithelial cell lines (A549 cell lines); Table S1. A total of 223 protein groups detected in this study; Table S2. Summary of 38 significantly different proteins (*p* value < 0.05) in bacterial control group and D-form hLF 1-11 treated group.
