# Supplementary material for: Elucidation of the Antimycobacterial Activity of D-Form Human Lactoferricin 1–11 (D-Form hLF 1–11) Against Mycobacterium smegmatis Through Proteomics and Imaging Analysis
Source: Antibiotics (Basel). 2026 Jun 15;15(6):607. doi: 10.3390/antibiotics15060607 (PMC13296235; doi:10.3390/antibiotics15060607)
Supplement: Supplementary file 1 [file antibiotics-15-00607-s001.zip › supplementary file-Table S1.pdf]

Table S1. A total of 223 protein groups detected in this study

	Selected Protein I	Protein IDs
1	A0A2U9PZ50	A0A2U9PZ50;I7GAD7;A0R5M3;A0A653FAI
2	A0A2U9PVX0	A0A2U9PVX0;I7GET1;A0R2H8;A0A653FMN
3	A0QQU5	A0QQU5;A0A2U9PJL5;G1EFP8;A0A0A1EIU
4	I7G686	I7G686;A0QT50;A0A2U9PLU0
5	A0QSS3	A0QSS3;A0A2U9PLG7
6	A0A2U9PQI5	A0A2U9PQI5;A0QWW2;Q9LBS3
7	A0A2U9PJ55	A0A2U9PJ55;A0QQC8;E5DMV1
8	A0R3M4	A0R3M4;A0A2U9PX87
9	A0A2U9PYX5	A0A2U9PYX5;A0A653FAC7;A0R5G1
10	I7G5V6	I7G5V6;A0A2U9PLC0;A0QSN7;I7G4D2;A0
11	Q9LBQ3	Q9LBQ3;I7F995;A0QT51;A0A653FKM4;A0
12	A0A2U9PKX4	A0A2U9PKX4;A0QS98;A1C3J4
13	A4ZHR8	A4ZHR8;A0A2U9PM45
14	I7FRX4	I7FRX4;A0R2Y1;A0A653FET9;A0A2U9PWH
15	A0A2U9PNL7	A0A2U9PNL7;I7FZT6;A0QUV7
16	Q9ZHC5	Q9ZHC5;Q4PLR8;A0A2U9PNN7
17	I7GAH5	I7GAH5;A0QWY0;A0A653FA46;A0A2U9PQ
18	I7G2C2	I7G2C2;A0QNF6;A0A2U9PH26
19	I7GDR8	I7GDR8;A0R2U8;A0A653FG35;A0A2U9PW
20	A0R0B3	A0R0B3;A0A2U9PTY7
21	A0R3B8	A0R3B8;A0A2U9PWX2
22	I7G5C1	I7G5C1;A0QS63;A0A2U9PL09
23	A0QVB9	A0QVB9;A0A2U9PNW9
24	A0QT96	A0QT96;I7G4T8;A0A2U9PLW1
25	I7G6A9	I7G6A9;A0QUV6;A0A2U9PNT6
26	A0QSZ3	A0QSZ3;I7FYJ4;A0A2U9PLP3
27	A0R200	A0R200;A0A2U9PVD8
28	I7GGP4	I7GGP4;A0R761;A0A653FA40;A0A2U9Q0V
29	I7G6G9	I7G6G9;A0QTE1;A0A2U9PMC8
30	Q9KGW0	Q9KGW0;A0R5E1;A0A2U9Q0I8
31	Q9RHG2	Q9RHG2;A0QT53;A0A2U9PM48
32	A0R006	A0R006;A0A2U9PTH0
33	A0QWS8	A0QWS8;A0A2U9PQE2
34	A0QYY6	A0QYY6;A0A2U9PSM7;U5U3X6;V5W3M9
35	A0R202	A0R202;A0A2U9PVH1
36	I7G2U5	I7G2U5;A0QR68;A0A2U9PNC7
37	P41193	P41193;A0QS97;A0A2U9PKX8
38	A0QSD4	A0QSD4;A0A2U9PL18
39	A0R757	A0R757;A0A2U9Q0H5;A0QXE5;A0A2U9PR
40	A0R758	A0R758;A0A653F837;A0A2U9Q0G2
41	I7FSI8	I7FSI8;A0R3M3;A0A2U9PX88
42	I7FFV0	I7FFV0;A0QZ83;A0A2U9PSY2
43	A0QVQ5	A0QVQ5;A0A2U9PPG3;U5U0Z3
44	A0A2U9PKY1	A0A2U9PKY1;A0QSA8;I7G3X8
45	I7FBH2	I7FBH2;A0QV51;A0A2U9PNR0

46	I7G9R3	I7G9R3;A0QXZ5;A0A2U9PRU9
47	I7FEK8	I7FEK8;A0QY23;A0A653FMV3;A0A2U9PRU
48	I7GF12	I7GF12;A0R2V6;A0A653FE97;A0A2U9PWA
49	Q9AGJ6	Q9AGJ6;A0QP32;A0A2U9PHP3
50	Q939T2	Q939T2;A0R079;A0A2U9PTN9
51	A0QSG4	A0QSG4;A0A2U9PL58
52	A0QUX8	A0QUX8;A0A2U9PNR1
53	I7FGD9	I7FGD9;A0QSG5;A0A2U9PL48
54	I7FMR4	I7FMR4;A0QYD5;A0A2U9PS55
55	A0R609	A0R609;A0A2U9PZI9;P0C580;Q9LBD6
56	I7G4N0	I7G4N0;A0QT42;A0A2U9PLT3
57	I7G8I0	I7G8I0;A0R417;A0A2U9PXH6;Q9RMR0
58	A0QSL8	A0QSL8;A0A2U9PLK5
59	I7FL78	I7FL78;A0R4B3;A0A653FC62;A0A2U9PXS3
60	I7FGZ1	I7FGZ1;A0R0A1;A0A653FCX4;A0A2U9PTQ
61	A0QX20	A0QX20;A0A653FBS0;A0A2U9PQM4
62	I7G6M8	I7G6M8;A0QV52;A0A2U9PNU5
63	A0QWW3	A0QWW3;A0A2U9PQJ9
64	A0R2X1	A0R2X1;A0A2U9PWK5
65	I7FI17	I7FI17;A0R1B3;A0A653FD79;A0A2U9PUR
66	Q7WYN1	Q7WYN1;A0QWT3;A0A2U9PQG3
67	A0QS62	A0QS62;A0A2U9PKW5
68	A0QSP9	A0QSP9;A0A2U9PLP0
69	A0QS46	A0QS46;A0A2U9PKS3
70	Q9RP36	Q9RP36;A0QYG2;A0A2U9PS38
71	A0QWX9	A0QWX9;A0A2U9PQJ2
72	A0QSD1	A0QSD1;A0A2U9PL03
73	I7G4E1	I7G4E1;A0QQX6;A0A2U9PJP3
74	A0QSL7	A0QSL7;A0A2U9PL94
75	I7G7Z8	I7G7Z8;A0R3N8;A0A653F9R4;A0A2U9PX3
76	A0A2U9PUU3	A0A2U9PUU3;I7FQK1;A0R1A7;A0A653FBE
77	A0R1V9	A0R1V9;Q57529;A0A2U9PVH6
78	P53649	P53649;A0R652;A0A653F936;A0A2U9PZL7
79	A0A2U9PSB5	A0A2U9PSB5;A0QYG3;Q9RP37
80	A0QV10	A0QV10;A0A653FE78;A0A2U9PNM4
81	A0QSL5	A0QSL5;A0A2U9PL95
82	I7FEX4	I7FEX4;A0QYF7;A0A653FFH9;A0A2U9PSA
83	A0QP20	A0QP20;I7FVV1;A0A2U9PHQ2
84	A0R7F9	A0R7F9;A0A2U9Q0X2
85	O06114	O06114;A0QSD2;A0A2U9PL07
86	A0QXX7	A0QXX7;A0A653FN71;A0A2U9PRT0
87	A0QV37	A0QV37;A0A653FFJ7;A0A2U9PNU6
88	A0QSG0	A0QSG0;A0A2U9PL60
89	A0A2U9PHC6	A0A2U9PHC6;A0QNM4;I7F4L2;A0A653FPE
90	Q7WYH3	Q7WYH3;I7GEQ6;A0R2E3;A0A2U9PW26
91	I7G1P3	I7G1P3;A0QPE7;A0A2U9PHZ5
92	A0QYU6	A0QYU6;A0A2U9PSH6

93	A0QSD3	A0QSD3;A0A2U9PL26
94	I7F6Q4	I7F6Q4;A0QQN5;A0A2U9PJI1
95	A0QSG1	A0QSG1;A0A2U9PLA3
96	I7FI06	I7FI06;A0R198;A0A2U9PW74
97	A0QSP8	A0QSP8;A0A653FEZ4;A0A2U9PLC7
98	Q9RHG3	Q9RHG3;Q9LBQ2;I7G4P3;A0QT52;A0A2U
99	P00215	P00215;I7G6W3;A0R2I1;A0A2U9PW22
100	I7G9Y1	I7G9Y1;A0QY79;A0A2U9PRW3
101	I7GD73	I7GD73;A0R072;A0A653FAW6;A0A2U9PT
102	A0QYF5	A0QYF5;I7FMS9;A0A2U9PS74
103	A0A2U9PVC8	A0A2U9PVC8;A0R1Y7
104	I7G8G0	I7G8G0;A0A2U9PQK2;A0QWY3;A0A653FA
105	A0QSX4	A0QSX4;I7G631;A0A2U9PLX8
106	A0QSE0	A0QSE0;A0A2U9PL81
107	A0QRN3	A0QRN3;A0A653FG66;A0A2U9PKG4
108	A0QSS4	A0QSS4;A0A653FFK6;A0A2U9PLK8
109	O06115	O06115;A0QSD6;A0A2U9PL12
110	A0QVB8	A0QVB8;A0A2U9PNW3
111	A0QSG3	A0QSG3;A0A2U9PL37
112	A0R1Z9	A0R1Z9;A0A2U9PVE4
113	I7G4Z3	I7G4Z3;A0QRN7;A0A653FGE3;A0A2U9PK
114	A0QVQ3	A0QVQ3;A0A2U9PPF0
115	A0QS45	A0QS45;A0A2U9PKR6
116	A0A2U9PLF2	A0A2U9PLF2
117	A0QVE0	A0QVE0;A0A2U9PP60
118	I7GFG2	I7GFG2;A0R5B0;A0A653FNR6;A0A2U9Q0
119	A0R6E3	A0R6E3;A0A2U9PZT5
120	A0R170	A0R170;I7GCM1;A0A653FBM7;A0A2U9PU
121	I7F8U3	I7F8U3;A0QSN8;A0A653FF04;A0A2U9PLB
122	A0R199	A0R199;A0A653FBE8;A0A2U9PUP9
123	I7GBX4	I7GBX4;A0R0C7;A0A2U9PU03;A0A2U9PIK
124	A0R7F6	A0R7F6;A0A653F9J9;A0A2U9Q132
125	A0R0B2	A0R0B2;A0A2U9PTZ0
126	A0R7F7	A0R7F7;A0A2U9Q139
127	I7FPG4	I7FPG4;A0R7J6;A0A2U9Q112
128	A0R5Z0	A0R5Z0;A0A2U9PZH2
129	A0QSH8	A0QSH8;A0A2U9PLG2
130	A0R0B0	A0R0B0;A0A2U9PTU4
131	I7GFQ8	I7GFQ8;A0R623;A0A2U9PZI7
132	A0QSD0	A0QSD0;A0A2U9PL74
133	A0A2U9PS95	A0A2U9PS95;I7FMU4;A0QYH7
134	I7F8Q0	I7F8Q0;A0QSJ2;A0A653FFU6;A0A2U9PL7
135	I7G4Y4	I7G4Y4;A0QTE3;A0A2U9PM55
136	A0A2U9PLF7	A0A2U9PLF7;A0QSL9;A0A653FF30
137	Q8RME2	Q8RME2;I7F8C1;A4ZHU4;A0A653FM53;A
138	I7GDX1	I7GDX1;A0R197;A0A2U9PUV0
139	A0QSD7	A0QSD7;A0A2U9PLC2

140	Q9AFI5	Q9AFI5;A0A2U9Q0W2
141	A0R597	A0R597;A0A653FM64;A0A2U9PYR7;I7FU5
142	A0A2U9PQP6	A0A2U9PQP6;I7G8L7;A0QX35
143	I7F4Z3	I7F4Z3;A0A653FNY1;A0QNZ3;A0A2U9PHL
144	I7F603	I7F603;A0QPY2;A0A2U9PIQ9
145	I7FEW5	I7FEW5;A0QYE7;A0A2U9PS96
146	P0C558	P0C558;A0R692;A0A2U9PZP1
147	I7GGF4	I7GGF4;A0A2U9PZT1;A0R656
148	A0QSD8	A0QSD8;A0A2U9PL83
149	I7FX95	I7FX95;A0QQW8;A0A653FDR7;A0A2U9PJ[illegible]
150	A0QYU8	A0QYU8;I7GC56
151	A0QSD9	A0QSD9;A0A2U9PL45
152	I7FJZ3	I7FJZ3;A0QVR8;A0A2U9PPI6
153	I7FVQ8	I7FVQ8;A0R760;A0A2U9Q0J3
154	A0R6B7	A0R6B7;A0A653FLD7;A0A2U9PZS4;I7GFU
155	I7G6W0	I7G6W0;A0QTR2;A0A2U9PMG7
156	I7G4K7	I7G4K7;A0QT22;A0A653FJ55;A0A2U9PLQ[illegible]
157	A0R151	A0R151;A0A2U9PUJ1
158	I7GFH6	I7GFH6;A0R5H1;A0A2U9PYU9
159	O30973	O30973;I7FW12;A0R7I9;A0A653F8B3
160	I7G436	I7G436;A0QSG8;A0A653FF82
161	P96803	P96803;A0R3N9;A0A653F9Q7;A0A2U9PX3
162	P0C563	P0C563;A0QS96;A0A2U9PKZ1
163	I7G2H6	I7G2H6;A0QQQ1;A0A2U9PJH5
164	A0R102	A0R102;A0A2U9PUD4
165	A0QQC1	A0QQC1;I7F6E6;A0A2U9PJC2
166	A0QXD8	A0QXD8;A0A2U9PQY6
167	I7GAH8	I7GAH8;A0R5S2;A0A2U9PZB0
168	A0R617	A0R617;I7FMV0;A0A653F966;A0A2U9PZH
169	I7GBA7	I7GBA7;A0QXW3;A0A2U9PRI1
170	A0A2U9Q0F0	A0A2U9Q0F0
171	A0QY95	A0QY95;A0A2U9PTD1;I7G354
172	I7F8K7	I7F8K7;A0QSF2;A0A653FFZ1;A0A2U9PL2[illegible]
173	I7FRM7	I7FRM7;A0R2H7;A0A2U9PVX6
174	A0QSG7	A0QSG7
175	I7GEW9	I7GEW9;A0R4G4;A0A653FC78;A0A2U9PY[illegible]
176	A0QXS8	A0QXS8;I7FE72
177	A0R2K9	A0R2K9
178	I7F4S5	I7F4S5;A0QNT0;A0A2U9PHF4
179	I7GBQ8	I7GBQ8;A0R059;A0A2U9PTM0
180	A0A653FAJ8	A0A653FAJ8;A0A2U9PQ50;I7G7X0;A0QW[illegible]
181	I7G8V5	I7G8V5;A0QXB9;A0A653FET8;A0A2U9PQ[illegible]
182	I7GFS6	I7GFS6;A0R4H6
183	I7GBR5	I7GBR5;A0A2U9PTM9;A0R069
184	A0QRX7	A0QRX7;A0A2U9PKK3;I7G562
185	A0A2U9Q0A4	A0A2U9Q0A4;I7GGM6;A0R6W9;A0A653F8
186	A0A2U9PMS1	A0A2U9PMS1

187	I7G7T9	I7G7T9;A0QR89;A0A2U9PNH2
188	A0R2V7	A0R2V7;A0A2U9PW98
189	I7G8G3	I7G8G3;A0R401;A0A2U9PZ44
190	A0QYW3	A0QYW3;I7FN97
191	I7GFY8	I7GFY8;A0R4Z0;A0A653FNB8;A0A2U9PYC
192	A0A2U9PI34	A0A2U9PI34;Q2M5K4;I7G1T1;A0A653FNF
193	I7GA06	I7GA06;A0R576;A0A2U9PYK6
194	I7G4P8	I7G4P8;A0QRC5;A0A2U9PK59
195	A0R5R7	A0R5R7;A0A653F8N7;A0A2U9PZF9
196	I7G509	I7G509;A0R0F7;A0A653FB51;A0A2U9PU2
197	I7FKY7	I7FKY7;A0R409;A0A2U9PXJ7
198	I7FZN9	I7FZN9;A0QUM7;A0A653FIN7;A0A2U9PNE
199	A0QSD5	A0QSD5;A0A2U9PL20
200	A0A2U9PY83	A0A2U9PY83
201	I7FLQ8	I7FLQ8;A0QXF8;A0A653FF84;A0A2U9PR0
202	A0A2U9PV26	A0A2U9PV26;A0R1H2;I7GCU5
203	P41403	P41403;I7GFK4;A0R5N8;A0A2U9PZ43
204	A0A653FPF2	A0A653FPF2
205	I7FWG9	I7FWG9;A0QPQ3
206	I7GCR7	I7GCR7;A0QZJ4;A0A2U9PTA1
207	I7G1Y7	I7G1Y7;A0QXY1;A0A653FMY9;A0A2U9PRN
208	A0A2U9PUF8	A0A2U9PUF8
209	A0R2X7	A0R2X7
210	I7FI02	I7FI02
211	A0A653FPX8	A0A653FPX8;L8FLZ2;I7FCD9;A0QNK1
212	A0A653FLV3	A0A653FLV3;I7GGI8;A0R6F7
213	I7G7S7	I7G7S7;A0QWE1;A0A2U9PQ29
214	A0A2U9PX01	A0A2U9PX01
215	A0A2U9PH71	A0A2U9PH71
246	A0A2U9PZ15	A0A2U9PZ15;A0R5L6;I7FMH0
217	I7GF02	I7GF02;A0R4K9;A0A2U9PY31
218	I7G751	I7G751;A0R2V4;A0A653FE88;A0A2U9PWF
219	I7GFT0	I7GFT0;A0R4I6;A0A2U9PXZ3
220	A0A653FME3	A0A653FME3;I7G9H1;A0R4W3
221	I7F7A7	I7F7A7;A0QR61
222	A0A2U9PV64	A0A2U9PV64;I7G601;A0R1J4
223	I7GD17	I7GD17;A0QZZ0;A0A2U9PTL7

Majority protein IDs	Protein names
A0A2U9PZ50;I7GAD7;A0R5M3;A0A	Methanol:N,N-dimethyl-4-nitrosoaniline oxidoreductase
A0A2U9PVX0;I7GET1;A0R2H8;A0A(	L-glutamate gamma-semialdehyde dehydrogenase (EC
A0QQU5;A0A2U9PJL5	Chaperonin GroEL 2 (EC 5.6.1.7) (60 kDa chaperonin 2
I7G686;A0QT50;A0A2U9PLU0	Periplasmic binding protein/LacI transcriptional regulat
A0QSS3;A0A2U9PLG7	Co-chaperonin GroES (10 kDa chaperonin) (Chaperonir
A0A2U9PQI5;A0QWW2;Q9LBS3	Glyceraldehyde-3-phosphate dehydrogenase (EC 1.2.1
A0A2U9PJ55;A0QQC8	Chaperone protein DnaK (HSP70) (Heat shock 70 kDa
A0R3M4;A0A2U9PX87	Succinate--CoA ligase [ADP-forming] subunit beta (EC
A0A2U9PYX5;A0A653FAC7;A0R5G1	Acetyl-coenzyme A synthetase (AcCoA synthetase) (Ac
I7G5V6;A0A2U9PLC0;A0QSN7	Probable aldehyde dehydrogenase (EC 1.2.1.3)
Q9LBQ3;I7F995;A0QT51;A0A653FK	Ribulokinase (EC 2.7.1.16)
A0A2U9PKX4;A0QS98	Elongation factor Tu (EF-Tu) (EC 3.6.5.3)
A4ZHR8;A0A2U9PM45	Adenosylhomocysteinase (EC 3.13.2.1) (S-adenosyl-L-l
I7FRX4;A0R2Y1;A0A653FET9;A0A2	Acetyl-CoA acetyltransferase (EC 2.3.1.-)
A0A2U9PNL7;I7FZT6;A0QUV7	Electron transfer flavoprotein, alpha subunit
Q9ZHC5;Q4PLR8;A0A2U9PNN7	DNA-binding protein HupB (HupB) (EC 1.16.3.1) (Cold
I7GAH5;A0QWY0;A0A653FA46;A0A	Transketolase (EC 2.2.1.1)
I7G2C2;A0QNF6;A0A2U9PH26	Peptidyl-prolyl cis-trans isomerase (PPIase) (EC 5.2.1.8
I7GDR8;A0R2U8;A0A653FG35;A0A2	Fumarate hydratase class II (Fumarase C) (EC 4.2.1.2)
A0R0B3;A0A2U9PTY7	Meromycolate extension acyl carrier protein (ACP)
A0R3B8;A0A2U9PWX2	Enolase (EC 4.2.1.11) (2-phospho-D-glycerate hydro-ly
I7G5C1;A0QS63;A0A2U9PL09	Large ribosomal subunit protein bL12
A0QVB9;A0A2U9PNW9	Elongation factor Ts (EF-Ts)
A0QT96;I7G4T8;A0A2U9PLW1	aldehyde dehydrogenase (NAD(+)) (EC 1.2.1.3)
I7G6A9;A0QUV6;A0A2U9PNT6	Electron transfer flavoprotein subunit beta (Electron tra
A0QSZ3;I7FYJ4;A0A2U9PLP3	Isocitrate dehydrogenase [NADP] (EC 1.1.1.42) (Oxalo
A0R200;A0A2U9PVD8	ATP synthase subunit beta (EC 7.1.2.2) (ATP synthase
I7GGP4;A0R761;A0A653FA40;A0A2	3-hydroxyacyl-CoA dehydrogenase (EC 1.1.1.35)
I7G6G9;A0QTE1;A0A2U9PMC8	Biotin-dependent acyl-coenzyme A carboxylase alpha3
Q9KGW0;A0R5E1;A0A2U9Q0I8	Probable cold shock protein A
Q9RHG2;A0QT53;A0A2U9PM48	L-arabinose isomerase (EC 5.3.1.4)
A0R006;A0A2U9PTH0	Cell wall synthesis protein Wag31 (Antigen 84)
A0QWS8;A0A2U9PQE2	Integration host factor (Mycobacterial integration host
A0QYY6;A0A2U9PSM7;U5U3X6	Small ribosomal subunit protein bS1 (30S ribosomal pr
A0R202;A0A2U9PVH1	ATP synthase subunit alpha (EC 7.1.2.2) (ATP synthase
I7G2U5;A0QR68;A0A2U9PNC7	Methyltransferase type 11 (EC 2.1.1.64)
P41193;A0QS97;A0A2U9PKX8	Small ribosomal subunit protein uS7 (30S ribosomal pr
A0QSD4;A0A2U9PL18	Large ribosomal subunit protein uL2 (50S ribosomal pr
A0R757;A0A2U9Q0H5	D-erythrulose-4-phosphate isomerase 2 (EC 5.3.1.34)
A0R758;A0A653F837;A0A2U9Q0G2	L-erythrulose 1-kinase (EC 2.7.1.209)
I7FSI8;A0R3M3;A0A2U9PX88	Succinate--CoA ligase [ADP-forming] subunit alpha (EC
I7FFV0;A0QZ83;A0A2U9PSY2	Heat shock protein hspX
A0QVQ5;A0A2U9PPG3	Polyribonucleotide nucleotidyltransferase (EC 2.7.7.8) (
A0A2U9PKY1;A0QSA8;I7G3X8	ornithine aminotransferase (EC 2.6.1.13) (Ornithine--o
I7FBH2;A0QV51;A0A2U9PNR0	methylmalonate-semialdehyde dehydrogenase (CoA ac

I7G9R3;A0QXZ5;A0A2U9PRU9	Thiol peroxidase (Tpx) (EC 1.11.1.24) (Peroxiredoxin t
I7FEK8;A0QY23;A0A653FMV3;A0A2	Fructose-bisphosphate aldolase class 1 (EC 4.1.2.13) (
I7GF12;A0R2V6;A0A653FE97;A0A2	Acyl-acyl-carrier protein desaturase DesA2 (EC 1.14.19
Q9AGJ6;A0QP32;A0A2U9PHP3	Phosphoenolpyruvate carboxykinase [GTP] (PEP carbo:
Q939T2;A0R079;A0A2U9PTN9	Glutamine synthetase (EC 6.3.1.2)
A0QSG4;A0A2U9PL58	Large ribosomal subunit protein uL6 (50S ribosomal pr
A0QUX8;A0A2U9PNR1	Ketol-acid reductoisomerase (NADP(+)) (KARI) (EC 1.1
I7FGD9;A0QSG5;A0A2U9PL48	Large ribosomal subunit protein uL18
I7FMR4;A0QYD5;A0A2U9PS55	Luciferase-like oxidoreductase (EC 1.5.99.11)
A0R609;A0A2U9PZI9;P0C580	Catalase-peroxidase 1 (CP 1) (EC 1.11.1.21) (Peroxidas
I7G4N0;A0QT42;A0A2U9PLT3	ABC transporter sugar-binding protein (EC 3.6.3.17)
I7G8I0;A0R417;A0A2U9PXH6	Citrate synthase
A0QSL8;A0A2U9PLK5	DNA-directed RNA polymerase subunit alpha (RNAP su
I7FL78;A0R4B3;A0A653FC62;A0A2U	Acyl-acyl-carrier protein desaturase DesA1 (EC 1.14.19
I7FGZ1;A0R0A1;A0A653FCX4;A0A2	Glyoxalase/bleomycin resistance protein/dioxygenase
A0QX20;A0A653FBS0;A0A2U9PQM	Aconitate hydratase A (ACN) (Aconitase) (EC 4.2.1.3) (
I7G6M8;A0QV52;A0A2U9PNU5	Aminotransferase class-III (EC 2.6.1.-)
A0QWW3;A0A2U9PQJ9	Phosphoglycerate kinase (EC 2.7.2.3)
A0R2X1;A0A2U9PWK5	Transcription elongation factor GreA (Transcript cleava
I7FI17;A0R1B3;A0A653FD79;A0A2U	Aminopeptidase N (EC 3.4.11.2) (Alanine aminopeptida
Q7WYN1;A0QWT3;A0A2U9PQG3	S-adenosylmethionine synthase (AdoMet synthase) (E(
A0QS62;A0A2U9PKW5	Large ribosomal subunit protein uL10 (50S ribosomal p
A0QSP9;A0A2U9PLP0	Small ribosomal subunit protein uS9 (30S ribosomal pr
A0QS46;A0A2U9PKS3	Large ribosomal subunit protein uL1 (50S ribosomal pr
Q9RP36;A0QYG2;A0A2U9PS38	Putative signal transduction protein GarA
A0QWX9;A0A2U9PQJ2	Transaldolase (EC 2.2.1.2)
A0QSD1;A0A2U9PL03	Large ribosomal subunit protein uL3 (50S ribosomal pr
I7G4E1;A0QQX6;A0A2U9PJP3	Isocitrate lyase (EC 4.1.3.1)
A0QSL7;A0A2U9PL94	Small ribosomal subunit protein uS4 (30S ribosomal pr
I7G7Z8;A0R3N8;A0A653F9R4;A0A2	Succinate semialdehyde dehydrogenase (EC 1.2.1.16)
A0A2U9PUU3;I7FQK1;A0R1A7;A0A	Ribose-5-phosphate isomerase B (EC 5.3.1.6) (Phosph
A0R1V9;Q57529;A0A2U9PVH6	Alkyl hydroperoxide reductase C (EC 1.11.1.28) (Peroxi
P53649;A0R652;A0A653F936;A0A2	Superoxide dismutase [Mn] (EC 1.15.1.1)
A0A2U9PSB5;A0QYG3;Q9RP37	Glycine cleavage system H protein
A0QV10;A0A653FE78;A0A2U9PNM	Aldo-keto reductase MSMEG_2408/MSMEI_2347 (EC 1.
A0QSL5;A0A2U9PL95	Small ribosomal subunit protein uS13 (30S ribosomal p
I7FEX4;A0QYF7;A0A653FFH9;A0A2	Glycine dehydrogenase (decarboxylating) (EC 1.4.4.2)
A0QP20;I7FVV1;A0A2U9PHQ2	Haemophore haem-binding domain-containing protein
A0R7F9;A0A2U9Q0X2	Small ribosomal subunit protein bS6 (30S ribosomal pr
O06114;A0QSD2;A0A2U9PL07	Large ribosomal subunit protein uL4 (50S ribosomal pr
A0QXX7;A0A653FN71;A0A2U9PRT(	Catalase-peroxidase 2 (CP 2) (EC 1.11.1.21) (Peroxidas
A0QV37;A0A653FFJ7;A0A2U9PNU6	Small ribosomal subunit protein bS16 (30S ribosomal p
A0QSG0;A0A2U9PL60	Large ribosomal subunit protein uL24 (50S ribosomal p
A0A2U9PHC6;A0QNM4;I7F4L2;A0A	Peroxisomal hydratase-dehydrogenase-epimerase
Q7WYH3;I7GEQ6;A0R2E3;A0A2U9I	Calmodulin-like protein
I7G1P3;A0QPE7;A0A2U9PHZ5	3-oxoacyl-acyl-carrier protein reductase FabG4 (EC 1.1
A0QYU6;A0A2U9PSH6	Large ribosomal subunit protein bL20 (50S ribosomal p

A0QSD3;A0A2U9PL26	Large ribosomal subunit protein uL23 (50S ribosomal p
I7F6Q4;A0QQN5;A0A2U9PJI1	Amidase, hydantoinase/carbamoylase (EC 3.5.1.87)
A0QSG1;A0A2U9PLA3	Large ribosomal subunit protein uL5 (50S ribosomal pr
I7FI06;A0R198;A0A2U9PW74	ATP-dependent Clp protease proteolytic subunit (EC 3.
A0QSP8;A0A653FEZ4;A0A2U9PLC7	Large ribosomal subunit protein uL13 (50S ribosomal p
Q9RHG3;Q9LBQ2;I7G4P3;A0QT52;.	L-ribulose-5-phosphate 4-epimerase (EC 5.1.3.4)
P00215;I7G6W3;A0R2I1;A0A2U9PV	Ferredoxin
I7G9Y1;A0QY79;A0A2U9PRW3	Bacterioferritin (EC 1.16.3.1)
I7GD73;A0R072;A0A653FAW6;A0A	Dihydrolipoamide acetyltransferase component of pyru
A0QYF5;I7FMS9;A0A2U9PS74	Malate synthase G (EC 2.3.3.9)
A0A2U9PVC8;A0R1Y7	Probable acetyl-CoA acetyltransferase (EC 2.3.1.9) (Ac
I7G8G0;A0A2U9PQK2;A0QWY3;A0/	Quinone reductase qor (NADPH:quinone reductase) (Z
A0QSX4;I7G631;A0A2U9PLX8	Nitroreductase family protein
A0QSE0;A0A2U9PL81	Small ribosomal subunit protein uS17 (30S ribosomal p
A0QRN3;A0A653FG66;A0A2U9PKG4	Urocanate hydratase (Urocanase) (EC 4.2.1.49) (Imida
A0QSS4;A0A653FFK6;A0A2U9PLK8	Chaperonin GroEL 1 (EC 5.6.1.7) (60 kDa chaperonin 1
O06115;A0QSD6;A0A2U9PL12	Large ribosomal subunit protein uL22 (50S ribosomal p
A0QVB8;A0A2U9PNW3	Small ribosomal subunit protein uS2 (30S ribosomal pr
A0QSG3;A0A2U9PL37	Small ribosomal subunit protein uS8 (30S ribosomal pr
A0R1Z9;A0A2U9PVE4	ATP synthase epsilon chain (ATP synthase F1 sector ep
I7G4Z3;A0QRN7;A0A653FGE3;A0A	Histidine ammonia-lyase (EC 4.3.1.3)
A0QVQ3;A0A2U9PPF0	Small ribosomal subunit protein uS15 (30S ribosomal p
A0QS45;A0A2U9PKR6	Large ribosomal subunit protein uL11 (50S ribosomal p
A0A2U9PLF2	Large ribosomal subunit protein uL15
A0QVE0;A0A2U9PP60	Ribosome-recycling factor (RRF) (Ribosome-releasing f
I7GFG2;A0R5B0;A0A653FNR6;A0A2	Anti-sigma factor antagonist
A0R6E3;A0A2U9PZT5	Uncharacterized protein MSMEG_6518/MSMEI_6344
A0R170;I7GCM1;A0A653FBM7;A0A	Alpha oxoglutarate ferredoxin oxidoreductase, beta sul
I7F8U3;A0QSN8;A0A653FF04;A0A2	Protein glcG
A0R199;A0A653FBE8;A0A2U9PUP9	Trigger factor (TF) (EC 5.2.1.8) (PPIase)
I7GBX4;A0R0C7;A0A2U9PU03	Zinc-dependent alcohol dehydrogenase AdhE2 (EC 1.1.
A0R7F6;A0A653F9J9;A0A2U9Q132	Large ribosomal subunit protein bL9 (50S ribosomal pr
A0R0B2;A0A2U9PTZ0	Malonyl CoA-acyl carrier protein transacylase (MCT) (E
A0R7F7;A0A2U9Q139	Small ribosomal subunit protein bS18B (30S ribosomal
I7FPG4;A0R7J6;A0A2U9Q112	Single-stranded nucleic acid binding R3H
A0R5Z0;A0A2U9PZH2	Bacterial proteasome activator
A0QSH8;A0A2U9PLG2	Adenylate kinase (AK) (EC 2.7.4.3) (ATP-AMP transpho
A0R0B0;A0A2U9PTU4	Pyruvate dehydrogenase E1 component (PDH E1 comp
I7GFQ8;A0R623;A0A2U9PZI7	Esterase
A0QSD0;A0A2U9PL74	Small ribosomal subunit protein uS10 (30S ribosomal p
A0A2U9PS95;I7FMU4;A0QYH7	Mannose-binding lectin
I7F8Q0;A0QSJ2;A0A653FFU6;A0A2	methylmalonate-semialdehyde dehydrogenase (CoA ac
I7G4Y4;A0QTE3;A0A2U9PM55	Sulfurtransferase
A0A2U9PLF7;A0QSL9;A0A653FF30	Large ribosomal subunit protein bL17
Q8RME2;I7F8C1;A4ZHU4;A0A653FI	Elongation factor G (EF-G)
I7GDX1;A0R197;A0A2U9PUV0	ATP-dependent Clp protease proteolytic subunit (EC 3.
A0QSD7;A0A2U9PLC2	Small ribosomal subunit protein uS3 (30S ribosomal pr

Q9AFI5;A0A2U9Q0W2	Single-stranded DNA-binding protein (SSB)
A0R597;A0A653FM64;A0A2U9PYR7	Inorganic pyrophosphatase (EC 3.6.1.1) (Pyrophosphat
A0A2U9PQP6;I7G8L7;A0QX35	Methylmalonyl-CoA mutase small subunit (EC 5.4.99.2)
I7F4Z3;A0A653FNY1;A0QNZ3;A0A2	Short-chain dehydrogenase/reductase SDR (EC 1.1.1.-)
I7F603;A0QPY2;A0A2U9PIQ9	Probable cold shock protein A
I7FEW5;A0QYE7;A0A2U9PS96	6-phosphogluconate dehydrogenase, decarboxylating (
P0C558;A0R692;A0A2U9PZP1	DNA protection during starvation protein (EC 1.16.-.-)
I7GGF4;A0A2U9PZT1;A0R656	Nucleoid-associated protein EspR
A0QSD8;A0A2U9PL83	Large ribosomal subunit protein uL16 (50S ribosomal p
I7FX95;A0QQW8;A0A653FDR7;A0A	Dihydrolipoyl dehydrogenase (EC 1.8.1.4)
A0QYU8;I7GC56	Translation initiation factor IF-3
A0QSD9;A0A2U9PL45	Large ribosomal subunit protein uL29 (50S ribosomal p
I7FJZ3;A0QVR8;A0A2U9PPI6	Dienelactone hydrolase (EC 3.1.1.45)
I7FVQ8;A0R760;A0A2U9Q0J3	Xylose isomerase-like TIM barrel
A0R6B7;A0A653FLD7;A0A2U9PZS4;	GAF domain protein, putative
I7G6W0;A0QTR2;A0A2U9PMG7	ATP-binding protein
I7G4K7;A0QT22;A0A653FJ55;A0A2	Primosomal protein
A0R151;A0A2U9PUJ1	Large ribosomal subunit protein bL21 (50S ribosomal p
I7GFH6;A0R5H1;A0A2U9PYU9	CRP-like cAMP-activated global transcriptional regulato
O30973;I7FW12;A0R7I9;A0A653F8	Thioredoxin reductase (TRXR) (EC 1.8.1.9)
I7G436;A0QSG8;A0A653FF82	Large ribosomal subunit protein uL15
P96803;A0R3N9;A0A653F9Q7;A0A2	Glucose-6-phosphate isomerase (GPI) (EC 5.3.1.9) (Ph
P0C563;A0QS96;A0A2U9PKZ1	Small ribosomal subunit protein uS12 (30S ribosomal p
I7G2H6;A0QQQ1;A0A2U9PJH5	Superoxide dismutase [Cu-Zn] (EC 1.15.1.1)
A0R102;A0A2U9PUD4	Small ribosomal subunit protein bS20 (30S ribosomal p
A0QQC1;I7F6E6;A0A2U9PJC2	Monooxygenase
A0QXD8;A0A2U9PQY6	Erythritol/L-threitol dehydrogenase (EC 1.1.1.-)
I7GAH8;A0R5S2;A0A2U9PZB0	Transcription elongation factor
A0R617;I7FMV0;A0A653F966;A0A2	Polyketide synthase
I7GBA7;A0QXW3;A0A2U9PRI1	Two-component system response regulator, LuxR fami
A0A2U9Q0F0	Amine oxidase domain-containing protein
A0QY95;A0A2U9PTD1;I7G354	Antigen 85-C (EC 2.3.1.-)
I7F8K7;A0QSF2;A0A653FFZ1;A0A2	Coenzyme PQQ synthesis C (EC 1.3.3.11)
I7FRM7;A0R2H7;A0A2U9PVX6	proline dehydrogenase (EC 1.5.5.2)
A0QSG7	Large ribosomal subunit protein uL30 (50S ribosomal p
I7GEW9;A0R4G4;A0A653FC78;A0A	Phosphoribosylformylglycinamidine synthase subunit P
A0QXS8;I7FE72	MOSC domain protein
A0R2K9	Uncharacterized protein
I7F4S5;A0QNT0;A0A2U9PHF4	NAD(P) transhydrogenase subunit alpha part 1 (EC 7.1
I7GBQ8;A0R059;A0A2U9PTM0	Carbohydrate kinase CbhK (EC 2.7.1.20)
A0A653FAJ8;A0A2U9PQ50;I7G7X0;	Putative sugar epimerase YhfK
I7G8V5;A0QXB9;A0A653FET8;A0A2	Transcriptional regulatory protein PdtaR
I7GFS6;A0R4H6	Glutathione peroxidase
I7GBR5;A0A2U9PTM9;A0R069	Probable cytosol aminopeptidase (EC 3.4.11.1) (Leucin
A0QRX7;A0A2U9PKK3;I7G562	HNH nuclease, putative
A0A2U9Q0A4;I7GGM6;A0R6W9;A0	Cache domain-containing protein
A0A2U9PMS1	Phosphoenolpyruvate synthase (EC 2.7.9.2) (Pyruvate,

I7G7T9;A0QR89;A0A2U9PNH2	Geranylgeranyl reductase
A0R2V7;A0A2U9PW98	Serine hydroxymethyltransferase (SHMT) (Serine meth
I7G8G3;A0R401;A0A2U9PZ44	Histidinol dehydrogenase
A0QYW3;I7FN97	Luciferase-like domain-containing protein
I7GFY8;A0R4Z0;A0A653FNB8;A0A2	Coa-transferase (Alpha subunit) (EC 2.8.3.12)
A0A2U9PI34;Q2M5K4;I7G1T1;A0A6	MmpL protein
I7GA06;A0R576;A0A2U9PYK6	Nucleoid-associated protein Lsr2
I7G4P8;A0QRC5;A0A2U9PK59	Glyoxalase/bleomycin resistance protein/dioxygenase
A0R5R7;A0A653F8N7;A0A2U9PZF9	Putative aminotransferase MSMEG_6286/MSMEI_6121
I7G509;A0R0F7;A0A653FB51;A0A2	Transcriptional regulator, CdaR family
I7FKY7;A0R409;A0A2U9PXJ7	Peptidyl-prolyl cis-trans isomerase (EC 5.2.1.8)
I7FZN9;A0QUM7;A0A653FIN7;A0A2	Nickel-dependent hydrogenase large subunit (EC 1.12.
A0QSD5;A0A2U9PL20	Small ribosomal subunit protein uS19 (30S ribosomal p
A0A2U9PY83	O-antigen polymerase, putative
I7FLQ8;A0QXF8;A0A653FF84;A0A2	Isocitrate dehydrogenase, NAD-dependent (EC 1.1.1.-)
A0A2U9PV26;A0R1H2;I7GCU5	DUF3618 domain-containing protein
P41403;I7GFK4;A0R5N8;A0A2U9PZ	Aspartokinase (EC 2.7.2.4) (Aspartate kinase) (ASK)
A0A653FPF2	Uncharacterized protein
I7FWG9;A0QPQ3	Uncharacterized protein
I7GCR7;A0QZJ4;A0A2U9PTA1	L-carnitine dehydratase/bile acid-inducible protein F (E
I7G1Y7;A0QXY1;A0A653FMY9;A0A2	Acyl-AMP synthetase
A0A2U9PUF8	Thioester domain-containing protein
A0R2X7	Uncharacterized protein
I7FI02	Uncharacterized protein
A0A653FPX8;L8FLZ2;I7FCD9;A0QN	ESX-1 secretion-associated protein EspK
A0A653FLV3;I7GGI8;A0R6F7	HTH-type transcriptional regulator MtrR
I7G7S7;A0QWE1;A0A2U9PQ29	GntR family transcriptional regulator
A0A2U9PX01	Peptidase S1 and S6, chymotrypsin/Hap
A0A2U9PH71	IS1096, tnpA protein
A0A2U9PZ15;A0R5L6;I7FMH0	Thiopurine S-methyltransferase (Tpmt) superfamily pro
I7GF02;A0R4K9;A0A2U9PY31	Histidine triad (HIT) protein
I7G751;A0R2V4;A0A653FE88;A0A2	NAD(P)H nitroreductase acg
I7GFT0;A0R4I6;A0A2U9PXZ3	Adenylosuccinate lyase (ASL) (EC 4.3.2.2) (Adenylosuc
A0A653FME3;I7G9H1;A0R4W3	Uncharacterized protein
I7F7A7;A0QR61	Methyltransferase type 12
A0A2U9PV64;I7G601;A0R1J4	Enoyl-CoA hydratase/isomerase family protein
I7GD17;A0QZZ0;A0A2U9PTL7	Phospholipid-binding protein, PBP family

**Gene Names**	**Fasta headers**
D806_061380	tr|A0A2U9PZ50|A0A2U9PZ50_MYCSE Methanol:N,N-dimethyl-4-nitros
D806_050100	tr|A0A2U9PVX0|A0A2U9PVX0_MYCSE L-glutamate gamma-semialdeh
groEL2 groL2 MSMEG_0880 I	sp|A0QQU5|CH602_MYCS2 Chaperonin GroEL 2 OS=Mycolicibacteriur
MSMEI_1672	tr|I7G686|I7G686_MYCS2 Periplasmic binding protein/LacI transcripti
groES groS MSMEG_1582 MS	sp|A0QSS3|CH10_MYCS2 Co-chaperonin GroES OS=Mycolicibacterium
D806_030480	tr|A0A2U9PQI5|A0A2U9PQI5_MYCSE Glyceraldehyde-3-phosphate de
dnaK D806_007820	tr|A0A2U9PJ55|A0A2U9PJ55_MYCSE Chaperone protein DnaK OS=My
sucC MSMEG_5525 MSMEI_5	sp|A0R3M4|SUCC_MYCS2 Succinate--CoA ligase [ADP-forming] subur
acsA D806_060530	tr|A0A2U9PYX5|A0A2U9PYX5_MYCSE Acetyl-coenzyme A synthetase
MSMEI_1506	tr|I7G5V6|I7G5V6_MYCS2 Probable aldehyde dehydrogenase OS=My
araB	sp|Q9LBQ3|ARAB_MYCSM Ribulokinase OS=Mycolicibacterium smegn
tuf D806_014190	tr|A0A2U9PKX4|A0A2U9PKX4_MYCSE Elongation factor Tu OS=Mycoli
ahcY MSMEG_1843 MSMEI_1	sp|A4ZHR8|SAHH_MYCS2 Adenosylhomocysteinase OS=Mycolicibacte
fadA3 MSMEI_5134	tr|I7FRX4|I7FRX4_MYCS2 Acetyl-CoA acetyltransferase OS=Mycolicib
D806_023250	tr|A0A2U9PNL7|A0A2U9PNL7_MYCSE Electron transfer flavoprotein, a
hup cipMa hlp MSMEG_2389	sp|Q9ZHC5|DBH_MYCS2 DNA-binding protein HupB OS=Mycolicibacte
tkt MSMEI_3025	tr|I7GAH5|I7GAH5_MYCS2 Transketolase OS=Mycolicibacterium smeg
ppiA MSMEI_0026	tr|I7G2C2|I7G2C2_MYCS2 Peptidyl-prolyl cis-trans isomerase OS=My
fumC MSMEI_5102	tr|I7GDR8|I7GDR8_MYCS2 Fumarate hydratase class II OS=Mycolicib
acpM MSMEG_4326 MSMEI_	sp|A0R0B3|ACPM_MYCS2 Meromycolate extension acyl carrier proteir
eno MSMEG_5415 MSMEI_52	sp|A0R3B8|ENO_MYCS2 Enolase OS=Mycolicibacterium smegmatis (s
rplL MSMEI_1326	tr|I7G5C1|I7G5C1_MYCS2 Large ribosomal subunit protein bL12 OS=
tsf MSMEG_2520 MSMEI_24	sp|A0QVB9|EFTS_MYCS2 Elongation factor Ts OS=Mycolicibacterium
MSMEG_1762	tr|A0QT96|A0QT96_MYCS2 aldehyde dehydrogenase (NAD(+)) OS=M
etfB MSMEI_2291	tr|I7G6A9|I7G6A9_MYCS2 Electron transfer flavoprotein subunit beta
MSMEG_1654	tr|A0QSZ3|A0QSZ3_MYCS2 Isocitrate dehydrogenase [NADP] OS=My
atpD MSMEG_4936 MSMEI_4	sp|A0R200|ATPB_MYCS2 ATP synthase subunit beta OS=Mycolicibact
MSMEI_6608	tr|I7GGP4|I7GGP4_MYCS2 3-hydroxyacyl-CoA dehydrogenase OS=My
accA3 MSMEI_1762	tr|I7G6G9|I7G6G9_MYCS2 Biotin-dependent acyl-coenzyme A carbox
cspA	tr|Q9KGW0|Q9KGW0_MYCSM Probable cold shock protein A OS=Myc
araA	sp|Q9RHG2|ARAA_MYCSM L-arabinose isomerase OS=Mycolicibacteri
wag31 ag84 MSMEG_4217 M	sp|A0R006|WAG31_MYCS2 Cell wall synthesis protein Wag31 OS=My
mIHF msihf MSMEG_3050 M	sp|A0QWS8|IHF_MYCS2 Integration host factor OS=Mycolicibacteriun
rpsA MSMEG_3833 MSMEI_3	sp|A0QYY6|RS1_MYCS2 Small ribosomal subunit protein bS1 OS=Myc
atpA MSMEG_4938 MSMEI_4	sp|A0R202|ATPA_MYCS2 ATP synthase subunit alpha OS=Mycolicibac
MSMEI_0976 MSMEI_2227	tr|I7G2U5|I7G2U5_MYCS2 Methyltransferase type 11 OS=Mycolicibac
rpsG	sp|P41193|RS7_MYCSM Small ribosomal subunit protein uS7 OS=Myc
rplB MSMEG_1439 MSMEI_1	sp|A0QSD4|RL2_MYCS2 Large ribosomal subunit protein uL2 OS=Myc
derI2 MSMEG_6787 MSMEI_	sp|A0R757|DERI2_MYCS2 D-erythrulose-4-phosphate isomerase 2 OS
lerK MSMEG_6788 MSMEI_6	sp|A0R758|LERK_MYCS2 L-erythrulose 1-kinase OS=Mycolicibacteriur
sucD MSMEI_5372	tr|I7FSI8|I7FSI8_MYCS2 Succinate--CoA ligase [ADP-forming] subunit
hspX MSMEI_3842	tr|I7FFV0|I7FFV0_MYCS2 Heat shock protein hspX OS=Mycolicibacter
pnp gpsI MSMEG_2656 MSM	sp|A0QVQ5|PNP_MYCS2 Polyribonucleotide nucleotidyltransferase OS
D806_014290	tr|A0A2U9PKY1|A0A2U9PKY1_MYCSE ornithine aminotransferase OS=
MSMEI_2387	tr|I7FBH2|I7FBH2_MYCS2 methylmalonate-semialdehyde dehydrogen

tpx MSMEI_3398	tr|I7G9R3|I7G9R3_MYCS2 Thiol peroxidase OS=Mycolicibacterium sm
MSMEI_3427	tr|I7FEK8|I7FEK8_MYCS2 Fructose-bisphosphate aldolase class 1 OS=
desA2 MSMEI_5110	tr|I7GF12|I7GF12_MYCS2 Acyl-acyl-carrier protein desaturase DesA2
pckG pck	sp|Q9AGJ6|PCKG_MYCSM Phosphoenolpyruvate carboxykinase [GTP]
glnA1	tr|Q939T2|Q939T2_MYCSM Glutamine synthetase OS=Mycolicibacteri
rplF MSMEG_1470 MSMEI_14	sp|A0QSG4|RL6_MYCS2 Large ribosomal subunit protein uL6 OS=Myc
ilvC MSMEG_2374 MSMEI_23	sp|A0QUX8|ILVC_MYCS2 Ketol-acid reductoisomerase (NADP(+)) OS=
rplR MSMEI_1435	tr|I7FGD9|I7FGD9_MYCS2 Large ribosomal subunit protein uL18 OS=
MSMEI_3535	tr|I7FMR4|I7FMR4_MYCS2 Luciferase-like oxidoreductase OS=Mycolic
katG1 MSMEG_6384 MSMEI_	sp|A0R609|KATG1_MYCS2 Catalase-peroxidase 1 OS=Mycolicbacteriu
MSMEI_1664	tr|I7G4N0|I7G4N0_MYCS2 ABC transporter sugar-binding protein OS=
gltA2 MSMEI_5522	tr|I7G8I0|I7G8I0_MYCS2 Citrate synthase OS=Mycolicibacterium sme
rpoA MSMEG_1524 MSMEI_1	sp|A0QSL8|RPOA_MYCS2 DNA-directed RNA polymerase subunit alph
des MSMEI_5619	tr|I7FL78|I7FL78_MYCS2 Acyl-acyl-carrier protein desaturase DesA1 C
MSMEI_4213	tr|I7FGZ1|I7FGZ1_MYCS2 Glyoxalase/bleomycin resistance protein/di
acnA acn MSMEG_3143 MSM	sp|A0QX20|ACNA_MYCS2 Aconitate hydratase A OS=Mycolicibacteriu
MSMEI_2388	tr|I7G6M8|I7G6M8_MYCS2 Aminotransferase class-III OS=Mycolicibac
pgk MSMEG_3085 MSMEI_30	sp|A0QWW3|PGK_MYCS2 Phosphoglycerate kinase OS=Mycolicibacte
greA MSMEG_5263 MSMEI_5	sp|A0R2X1|GREA_MYCS2 Transcription elongation factor GreA OS=My
MSMEI_4573	tr|I7FI17|I7FI17_MYCS2 Aminopeptidase N OS=Mycolicibacterium sm
metK	sp|Q7WYN1|METK_MYCSM S-adenosylmethionine synthase OS=Mycc
rplJ MSMEG_1364 MSMEI_13	sp|A0QS62|RL10_MYCS2 Large ribosomal subunit protein uL10 OS=M
rpsI MSMEG_1557 MSMEI_15	sp|A0QSP9|RS9_MYCS2 Small ribosomal subunit protein uS9 OS=Myc
rplA MSMEG_1347 MSMEI_13	sp|A0QS46|RL1_MYCS2 Large ribosomal subunit protein uL1 OS=Myc
garA	tr|Q9RP36|Q9RP36_MYCSM Putative signal transduction protein GarA
tal MSMEG_3102 MSMEI_302	sp|A0QWX9|TAL_MYCS2 Transaldolase OS=Mycolicibacterium smegm
rplC MSMEG_1436 MSMEI_14	sp|A0QSD1|RL3_MYCS2 Large ribosomal subunit protein uL3 OS=Myc
icl MSMEI_0889	tr|I7G4E1|I7G4E1_MYCS2 Isocitrate lyase OS=Mycolicibacterium sme
rpsD MSMEG_1523 MSMEI_1	sp|A0QSL7|RS4_MYCS2 Small ribosomal subunit protein uS4 OS=Myc
MSMEI_5386	tr|I7G7Z8|I7G7Z8_MYCS2 Succinate semialdehyde dehydrogenase OS
D806_045630	tr|A0A2U9PUU3|A0A2U9PUU3_MYCSE Ribose-5-phosphate isomerase
MSMEG_4891 MSMEI_4766	sp|A0R1V9|AHPC_MYCS2 Alkyl hydroperoxide reductase C OS=Mycoli
sodA sod	sp|P53649|SODM_MYCSM Superoxide dismutase [Mn] OS=Mycoliciba
gcvH D806_036520	tr|A0A2U9PSB5|A0A2U9PSB5_MYCSE Glycine cleavage system H prot
MSMEG_2408 MSMEI_2347	sp|A0QV10|Y2408_MYCS2 Aldo-keto reductase MSMEG_2408/MSMEI_
rpsM MSMEG_1521 MSMEI_1	sp|A0QSL5|RS13_MYCS2 Small ribosomal subunit protein uS13 OS=M
gcvP MSMEI_3557	tr|I7FEX4|I7FEX4_MYCS2 Glycine dehydrogenase (decarboxylating) C
MSMEG_0243	tr|A0QP20|A0QP20_MYCS2 Haemophore haem-binding domain-conta
rpsF MSMEG_6897 MSMEI_6	sp|A0R7F9|RS6_MYCS2 Small ribosomal subunit protein bS6 OS=Myc
rplD	sp|O06114|RL4_MYCSM Large ribosomal subunit protein uL4 OS=Myc
katG2 katH MSMEG_3461 MS	sp|A0QXX7|KATG2_MYCS2 Catalase-peroxidase 2 OS=Mycolicibacteri
rpsP MSMEG_2435 MSMEI_2	sp|A0QV37|RS16_MYCS2 Small ribosomal subunit protein bS16 OS=M
rplX MSMEG_1466 MSMEI_14	sp|A0QSG0|RL24_MYCS2 Large ribosomal subunit protein uL24 OS=M
D806_001010	tr|A0A2U9PHC6|A0A2U9PHC6_MYCSE Peroxisomal hydratase-dehydro
camL	tr|Q7WYH3|Q7WYH3_MYCSM Calmodulin-like protein OS=Mycolicibac
fabG4 MSMEI_0365	tr|I7G1P3|I7G1P3_MYCS2 3-oxoacyl-acyl-carrier protein reductase Fa
rplT MSMEG_3791 MSMEI_37	sp|A0QYU6|RL20_MYCS2 Large ribosomal subunit protein bL20 OS=M

rplW MSMEG_1438 MSMEI_1	sp|A0QSD3|RL23_MYCS2 Large ribosomal subunit protein uL23 OS=M
MSMEI_0800	tr|I7F6Q4|I7F6Q4_MYCS2 Amidase, hydantoinase/carbamoylase OS=
rplE MSMEG_1467 MSMEI_14	sp|A0QSG1|RL5_MYCS2 Large ribosomal subunit protein uL5 OS=Myc
clpP MSMEI_4556	tr|I7FI06|I7FI06_MYCS2 ATP-dependent Clp protease proteolytic subu
rplM MSMEG_1556 MSMEI_1	sp|A0QSP8|RL13_MYCS2 Large ribosomal subunit protein uL13 OS=M
araD	tr|Q9RHG3|Q9RHG3_MYCSM L-ribulose-5-phosphate 4-epimerase (Fr
fdxA	sp|P00215|FER_MYCSM Ferredoxin OS=Mycolicibacterium smegmatis
bfr MSMEI_3483	tr|I7G9Y1|I7G9Y1_MYCS2 Bacterioferritin OS=Mycolicibacterium smeg
sucB MSMEI_4182	tr|I7GD73|I7GD73_MYCS2 Dihydrolipoamide acetyltransferase compo
glcB MSMEG_3640	tr|A0QYF5|A0QYF5_MYCS2 Malate synthase G OS=Mycolicibacterium
D806_048140	tr|A0A2U9PVC8|A0A2U9PVC8_MYCSE Probable acetyl-CoA acetyltrans
MSMEI_3027	tr|I7G8G0|I7G8G0_MYCS2 Quinone reductase qor (NADPH:quinone re
MSMEG_1635	tr|A0QSX4|A0QSX4_MYCS2 Nitroreductase family protein OS=Mycolic
rpsQ MSMEG_1445 MSMEI_1	sp|A0QSE0|RS17_MYCS2 Small ribosomal subunit protein uS17 OS=M
hutU MSMEG_1179 MSMEI_1	sp|A0QRN3|HUTU_MYCS2 Urocanate hydratase OS=Mycolicibacterium
groEL1 groL1 MSMEG_1583 I	sp|A0QSS4|CH601_MYCS2 Chaperonin GroEL 1 OS=Mycolicibacterium
rplV	sp|O06115|RL22_MYCSM Large ribosomal subunit protein uL22 OS=M
rpsB MSMEG_2519 MSMEI_2	sp|A0QVB8|RS2_MYCS2 Small ribosomal subunit protein uS2 OS=Myc
rpsH MSMEG_1469 MSMEI_1	sp|A0QSG3|RS8_MYCS2 Small ribosomal subunit protein uS8 OS=Myc
atpC MSMEG_4935 MSMEI_4	sp|A0R1Z9|ATPE_MYCS2 ATP synthase epsilon chain OS=Mycolicibac
MSMEI_1152	tr|I7G4Z3|I7G4Z3_MYCS2 Histidine ammonia-lyase OS=Mycolicibacte
rpsO MSMEG_2654 MSMEI_2	sp|A0QVQ3|RS15_MYCS2 Small ribosomal subunit protein uS15 OS=M
rplK MSMEG_1346 MSMEI_13	sp|A0QS45|RL11_MYCS2 Large ribosomal subunit protein uL11 OS=M
rplO D806_014900	tr|A0A2U9PLF2|A0A2U9PLF2_MYCSE Large ribosomal subunit protein
frr MSMEG_2541 MSMEI_248	sp|A0QVE0|RRF_MYCS2 Ribosome-recycling factor OS=Mycolicibacter
rsfB MSMEI_5968	tr|I7GFG2|I7GFG2_MYCS2 Anti-sigma factor antagonist OS=Mycolicib
MSMEG_6518 MSMEI_6344	sp|A0R6E3|Y6518_MYCS2 Uncharacterized protein MSMEG_6518/MSM
orB MSMEG_4645	tr|A0R170|A0R170_MYCS2 Alpha oxoglutarate ferredoxin oxidoreduct
MSMEI_1507	tr|I7F8U3|I7F8U3_MYCS2 Protein glcG OS=Mycolicibacterium smegma
tig MSMEG_4674 MSMEI_455	sp|A0R199|TIG_MYCS2 Trigger factor OS=Mycolicibacterium smegma
adhE2 MSMEI_4240	tr|I7GBX4|I7GBX4_MYCS2 Zinc-dependent alcohol dehydrogenase Ad
rplI MSMEG_6894 MSMEI_67	sp|A0R7F6|RL9_MYCS2 Large ribosomal subunit protein bL9 OS=Myc
fabD MSMEG_4325 MSMEI_4	sp|A0R0B2|FABD_MYCS2 Malonyl CoA-acyl carrier protein transacylas
rpsR2 rpsR1 MSMEG_6895 M	sp|A0R7F7|RS182_MYCS2 Small ribosomal subunit protein bS18B OS=
MSMEI_6749	tr|I7FPG4|I7FPG4_MYCS2 Single-stranded nucleic acid binding R3H O
bpa MSMEG_6365 MSMEI_61	sp|A0R5Z0|BPA_MYCS2 Bacterial proteasome activator OS=Mycoliciba
adk MSMEG_1484 MSMEI_14	sp|A0QSH8|KAD_MYCS2 Adenylate kinase OS=Mycolicibacterium sme
aceE MSMEG_4323 MSMEI_4	sp|A0R0B0|ODP1_MYCS2 Pyruvate dehydrogenase E1 component OS
MSMEI_6230	tr|I7GFQ8|I7GFQ8_MYCS2 Esterase OS=Mycolicibacterium smegmatis
rpsJ MSMEG_1435 MSMEI_13	sp|A0QSD0|RS10_MYCS2 Small ribosomal subunit protein uS10 OS=M
D806_036650	tr|A0A2U9PS95|A0A2U9PS95_MYCSE Mannose-binding lectin OS=Myc
mmsA MSMEI_1462	tr|I7F8Q0|I7F8Q0_MYCS2 methylmalonate-semialdehyde dehydrogen
sseA MSMEI_1764	tr|I7G4Y4|I7G4Y4_MYCS2 Sulfurtransferase OS=Mycolicibacterium sm
rplQ D806_015410	tr|A0A2U9PLF7|A0A2U9PLF7_MYCSE Large ribosomal subunit protein
fusA	tr|Q8RME2|Q8RME2_MYCSM Elongation factor G OS=Mycolicibacteriu
clpP MSMEI_4555	tr|I7GDX1|I7GDX1_MYCS2 ATP-dependent Clp protease proteolytic su
rpsC MSMEG_1442 MSMEI_1	sp|A0QSD7|RS3_MYCS2 Small ribosomal subunit protein uS3 OS=Myc

ssb MSMEG_6896 MSMEI_67	sp|Q9AFI5|SSB_MYCS2 Single-stranded DNA-binding protein OS=Myc
ppa MSMEG_6114	tr|A0R597|A0R597_MYCS2 Inorganic pyrophosphatase OS=Mycoliciba
D806_031200	tr|A0A2U9PQP6|A0A2U9PQP6_MYCSE Methylmalonyl-CoA mutase sm
MSMEI_0209	tr|I7F4Z3|I7F4Z3_MYCS2 Short-chain dehydrogenase/reductase SDR
cspA MSMEI_0544	tr|I7F603|I7F603_MYCS2 Probable cold shock protein A OS=Mycolicib
gnd MSMEI_3547	tr|I7FEW5|I7FEW5_MYCS2 6-phosphogluconate dehydrogenase, deca
dps	sp|P0C558|DPS_MYCSM DNA protection during starvation protein OS
MSMEI_6263	tr|I7GGF4|I7GGF4_MYCS2 Nucleoid-associated protein EspR OS=Myc
rplP MSMEG_1443 MSMEI_1	sp|A0QSD8|RL16_MYCS2 Large ribosomal subunit protein uL16 OS=
lpd MSMEI_0882	tr|I7FX95|I7FX95_MYCS2 Dihydrolipoyl dehydrogenase OS=Mycoliciba
infC MSMEG_3793	tr|A0QYU8|A0QYU8_MYCS2 Translation initiation factor IF-3 OS=Myc
rpmC MSMEG_1444 MSMEI_	sp|A0QSD9|RL29_MYCS2 Large ribosomal subunit protein uL29 OS=
MSMEI_2605	tr|I7FJZ3|I7FJZ3_MYCS2 Dienelactone hydrolase OS=Mycolicibacteriu
MSMEI_6607	tr|I7FVQ8|I7FVQ8_MYCS2 Xylose isomerase-like TIM barrel OS=Myco
MSMEG_6492	tr|A0R6B7|A0R6B7_MYCS2 GAF domain protein, putative OS=Mycolic
MSMEI_1892	tr|I7G6W0|I7G6W0_MYCS2 ATP-binding protein OS=Mycolicibacteriu
MSMEI_1644	tr|I7G4K7|I7G4K7_MYCS2 Primosomal protein OS=Mycolicibacterium
rplU MSMEG_4625 MSMEI_4	sp|A0R151|RL21_MYCS2 Large ribosomal subunit protein bL21 OS=M
MSMEI_6029	tr|I7GFH6|I7GFH6_MYCS2 CRP-like cAMP-activated global transcriptic
trxB	sp|O30973|TRXB_MYCSM Thioredoxin reductase OS=Mycolicibacteriu
rplO MSMEI_1438	tr|I7G436|I7G436_MYCS2 Large ribosomal subunit protein uL15 OS=
pgi	sp|P96803|G6PI_MYCSM Glucose-6-phosphate isomerase OS=Mycolic
rpsL	sp|P0C563|RS12_MYCSM Small ribosomal subunit protein uS12 OS=
sodC MSMEI_0816	tr|I7G2H6|I7G2H6_MYCS2 Superoxide dismutase [Cu-Zn] OS=Mycolic
rpsT MSMEG_4571 MSMEI_4	sp|A0R102|RS20_MYCS2 Small ribosomal subunit protein bS20 OS=M
MSMEG_0702	tr|A0QQC1|A0QQC1_MYCS2 Monooxygenase OS=Mycolicibacterium s
eltD MSMEG_3265 MSMEI_3	sp|A0QXD8|ELTD_MYCS2 Erythritol/L-threitol dehydrogenase OS=My
MSMEI_6127	tr|I7GAH8|I7GAH8_MYCS2 Transcription elongation factor OS=Mycoli
MSMEG_6392	tr|A0R617|A0R617_MYCS2 Polyketide synthase OS=Mycolicibacterium
MSMEI_3366	tr|I7GBA7|I7GBA7_MYCS2 Two-component system response regulato
D806_064810	tr|A0A2U9Q0F0|A0A2U9Q0F0_MYCSE Amine oxidase domain-containi
MSMEG_3580	tr|A0QY95|A0QY95_MYCS2 Antigen 85-C OS=Mycolicibacterium smeg
MSMEI_1422	tr|I7F8K7|I7F8K7_MYCS2 Coenzyme PQQ synthesis C OS=Mycolicibac
MSMEI_4989	tr|I7FRM7|I7FRM7_MYCS2 proline dehydrogenase OS=Mycolicibacteri
rpmD MSMEG_1473 MSMEI_	sp|A0QSG7|RL30_MYCS2 Large ribosomal subunit protein uL30 OS=
purL MSMEI_5666	tr|I7GEW9|I7GEW9_MYCS2 Phosphoribosylformylglycinamidine synth
MSMEG_3411	tr|A0QXS8|A0QXS8_MYCS2 MOSC domain protein OS=Mycolicibacteri
MSMEG_5152	tr|A0R2K9|A0R2K9_MYCS2 Uncharacterized protein OS=Mycolicibacte
pntAa MSMEI_0149	tr|I7F4S5|I7F4S5_MYCS2 NAD(P) transhydrogenase subunit alpha pa
adoK MSMEI_4169	tr|I7GBQ8|I7GBQ8_MYCS2 Carbohydrate kinase CbhK OS=Mycoliciba
yhfK BIN_B_01052	tr|A0A653FAJ8|A0A653FAJ8_MYCSM Putative sugar epimerase YhfK
MSMEI_3163	tr|I7G8V5|I7G8V5_MYCS2 Transcriptional regulatory protein PdtaR O
MSMEI_5679	tr|I7GFS6|I7GFS6_MYCS2 Glutathione peroxidase OS=Mycolicibacteri
pepA MSMEI_4179	tr|I7GBR5|I7GBR5_MYCS2 Probable cytosol aminopeptidase OS=Myc
MSMEG_1275	tr|A0QRX7|A0QRX7_MYCS2 HNH nuclease, putative OS=Mycolicibacte
D806_065570	tr|A0A2U9Q0A4|A0A2U9Q0A4_MYCSE Cache domain-containing prote
D806_020800	tr|A0A2U9PMS1|A0A2U9PMS1_MYCSE Phosphoenolpyruvate synthase

MSMEI_0998 MSMEI_2249	tr|I7G7T9|I7G7T9_MYCS2 Geranylgeranyl reductase OS=Mycolicibact
glyA MSMEG_5249 MSMEI_5	sp|A0R2V7|GLYA_MYCS2 Serine hydroxymethyltransferase OS=Mycol
MSMEI_5507	tr|I7G8G3|I7G8G3_MYCS2 Histidinol dehydrogenase OS=Mycolicibact
MSMEG_3809	tr|A0QYW3|A0QYW3_MYCS2 Luciferase-like domain-containing protei
MSMEI_5841	tr|I7GFY8|I7GFY8_MYCS2 Coa-transferase (Alpha subunit) OS=Mycol
D806_003960	tr|A0A2U9PI34|A0A2U9PI34_MYCSE MmpL protein OS=Mycolicibacter
lsr2 MSMEI_5934	tr|I7GA06|I7GA06_MYCS2 Nucleoid-associated protein Lsr2 OS=Myco
MSMEI_1034	tr|I7G4P8|I7G4P8_MYCS2 Glyoxalase/bleomycin resistance protein/di
MSMEG_6286 MSMEI_6121	sp|A0R5R7|Y6286_MYCS2 Putative aminotransferase MSMEG_6286/M
MSMEI_4266	tr|I7G509|I7G509_MYCS2 Transcriptional regulator, CdaR family OS=
MSMEI_5515	tr|I7FKY7|I7FKY7_MYCS2 Peptidyl-prolyl cis-trans isomerase OS=Myc
MSMEI_2207	tr|I7FZN9|I7FZN9_MYCS2 Nickel-dependent hydrogenase large subur
rpsS MSMEG_1440 MSMEI_1	sp|A0QSD5|RS19_MYCS2 Small ribosomal subunit protein uS19 OS=M
D806_058410	tr|A0A2U9PY83|A0A2U9PY83_MYCSE O-antigen polymerase, putative
MSMEI_3200	tr|I7FLQ8|I7FLQ8_MYCS2 Isocitrate dehydrogenase, NAD-dependent
D806_046560	tr|A0A2U9PV26|A0A2U9PV26_MYCSE DUF3618 domain-containing pro
ask	sp|P41403|AK_MYCSM Aspartokinase OS=Mycolicibacterium smegma
BIN_B_05650	tr|A0A653FPF2|A0A653FPF2_MYCSM Uncharacterized protein OS=My
MSMEI_0466	tr|I7FWG9|I7FWG9_MYCS2 Uncharacterized protein OS=Mycolicibacte
MSMEI_3951	tr|I7GCR7|I7GCR7_MYCS2 L-carnitine dehydratase/bile acid-inducible
MSMEI_3384	tr|I7G1Y7|I7G1Y7_MYCS2 Acyl-AMP synthetase OS=Mycolicibacterium
D806_044120	tr|A0A2U9PUF8|A0A2U9PUF8_MYCSE Thioester domain-containing pr
MSMEG_5269	tr|A0R2X7|A0R2X7_MYCS2 Uncharacterized protein OS=Mycolicibacte
MSMEI_4551	tr|I7FI02|I7FI02_MYCS2 Uncharacterized protein OS=Mycolicibacteriu
espK_2 BIN_B_05621	tr|A0A653FPX8|A0A653FPX8_MYCSM ESX-1 secretion-associated prot
mtrR_7 BIN_B_04741	tr|A0A653FLV3|A0A653FLV3_MYCSM HTH-type transcriptional regulat
MSMEI_2837	tr|I7G7S7|I7G7S7_MYCS2 GntR family transcriptional regulator OS=M
D806_053690	tr|A0A2U9PX01|A0A2U9PX01_MYCSE Peptidase S1 and S6, chymotryp
D806_000690	tr|A0A2U9PH71|A0A2U9PH71_MYCSE IS1096, tnpA protein OS=Myco
D806_061310	tr|A0A2U9PZ15|A0A2U9PZ15_MYCSE Thiopurine S-methyltransferase
MSMEI_5711	tr|I7GF02|I7GF02_MYCS2 Histidine triad (HIT) protein OS=Mycoliciba
MSMEI_5108	tr|I7G751|I7G751_MYCS2 NAD(P)H nitroreductase acg OS=Mycoliciba
purB MSMEI_5689	tr|I7GFT0|I7GFT0_MYCS2 Adenylosuccinate lyase OS=Mycolicibacteri
BIN_B_04911	tr|A0A653FME3|A0A653FME3_MYCSM Uncharacterized protein OS=M
MSMEI_0970	tr|I7F7A7|I7F7A7_MYCS2 Methyltransferase type 12 OS=Mycolicibact
D806_046770	tr|A0A2U9PV64|A0A2U9PV64_MYCSE Enoyl-CoA hydratase/isomerase
MSMEI_4101	tr|I7GD17|I7GD17_MYCS2 Phospholipid-binding protein, PBP family C

Number of proteins	Peptides	Razor + unique peptides	Sequence coverage [%]
4	26	26	62.9
4	18	18	40.6
10	30	30	50.8
3	23	23	70.8
2	13	13	99.0
3	14	14	55.0
3	24	24	39.9
2	16	16	45.2
3	16	16	28.1
7	14	14	37.1
5	10	10	26.1
3	13	13	44.7
2	13	13	26.0
4	9	9	27.7
3	10	10	43.1
3	9	9	38.9
4	15	15	29.3
3	9	9	61.1
4	12	12	34.4
2	5	5	38.4
2	12	12	37.5
3	11	11	62.3
2	10	10	53.5
3	9	9	25.4
3	9	9	43.7
3	10	10	17.8
2	11	11	33.1
4	9	9	30.3
3	13	13	31.4
3	7	7	77.6
3	11	11	26.3
2	8	8	30.5
2	6	6	52.4
4	11	11	29.9
2	10	10	20.1
3	3	3	14.2
3	6	6	52.6
2	7	7	38.5
4	6	6	55.9
3	8	8	20.8
3	10	10	45.0
3	6	6	39.3
3	10	10	16.8
3	9	9	26.5
3	8	8	18.9

3	5	5	72.6
4	8	8	32.0
4	7	7	31.6
3	8	8	17.7
3	8	8	24.3
2	6	6	41.3
2	7	7	27.9
3	5	5	35.4
3	6	6	22.3
4	8	8	14.3
3	6	6	27.1
4	7	7	20.7
2	10	10	42.9
4	5	5	18.0
4	5	5	56.7
3	8	8	10.0
3	3	3	9.3
2	6	6	19.9
2	6	6	57.3
4	5	5	6.7
3	8	8	26.6
2	8	8	48.0
2	7	7	54.7
2	5	5	26.0
3	3	3	17.7
2	6	6	22.1
2	7	7	25.8
3	3	3	11.2
2	5	5	30.8
4	5	5	14.3
4	4	4	39.0
3	4	4	32.8
6	3	3	17.9
3	3	3	51.1
3	2	2	6.9
2	5	5	33.1
4	2	2	3.2
3	3	3	22.4
2	4	4	40.0
3	3	3	15.3
3	5	5	11.8
3	4	4	39.7
2	4	4	31.4
4	3	3	13.9
4	2	2	50.9
3	3	3	10.0
2	3	3	37.2

2	2	2	31.0
3	6	6	19.1
2	3	3	19.8
3	2	2	13.5
3	3	3	21.8
5	1	1	9.7
4	3	3	24.5
3	1	1	7.5
4	3	3	7.9
3	3	3	4.6
2	3	3	9.9
4	2	2	8.6
3	4	4	25.8
2	3	3	27.6
3	3	3	6.7
3	5	3	8.3
3	3	3	22.2
2	2	2	12.3
2	3	3	29.5
2	3	3	33.1
4	4	4	10.1
2	2	2	38.2
2	2	2	19.7
1	3	3	19.0
2	2	2	12.4
4	2	2	21.0
2	2	2	20.2
4	4	4	17.5
4	2	2	13.6
3	2	2	7.9
4	3	3	9.4
3	2	2	10.6
2	2	2	12.4
2	1	1	14.3
3	1	1	7.1
2	2	2	12.6
2	2	2	13.8
2	3	3	5.6
3	1	1	6.3
2	2	2	24.8
3	1	1	6.8
4	2	2	5.5
3	2	2	7.5
3	1	1	13.2
5	2	2	5.1
3	2	2	9.2
2	2	2	7.3

2	2	2	12.1
4	2	2	11.7
3	2	2	3.5
4	2	2	11.2
3	2	2	55.2
3	2	2	3.5
3	1	1	5.5
3	1	1	11.2
2	2	2	17.4
4	2	2	5.2
2	1	1	6.3
2	1	1	13.0
3	1	1	6.4
3	1	1	5.9
4	1	1	2.6
3	1	1	15.2
4	1	1	2.3
2	1	1	14.6
3	1	1	6.7
4	1	1	6.8
3	2	1	19.7
4	2	2	7.1
3	1	1	6.5
3	1	1	7.6
2	2	2	34.9
3	2	2	12.6
2	1	1	3.0
3	2	2	17.8
4	2	2	2.1
3	1	1	3.7
1	1	1	1.4
3	1	1	5.5
4	1	1	3.3
3	2	2	8.8
1	1	1	27.9
4	1	1	1.4
2	1	1	5.5
1	2	2	7.9
3	1	1	2.2
3	1	1	4.6
4	1	1	3.3
4	2	2	11.5
2	1	1	7.5
3	1	1	1.7
3	2	2	5.8
4	1	1	3.4
1	2	2	7.8

3	2	2	5.6
2	1	1	2.4
3	1	1	2.0
2	1	1	3.4
4	1	1	3.1
4	2	2	1.5
3	1	1	11.4
3	1	1	8.1
3	1	1	2.8
4	1	1	3.3
3	1	1	7.5
4	1	1	2.8
2	1	1	8.6
1	1	1	3.5
4	2	2	8.1
3	1	1	21.3
4	1	1	3.6
1	1	1	3.2
2	1	1	2.9
3	1	1	2.6
4	2	2	6.8
1	1	1	5.3
1	1	1	5.0
1	1	1	37.8
4	1	1	4.4
3	1	1	7.0
3	1	1	6.0
1	1	1	2.3
1	1	1	5.0
3	1	1	8.1
3	1	1	15.2
4	1	1	2.7
3	1	1	1.5
3	1	1	2.6
2	1	1	5.7
3	1	1	4.2
3	1	1	13.3

				Volcar
**Mol. weight [kDa]**	**Q-value**	**Score**	**FC**	**log2(FC)**
46.276	0	182.9	2.2207	1.151
58.43	0	155.98	0.6553	-0.60978
56.487	0	149.83	1.279	0.35502
33.819	0	132.21	0.61781	-0.69476
10.763	0	95.522	0.56193	-0.83153
35.947	0	95.425	0.76971	-0.37762
66.646	0	86.504	0.56353	-0.82744
40.932	0	76.607	0.78467	-0.34984
71.211	0	76.417	1.7009	0.76634
55.175	0	74.6	1.9369	0.95376
60.432	0	72.989	1.9755	0.98225
43.735	0	72.592	3.0968	1.6308
53.27	0	68.194	0.7785	-0.36124
42.591	0	61.817	0.51065	-0.96959
32.013	0	59.245	1.1914	0.25262
21.23	0	55.672	82.267	6.3622
75.154	0	54.149	0.68877	-0.5379
18.726	0	53.555	0.61708	-0.69648
49.762	0	52.256	0.57859	-0.78938
10.737	0	51.472	0.76804	-0.38074
44.761	0	48.727	0.7499	-0.41523
13.458	0	48.576	0.79526	-0.3305
29.405	0	47.552	1.0533	0.074965
54.871	0	47.21	0.74216	-0.43019
27.787	0	47.026	0.94981	-0.074283
82.603	0	46.95	0.46218	-1.1135
51.616	0	44.464	1.8004	0.8483
34.352	0	44.321	0.66019	-0.59904
63.137	0	42.699	1.8613	0.89633
7.3471	0	42.155	0.77005	-0.37699
54.954	0	38.739	0.82617	-0.27549
29.544	0	37.215	0.66226	-0.59454
11.635	0	33.485	2.8304	1.501
53.315	0	33.41	4.5457	2.1845
58.888	0	33.179	1.6649	0.73544
30.002	0	33.008	6.9114	2.789
17.628	0	32.313	25.884	4.694
30.357	0	32.158	6.8246	2.7707
15.849	0	31.222	0.78669	-0.34613
59.357	0	30.884	3.0646	1.6157
30.948	0	30.271	0.70711	-0.5
15.927	0	29.893	0.90493	-0.14412
81.026	0	27.309	0.90569	-0.14292
44.654	0	26.045	1.0488	0.068749
52.259	0	25.944	0.55191	-0.8575

16.832	0	24.638	1.3266	0.40773
31.925	0	23.714	0.99399	-0.0087029
31.022	0	23.474	0.93533	-0.096447
66.943	0	23.273	0.58314	-0.77809
53.591	0	22.615	0.46405	-1.1076
19.453	0	22.223	1.6687	0.73869
36.421	0	21.569	1.352	0.43513
13.684	0	20.577	9.4291	3.2371
33.972	0	20.557	0.66567	-0.58712
81.113	0	20.216	0.7795	-0.35938
38.767	0	20.214	1.0012	0.0016736
48.521	0	20.065	1.0341	0.048425
37.919	0	19.997	3.4968	1.806
38.454	0	19.769	1.748	0.80572
13.144	0	19.752	0.85312	-0.22918
102.17	0	19.466	1.3419	0.42431
49.413	0	19.279	0.39728	-1.3318
42.094	0	19.161	0.66116	-0.59693
18.005	0	18.977	0.59336	-0.75302
94.097	0	18.917	0.73633	-0.44158
42.59	0	18.729	1.0541	0.076023
18.011	0	18.179	1.7032	0.76822
16.765	0	17.971	7.0268	2.8129
25.019	0	17.874	4.1371	2.0486
16.584	0	16.282	0.34379	-1.5404
39.935	0	15.303	1.8862	0.91552
22.972	0	15.29	12.3	3.6206
47.089	0	15.172	2.4352	1.284
23.375	0	15.114	7.5666	2.9197
50.784	0	14.837	0.49555	-1.0129
16.934	0	13.947	0.61628	-0.69835
21.626	0	13.309	3.8064	1.9284
22.935	0	13.253	0.13957	-2.841
13.84	0	12.729	1.2794	0.35542
30.221	0	12.477	#N/A	#N/A
14.217	0	12.442	18.284	4.1925
100.84	0	12.163	2.9772	1.5739
11.67	0	11.42	1.0664	0.092753
10.185	0	11.412	2.5151	1.3306
22.871	0	11.041	2.7591	1.4642
81.997	0	10.55	0.43333	-1.2064
16.768	0	10.467	21.134	4.4015
11.206	0	10.39	3.6975	1.8866
30.338	0	10.376	3.2306	1.6918
5.6485	0	10.346	0.53341	-0.90668
46.498	0	10.3	4.355	2.1227
14.464	0	10.23	2.8049	1.4879

11.028	0	10.209	46.5	5.5392
45.164	0	9.9062	0.45451	-1.1376
21.122	0	9.8384	90.811	6.5048
21.508	0	9.8333	2.3361	1.2241
16.118	0	9.7844	20.287	4.3425
15.646	0	9.2064	#N/A	#N/A
11.51	0	9.0233	0.47063	-1.0873
18.482	0	8.8797	0.90632	-0.14191
61.029	0	8.7265	4.8307	2.2722
78.276	0	8.7072	0.7199	-0.47413
40.426	0	8.3669	0.55736	-0.84331
33.696	0	8.3341	1.1238	0.16843
23.77	0	8.3179	0.63174	-0.66259
11.102	0	7.8356	34.044	5.0893
60.117	0	7.8324	0.83391	-0.26204
56.152	0	7.7419	7.4576	2.8987
16.323	0	7.7038	58.804	5.8778
30.107	0	7.479	1.7963	0.845
14.47	0	6.7854	21.411	4.4203
13.265	0	6.2134	1.377	0.46157
53.388	0	6.1171	0.96323	-0.054044
10.344	0	6.0233	25.892	4.6944
15.002	0	5.8906	3.932	1.9753
15.573	0	5.8023	2.7148	1.4408
20.824	0	5.7417	0.63238	-0.66113
12.029	0	5.7037	#N/A	#N/A
13.362	0	5.6989	1.6075	0.68479
37.302	0	5.6786	2.7378	1.453
13.344	0	5.6479	0.18266	-2.4527
51.655	0	5.4381	1.3223	0.40304
38.404	0	5.4246	0.64837	-0.6251
15.923	0	5.3956	5.7591	2.5258
29.675	0	5.3862	3.9746	1.9908
9.502	0	5.2852	21.149	4.4025
20.141	0	5.2463	2.2573	1.1746
18.944	0	5.0823	0.62773	-0.67179
19.871	0	5.0766	0.46229	-1.1131
103.08	0	4.9991	1.6156	0.69204
35.701	0	4.9095	22.97	4.5217
11.433	0	4.5507	4.4738	2.1615
21.649	0	4.5117	#N/A	#N/A
54.303	0	4.4638	0.74919	-0.41659
32.85	0	4.3201	0.22775	-2.1345
20.038	0	4.2702	5.4835	2.4551
77.364	0	4.0149	0.89665	-0.15738
24.066	0	3.9607	3.5618	1.8326
30.139	0	3.9321	#N/A	#N/A

17.401	0	3.8114	14.976	3.9046
18.349	0	3.7489	0.82027	-0.28584
64.621	0	3.7389	0.047922	-4.3832
26.449	0	3.6437	1.2207	0.28769
7.1048	0	3.6335	0.41564	-1.2666
51.812	0	3.6244	0.74034	-0.43374
20.27	0	3.4181	3.9332	1.9757
15.069	0	3.3669	2.4977	1.3206
15.742	0	3.315	#N/A	#N/A
49.469	0	3.2323	1.7291	0.79001
22.899	0	3.2271	14.858	3.8931
8.7778	0	3.2187	3.9708	1.9894
26.668	0	3.2011	0.029557	-5.0803
34.042	0	3.0744	0.04634	-4.4316
62.038	0	3.046	#N/A	#N/A
8.4474	0	3.0406	19.745	4.3034
47.199	0	3.0353	#N/A	#N/A
11.034	0	3.0135	#N/A	#N/A
24.775	0	2.8453	6.742	2.7532
33.611	0	2.7736	1.5225	0.6064
15.571	0.0061728	2.7068	7.5954	2.9251
59.672	0.006135	2.6987	#N/A	#N/A
13.863	0.0060976	2.671	23.502	4.5547
23.213	0.0060606	2.5954	#N/A	#N/A
9.5329	0.0060241	2.5547	58.925	5.8808
33.464	0.005988	2.5123	0.046351	-4.4312
39.356	0.0059524	2.5038	0.26167	-1.9342
17.078	0.0059172	2.5011	2.0302	1.0216
194.46	0.0058824	2.4543	0.89453	-0.1608
23.459	0.005848	2.4406	#N/A	#N/A
62.643	0.005814	2.3674	7.1531	2.8386
35.915	0.017341	2.2581	0.048301	-4.3718
27.36	0.022989	2.2535	0.44313	-1.1742
35.421	0.022857	2.2462	3.3951	1.7635
7.008	0.028409	2.213	1.8323	0.87365
81.011	0.028249	2.1486	11.48	3.521
15.51	0.02809	2.1177	1.9374	0.95415
24.463	0.027933	2.064	#N/A	#N/A
37.307	0.033333	2.0197	0.044766	-4.4815
34.854	0.033149	1.9946	#N/A	#N/A
21.937	0.038462	1.9608	#N/A	#N/A
23.173	0.038251	1.9469	0.0031733	-8.2998
17.203	0.038043	1.9405	#N/A	#N/A
53.469	0.043243	1.8481	#N/A	#N/A
39.607	0.043011	1.8314	0.41699	-1.2619
26.49	0.042781	1.8174	#N/A	#N/A
37.935	0.047872	1.784	#N/A	#N/A

43.095	0.047619	1.779	0.25249	-1.9857
51.904	0.04712	1.7737	3.2847	1.7157
47.779	0.046875	1.7699	18.548	4.2132
28.479	0.046632	1.7699	0.186	-2.4266
32.478	0.056701	1.7472	#N/A	#N/A
106.39	0.05641	1.7221	#N/A	#N/A
12.454	0.056122	1.719	14.271	3.835
13.712	0.055838	1.6711	#N/A	#N/A
46.41	0.055556	1.6271	0.52042	-0.94226
45.18	0.055276	1.6181	#N/A	#N/A
12.901	0.054726	1.6141	1.4292	0.51524
59.617	0.054455	1.6059	#N/A	#N/A
10.773	0.054187	1.6052	27.359	4.774
49.139	0.058824	1.5643	2.5318	1.3402
37.759	0.058537	1.5566	#N/A	#N/A
8.2275	0.067633	1.5046	#N/A	#N/A
44.458	0.072115	1.4774	#N/A	#N/A
25.118	0.07177	1.4502	#N/A	#N/A
26.745	0.071429	1.4425	7.3314	2.8741
40.904	0.07109	1.4383	0.32724	-1.6116
65.801	0.075472	1.4188	0.85631	-0.2238
36.087	0.079812	1.3847	#N/A	#N/A
18.502	0.079439	1.358	#N/A	#N/A
5.0057	0.07907	1.353	#N/A	#N/A
25.344	0.083333	1.3277	1.4491	0.53517
22.661	0.082949	1.2929	#N/A	#N/A
25.796	0.087156	1.2749	#N/A	#N/A
44.538	0.086758	1.2656	#N/A	#N/A
48.974	0.090909	1.2606	#N/A	#N/A
24.225	0.090498	1.2606	#N/A	#N/A
15.156	0.09009	1.2588	0.37463	-1.4165
36.22	0.09417	1.241	#N/A	#N/A
51.358	0.09375	1.2329	18.867	4.2378
33.747	0.093333	1.2291	#N/A	#N/A
22.332	0.09292	1.2272	1.6117	0.68856
24.931	0.092511	1.2158	28.651	4.8405
18.061	0.092105	1.2156	1.0396	0.055985

ıo plot

raw.pval	-LOG10(p)	Avg. LFQ intensity Cell	MIC	LFQ intensity Cell01
0.000057282	4.242	39,253.00	17,676.33	39,262.00
0.0065116	2.1863	19,850.00	30,291.67	22,526.00
0.028636	1.5431	20,576.67	16,088.00	18,682.00
0.12155	0.91524	13,216.47	21,392.33	9,289.40
0.073717	1.1324	43,475.67	77,368.00	24,603.00
0.0048289	2.3162	30,008.33	38,986.67	30,040.00
0.10845	0.96478	9,220.07	16,361.33	4,899.20
0.73672	0.1327	11,127.93	14,181.63	7,230.80
0.20066	0.69753	16,034.33	9,426.70	23,762.00
0.011689	1.9322	22,254.67	11,489.73	23,068.00
0.01984	1.7025	8,335.77	4,219.47	6,709.60
0.0095671	2.0192	19,491.33	6,294.10	24,007.00
0.39322	0.40537	10,519.17	13,512.13	7,409.50
0.0042598	2.3706	7,784.20	15,243.67	7,300.20
0.667	0.17587	20,600.00	17,291.00	11,733.00
0.013523	1.8689	36,279.67	399.89	33,857.00
0.072636	1.1389	9,424.50	13,683.00	11,656.00
0.18077	0.74286	10,711.43	17,358.33	7,310.30
0.012421	1.9058	6,713.07	11,602.43	5,718.90
0.70824	0.14982	33,180.93	43,202.00	7,149.80
0.91918	0.036598	7,253.13	9,672.13	8,784.40
0.15775	0.80202	34,492.67	43,372.67	34,246.00
0.74021	0.13064	8,521.17	8,089.70	7,422.50
0.037628	1.4245	7,752.53	10,445.87	7,867.10
0.7313	0.1359	8,936.77	9,408.97	5,418.40
0.00081764	3.0874	7,572.77	16,385.00	8,458.20
0.046823	1.3295	10,596.83	5,885.90	11,169.00
0.32734	0.48501	13,249.63	20,069.33	8,603.90
0.01981	1.7031	8,125.70	4,365.53	7,497.00
0.57807	0.23802	12,123.00	15,743.23	11,653.00
0.33729	0.472	15,289.67	18,506.67	21,828.00
0.10781	0.96735	7,577.10	11,441.33	9,954.90
0.0025002	2.602	27,361.67	9,667.10	31,280.00
0.18452	0.73396	8,068.77	1,733.93	3,681.30
0.16225	0.78981	10,083.13	6,056.30	13,195.00
0.393	0.4056	4,534.90	579.87	0.00
0.0063601	2.1965	12,196.10	471.18	20,547.00
0.0032264	2.4913	8,711.47	1,276.49	6,652.60
0.70979	0.14887	8,448.60	10,739.40	7,038.10
0.036277	1.4404	6,561.50	2,141.03	5,086.30
0.06053	1.218	12,298.00	17,392.00	13,146.00
0.57521	0.24017	13,845.10	15,299.67	1,839.30
0.69072	0.1607	6,064.93	6,696.50	6,264.70
0.96338	0.016202	7,389.37	7,045.50	4,690.70
0.088129	1.0549	4,916.83	8,908.80	3,075.00

0.14828	0.82893	6,674.93	5,031.63	5,912.70
0.98574	0.0062395	9,156.13	9,211.53	6,928.20
0.85921	0.065903	8,892.50	9,507.30	7,606.50
0.032108	1.4934	3,947.70	6,769.73	2,830.60
0.002099	2.678	5,161.50	11,122.73	5,129.90
0.071661	1.1447	22,336.67	13,386.00	29,808.00
0.15979	0.79644	8,114.37	6,001.60	8,795.50
0.058287	1.2344	8,536.43	823.10	13,311.00
0.080581	1.0938	6,886.13	10,344.63	7,881.70
0.35771	0.44646	4,954.80	6,356.37	2,445.50
0.95577	0.019645	6,785.10	6,777.23	6,168.80
0.6867	0.16323	7,889.93	7,629.50	8,197.20
0.22655	0.64483	9,055.80	2,548.60	6,024.40
0.10747	0.96871	8,011.17	4,583.00	9,141.30
0.4116	0.38553	11,038.20	12,938.67	13,637.00
0.95816	0.018561	3,877.07	2,889.17	966.82
0.085596	1.0675	2,558.23	6,439.30	2,412.80
0.25355	0.59593	3,719.03	5,625.03	1,694.60
0.3746	0.42643	3,615.33	6,162.27	0.00
0.27846	0.55524	4,094.73	5,561.03	3,874.00
0.74845	0.12584	6,766.57	6,419.23	5,417.00
0.0033768	2.4715	6,199.77	3,640.13	6,738.10
0.071894	1.1433	10,408.00	1,398.97	6,892.00
0.19239	0.71582	5,004.13	1,168.47	4,198.20
0.11607	0.93527	2,159.93	6,521.87	0.00
0.65141	0.18615	5,042.82	2,673.47	979.37
0.050525	1.2965	6,969.37	484.37	13,040.00
0.039042	1.4085	10,803.83	4,436.60	14,524.00
0.10163	0.99299	7,324.47	926.88	7,654.90
0.33277	0.47785	2,460.83	5,048.87	0.00
0.67377	0.17149	3,275.67	5,315.27	5,087.10
0.093968	1.027	3,346.30	796.90	3,071.00
0.86031	0.065344	2,027.21	14,737.33	5,456.10
0.18798	0.72589	5,587.87	4,367.70	4,361.80
#N/A	#N/A	0.00	0.00	0.00
0.11649	0.93371	2,214.03	0.00	0.00
0.10986	0.95917	2,358.20	709.87	2,538.00
0.98697	0.0056951	4,059.93	3,804.57	0.00
0.27278	0.56419	4,961.30	1,931.50	5,850.50
0.23525	0.62847	3,648.50	1,281.25	2,688.10
0.0056987	2.2442	3,184.80	7,349.53	2,928.70
0.11718	0.93114	2,565.53	0.00	0.00
0.19034	0.72046	3,450.03	891.95	1,976.20
0.24551	0.60992	4,449.27	1,336.10	1,757.70
0.66424	0.17768	2,802.20	5,212.23	3,221.50
0.17948	0.74598	5,702.80	1,268.37	7,910.40
0.80135	0.09618	3,040.90	1,057.70	5,766.50

5.4223E-06	5.2658	5,735.37	0.00	4,687.30
0.35734	0.44691	3,239.73	7,127.90	3,281.90
3.5699E-08	7.4473	11,200.67	0.00	12,384.00
0.27998	0.55287	4,599.73	1,927.87	4,792.60
0.026929	1.5698	6,799.53	252.94	11,400.00
#N/A	#N/A	0.00	0.00	0.00
0.3064	0.51372	3,487.03	7,496.70	0.00
0.89585	0.047764	5,170.37	5,704.80	2,320.50
0.080443	1.0945	4,208.67	789.00	4,420.20
0.098548	1.0064	4,420.77	6,140.80	5,796.60
0.33596	0.47371	3,053.90	5,552.97	4,576.00
0.44888	0.34787	3,006.13	2,633.77	1,583.50
0.16725	0.77664	3,929.93	6,220.77	5,418.60
8.1495E-07	6.0889	4,198.93	0.00	4,055.30
0.42474	0.37188	2,307.40	2,816.27	3,087.80
0.060178	1.2206	4,638.07	539.70	2,962.00
1.0416E-06	5.9823	7,252.87	0.00	5,596.20
0.083926	1.0761	6,292.30	3,503.00	7,530.90
3.6708E-08	7.4352	2,640.87	0.00	2,893.40
0.019011	1.721	4,793.67	3,481.13	4,275.30
0.76401	0.1169	4,790.73	4,973.60	4,189.30
0.11924	0.92357	3,152.37	0.00	2,481.60
0.73721	0.13241	3,711.80	913.33	0.00
0.48012	0.31865	2,658.47	912.17	0.00
0.1479	0.83004	4,357.93	6,891.27	4,942.70
#N/A	#N/A	0.00	0.00	0.00
0.34857	0.45771	3,543.53	2,163.30	3,025.90
0.12014	0.92032	3,959.07	1,363.83	4,285.50
0.074916	1.1254	634.10	3,921.57	0.00
0.40334	0.39432	3,812.53	2,842.17	3,071.90
0.086546	1.0628	3,216.07	4,960.20	3,533.70
0.41048	0.38671	3,874.43	597.67	0.00
0.4387	0.35784	2,612.57	585.43	0.00
0.024365	1.6132	7,966.27	294.44	9,680.10
0.50247	0.29889	2,511.23	1,048.50	0.00
0.35822	0.44585	2,817.37	4,553.70	0.00
0.50521	0.29653	1,099.27	2,514.63	0.00
0.24077	0.61839	6,730.23	4,165.87	9,771.40
0.1162	0.93481	2,792.07	0.00	0.00
0.083762	1.077	2,683.03	517.50	2,309.80
#N/A	#N/A	0.00	0.00	0.00
0.25505	0.59337	3,280.93	4,379.30	2,994.90
0.42173	0.37497	369.17	1,940.83	1,107.50
0.10031	0.99866	6,223.23	1,052.67	12,838.00
0.5813	0.2356	1,359.60	1,566.90	0.00
0.0953	1.0209	2,508.67	622.10	2,296.80
#N/A	#N/A	0.00	0.00	0.00

0.11809	0.92779	1,806.03	0.00	0.00
0.95421	0.020357	1,254.23	1,538.07	0.00
0.12589	0.90002	0.00	2,532.67	0.00
0.42094	0.37578	2,513.00	2,017.57	2,477.50
0.088048	1.0553	2,700.17	6,496.37	1,450.10
0.24517	0.61054	155,320.67	209,796.67	245,380.00
0.43528	0.36123	1,680.43	355.47	0.00
0.12547	0.90145	2,363.17	863.90	1,777.40
#N/A	#N/A	0.00	0.00	0.00
0.88849	0.051348	1,797.97	1,022.50	0.00
0.1166	0.93331	1,791.43	0.00	0.00
0.44232	0.35427	1,656.90	345.40	0.00
0.000028633	4.5431	0.00	4,172.93	0.00
0.12047	0.91911	0.00	2,620.53	0.00
#N/A	#N/A	0.00	0.00	0.00
0.000014653	4.8341	2,435.30	0.00	1,856.10
#N/A	#N/A	0.00	0.00	0.00
#N/A	#N/A	0.00	0.00	0.00
0.060063	1.2214	3,736.13	471.93	3,254.90
0.54332	0.26495	2,142.13	1,351.80	0.00
0.067639	1.1698	4,754.17	543.70	1,845.10
#N/A	#N/A	0.00	0.00	0.00
0.13184	0.87995	2,857.60	0.00	7,677.10
#N/A	#N/A	0.00	0.00	0.00
0.000070606	4.1512	7,267.77	0.00	13,045.00
0.11627	0.93454	0.00	2,619.87	0.00
0.0005285	3.277	1,105.38	4,224.27	1,146.90
0.90102	0.045264	605.77	256.65	0.00
0.50876	0.29349	2,886.43	3,226.77	3,923.00
#N/A	#N/A	0.00	0.00	0.00
0.071919	1.1432	14,160.00	1,897.33	16,162.00
0.11612	0.93511	0.00	2,512.43	0.00
0.86535	0.062809	1,321.77	3,034.47	2,534.20
0.44535	0.3513	1,723.43	437.50	0.00
0.52814	0.27725	2,885.17	1,514.83	5,514.20
0.11717	0.93119	1,374.80	0.00	0.00
0.92194	0.035299	934.27	442.43	0.00
#N/A	#N/A	0.00	0.00	0.00
0.11612	0.93509	0.00	2,714.10	0.00
#N/A	#N/A	0.00	0.00	0.00
#N/A	#N/A	0.00	0.00	0.00
0.00003121	4.5057	0.00	38,867.67	0.00
#N/A	#N/A	0.00	0.00	0.00
#N/A	#N/A	0.00	0.00	0.00
0.51224	0.29053	1,167.77	2,956.57	3,503.30
#N/A	#N/A	0.00	0.00	0.00
#N/A	#N/A	0.00	0.00	0.00

0.45322	0.34369	1,033.83	4,379.17	3,101.50
0.10053	0.99769	2,151.77	572.87	1,846.80
4.6069E-06	5.3366	2,287.67	0.00	2,171.10
0.17277	0.76254	808.20	4,566.23	0.00
#N/A	#N/A	0.00	0.00	0.00
#N/A	#N/A	0.00	0.00	0.00
0.12183	0.91426	1,719.03	0.00	0.00
#N/A	#N/A	0.00	0.00	0.00
0.93579	0.028822	894.73	1,795.03	0.00
#N/A	#N/A	0.00	0.00	0.00
0.92822	0.032348	2,329.10	1,617.27	0.00
#N/A	#N/A	0.00	0.00	0.00
5.0706E-07	6.2949	3,374.50	0.00	4,046.80
0.14703	0.83258	4,416.03	1,662.00	2,124.30
#N/A	#N/A	0.00	0.00	0.00
#N/A	#N/A	0.00	0.00	0.00
#N/A	#N/A	0.00	0.00	0.00
#N/A	#N/A	0.00	0.00	0.00
0.063474	1.1974	2,939.53	318.73	1,705.30
0.072976	1.1368	3,344.77	10,221.07	5,303.00
0.97624	0.010444	8,661.33	10,121.67	11,815.00
#N/A	#N/A	0.00	0.00	0.00
#N/A	#N/A	0.00	0.00	0.00
#N/A	#N/A	0.00	0.00	0.00
0.6562	0.18296	1,038.03	731.95	0.00
#N/A	#N/A	0.00	0.00	0.00
#N/A	#N/A	0.00	0.00	0.00
#N/A	#N/A	0.00	0.00	0.00
#N/A	#N/A	0.00	0.00	0.00
#N/A	#N/A	0.00	0.00	0.00
0.4856	0.31372	784.23	2,271.73	2,352.70
#N/A	#N/A	0.00	0.00	0.00
0.11723	0.93096	2,285.90	0.00	4,414.20
#N/A	#N/A	0.00	0.00	0.00
0.36219	0.44106	4,981.53	3,049.80	6,605.90
0.12038	0.91944	3,492.70	0.00	8,088.80
0.99589	0.0017908	964.30	924.47	0.00

LFQ intensity Cell02	LFQ intensity Cell03	LFQ intensity MIC01	LFQ intensity MIC02
41,542.00	36,955.00	18,398.00	17,935.00
18,896.00	18,128.00	31,928.00	27,504.00
23,059.00	19,989.00	15,426.00	15,504.00
15,850.00	14,510.00	17,978.00	29,425.00
58,731.00	47,093.00	71,690.00	77,120.00
28,828.00	31,157.00	37,414.00	37,358.00
10,031.00	12,730.00	19,395.00	16,580.00
13,969.00	12,184.00	18,520.00	18,199.00
10,822.00	13,519.00	6,738.40	6,392.70
21,796.00	21,900.00	11,519.00	14,463.00
9,805.80	8,491.90	5,304.20	4,163.00
17,292.00	17,175.00	9,185.00	4,290.80
15,237.00	8,911.00	12,995.00	17,879.00
7,742.80	8,309.60	14,806.00	12,644.00
23,889.00	26,178.00	20,163.00	16,333.00
40,490.00	34,492.00	0.00	823.75
8,239.50	8,378.00	13,080.00	11,483.00
13,739.00	11,085.00	18,714.00	22,607.00
6,920.80	7,499.50	9,471.30	12,713.00
57,218.00	35,175.00	55,118.00	63,161.00
6,507.50	6,467.50	11,807.00	2,561.40
31,067.00	38,165.00	47,227.00	34,250.00
10,380.00	7,761.00	7,031.80	7,940.60
8,717.00	6,673.50	10,076.00	9,572.60
12,219.00	9,172.90	9,532.30	7,739.60
6,540.80	7,719.30	16,069.00	15,410.00
8,760.50	11,861.00	7,295.50	3,859.80
15,333.00	15,812.00	22,049.00	27,294.00
9,453.20	7,426.90	4,386.20	5,457.90
12,960.00	11,756.00	19,296.00	19,958.00
11,295.00	12,746.00	20,143.00	18,960.00
5,184.50	7,591.90	10,003.00	10,651.00
28,715.00	22,090.00	9,059.40	8,086.90
9,438.00	11,087.00	3,756.00	0.00
7,312.40	9,742.00	7,527.10	3,306.90
7,285.10	6,319.60	0.00	1,739.60
3,905.30	12,136.00	343.08	246.68
6,699.80	12,782.00	1,167.50	863.06
8,310.00	9,997.70	12,479.00	14,879.00
5,042.40	9,555.80	1,070.70	2,472.60
11,492.00	12,256.00	19,833.00	18,910.00
25,171.00	14,525.00	15,962.00	20,618.00
5,362.10	6,568.00	6,941.20	4,758.80
8,611.90	8,865.50	7,627.00	7,098.10
7,410.20	4,265.30	8,990.90	10,810.00

6,972.00	7,140.10	5,321.40	6,158.70
10,676.00	9,864.20	6,478.50	9,775.10
9,794.40	9,276.60	10,068.00	6,387.90
3,905.40	5,107.10	6,431.40	6,540.80
4,411.60	5,943.00	12,135.00	11,507.00
21,021.00	16,181.00	11,062.00	15,282.00
7,024.60	8,523.00	4,629.30	5,128.10
6,942.50	5,355.80	0.00	0.00
5,992.10	6,784.60	10,892.00	7,606.90
7,675.10	4,743.80	5,958.30	7,120.30
8,395.70	5,790.80	7,509.00	4,820.90
7,353.60	8,119.00	4,728.80	12,777.00
10,879.00	10,264.00	4,053.10	3,592.70
6,191.50	8,700.70	4,486.50	2,613.50
10,280.00	9,197.60	10,712.00	11,874.00
3,523.40	7,141.00	2,030.80	2,872.60
1,846.20	3,415.70	8,013.90	8,301.30
5,359.30	4,103.20	5,903.20	6,784.00
4,585.80	6,260.20	8,421.50	7,347.50
5,064.10	3,346.10	3,673.30	6,917.00
8,141.50	6,741.20	6,816.60	8,529.50
6,544.60	5,316.60	3,497.50	3,483.10
14,230.00	10,102.00	0.00	4,196.90
2,982.40	7,831.80	1,950.20	0.00
6,479.80	0.00	6,879.80	6,956.50
9,015.60	5,133.50	1,576.70	3,693.80
3,019.60	4,848.50	1,453.10	0.00
8,801.10	9,086.40	3,084.00	3,244.50
5,817.60	8,500.90	716.94	0.00
3,191.40	4,191.10	7,532.20	5,143.00
2,485.20	2,254.70	2,376.50	2,350.30
3,165.90	3,802.00	0.00	2,390.70
0.00	625.52	0.00	0.00
6,587.00	5,814.80	5,181.70	3,934.60
0.00	0.00	0.00	0.00
2,758.30	3,883.80	0.00	0.00
1,879.40	2,657.20	0.00	0.00
6,718.80	5,461.00	6,723.20	4,690.50
5,891.10	3,142.30	2,469.20	0.00
2,685.50	5,571.90	988.25	0.00
3,489.60	3,136.10	9,518.70	6,571.50
2,743.80	4,952.80	0.00	0.00
3,959.20	4,414.70	932.44	0.00
5,754.50	5,835.60	1,716.00	0.00
3,927.50	1,257.60	8,514.10	7,122.60
4,442.60	4,755.40	1,753.00	2,052.10
0.00	3,356.20	1,770.30	0.00

4,471.30	8,047.50	0.00	0.00
3,033.60	3,403.70	3,399.30	3,213.40
10,067.00	11,151.00	0.00	0.00
5,924.30	3,082.30	2,069.80	3,713.80
3,668.60	5,330.00	758.81	0.00
0.00	0.00	0.00	0.00
5,000.10	5,461.00	6,482.30	6,872.40
5,762.50	7,428.10	5,753.10	2,398.50
4,101.20	4,104.60	0.00	2,367.00
3,911.90	3,553.80	6,906.50	5,904.80
4,585.70	0.00	5,539.40	4,721.40
4,367.70	3,067.20	5,480.10	2,421.20
3,196.60	3,174.60	6,167.60	4,153.90
5,083.30	3,458.20	0.00	0.00
0.00	3,834.40	3,312.00	2,550.00
6,141.80	4,810.40	1,619.10	0.00
8,726.90	7,435.50	0.00	0.00
4,438.70	6,907.30	2,834.10	2,614.80
2,548.60	2,480.60	0.00	0.00
4,617.50	5,488.20	3,738.00	3,415.50
5,371.60	4,811.30	5,645.60	4,227.30
6,975.50	0.00	0.00	0.00
4,605.90	6,529.50	0.00	1,389.20
3,778.40	4,197.00	0.00	0.00
3,796.80	4,334.30	4,466.30	6,644.40
0.00	0.00	0.00	0.00
3,657.60	3,947.10	2,347.40	0.00
3,013.30	4,578.40	0.00	0.00
0.00	1,902.30	4,041.40	5,098.70
5,142.80	3,222.90	5,127.70	0.00
2,478.70	3,635.80	4,460.50	4,001.30
4,245.10	7,378.20	0.00	0.00
3,151.30	4,686.40	0.00	1,756.30
6,526.90	7,691.80	0.00	883.33
4,545.30	2,988.40	3,145.50	0.00
4,547.70	3,904.40	4,752.50	4,017.10
0.00	3,297.80	3,395.50	4,148.40
4,853.20	5,566.10	7,205.00	2,949.20
3,843.40	4,532.80	0.00	0.00
3,848.30	1,891.00	0.00	1,552.50
0.00	0.00	0.00	0.00
3,773.50	3,074.40	3,752.30	6,046.30
0.00	0.00	3,889.00	0.00
2,205.20	3,626.50	0.00	0.00
0.00	4,078.80	2,598.50	2,102.20
2,551.70	2,677.50	0.00	1,866.30
0.00	0.00	0.00	0.00

3,680.40	1,737.70	0.00	0.00
2,557.40	1,205.30	3,035.30	0.00
0.00	0.00	0.00	1,171.30
2,220.50	2,841.00	3,198.40	0.00
2,955.40	3,695.00	6,994.00	8,918.10
98,512.00	122,070.00	193,670.00	218,810.00
3,578.50	1,462.80	1,066.40	0.00
2,802.40	2,509.70	2,591.70	0.00
0.00	0.00	0.00	0.00
3,322.40	2,071.50	1,644.50	1,423.00
2,191.30	3,183.00	0.00	0.00
3,811.00	1,159.70	0.00	1,036.20
0.00	0.00	3,492.50	6,448.70
0.00	0.00	1,855.00	6,006.60
0.00	0.00	0.00	0.00
3,523.20	1,926.60	0.00	0.00
0.00	0.00	0.00	0.00
0.00	0.00	0.00	0.00
4,586.00	3,367.50	0.00	0.00
3,162.80	3,263.60	4,055.40	0.00
7,285.00	5,132.40	0.00	0.00
0.00	0.00	0.00	0.00
895.70	0.00	0.00	0.00
0.00	0.00	0.00	0.00
4,056.10	4,702.20	0.00	0.00
0.00	0.00	4,369.20	3,490.40
875.04	1,294.20	3,769.90	4,178.30
0.00	1,817.30	769.94	0.00
2,468.20	2,268.10	2,973.60	3,744.10
0.00	0.00	0.00	0.00
10,496.00	15,822.00	0.00	0.00
0.00	0.00	3,760.90	3,776.40
1,431.10	0.00	2,088.80	7,014.60
2,175.70	2,994.60	1,312.50	0.00
0.00	3,141.30	0.00	0.00
1,534.30	2,590.10	0.00	0.00
0.00	2,802.80	1,327.30	0.00
0.00	0.00	0.00	0.00
0.00	0.00	3,999.60	4,142.70
0.00	0.00	0.00	0.00
0.00	0.00	0.00	0.00
0.00	0.00	25,611.00	20,581.00
0.00	0.00	0.00	0.00
0.00	0.00	0.00	0.00
0.00	0.00	0.00	2,193.30
0.00	0.00	0.00	0.00
0.00	0.00	0.00	0.00

0.00	0.00	4,518.80	0.00
2,045.60	2,562.90	1,718.60	0.00
1,736.60	2,955.30	0.00	0.00
1,464.30	960.29	5,903.30	6,439.10
0.00	0.00	0.00	0.00
0.00	0.00	0.00	0.00
4,000.00	1,157.10	0.00	0.00
0.00	0.00	0.00	0.00
0.00	2,684.20	0.00	0.00
0.00	0.00	0.00	0.00
3,656.50	3,330.80	2,141.60	2,710.20
0.00	0.00	0.00	0.00
3,026.20	3,050.50	0.00	0.00
2,571.90	8,551.90	4,986.00	0.00
0.00	0.00	0.00	0.00
0.00	0.00	0.00	0.00
0.00	0.00	0.00	0.00
0.00	0.00	0.00	0.00
1,545.50	5,567.80	0.00	956.18
3,122.90	1,608.40	5,653.30	16,615.00
14,169.00	0.00	14,162.00	16,203.00
0.00	0.00	0.00	0.00
0.00	0.00	0.00	0.00
0.00	0.00	0.00	0.00
3,114.10	0.00	917.06	1,278.80
0.00	0.00	0.00	0.00
0.00	0.00	0.00	0.00
0.00	0.00	0.00	0.00
0.00	0.00	0.00	0.00
0.00	0.00	0.00	0.00
0.00	0.00	2,351.20	0.00
0.00	0.00	0.00	0.00
0.00	2,443.50	0.00	0.00
0.00	0.00	0.00	0.00
3,750.60	4,588.10	4,104.60	5,044.80
0.00	2,389.30	0.00	0.00
0.00	2,892.90	0.00	0.00

LFQ intensity MIC03	Intensity Cell01	Intensity Cell02	Intensity Cell03	Raw in
16,696.00	493,280.00	1,018,600.00	1,190,300.00	
31,443.00	178,310.00	316,710.00	355,740.00	
17,334.00	212,990.00	471,840.00	625,860.00	
16,774.00	70,654.00	312,020.00	305,690.00	
83,294.00	150,020.00	546,600.00	531,640.00	
42,188.00	128,680.00	353,480.00	490,550.00	
13,109.00	44,932.00	169,850.00	241,970.00	
5,825.90	37,224.00	116,020.00	201,850.00	
15,149.00	127,690.00	153,970.00	236,180.00	
8,487.20	177,090.00	301,160.00	532,450.00	
3,191.20	23,447.00	118,140.00	210,670.00	
5,406.50	198,750.00	266,240.00	381,350.00	
9,662.40	37,518.00	122,920.00	130,710.00	
18,281.00	27,267.00	53,172.00	86,258.00	
15,377.00	51,985.00	167,250.00	272,780.00	
375.91	289,310.00	286,250.00	291,960.00	
16,486.00	58,853.00	74,312.00	169,450.00	
10,754.00	50,419.00	99,903.00	128,640.00	
12,623.00	16,365.00	72,078.00	94,643.00	
11,327.00	8,597.80	271,260.00	328,570.00	
14,648.00	44,399.00	70,860.00	109,690.00	
48,641.00	224,890.00	443,620.00	331,410.00	
9,296.70	23,668.00	100,660.00	115,280.00	
11,689.00	16,295.00	58,113.00	82,202.00	
10,955.00	51,923.00	65,590.00	148,530.00	
17,676.00	28,817.00	68,371.00	90,706.00	
6,502.40	42,194.00	97,477.00	216,420.00	
10,865.00	57,192.00	137,790.00	180,190.00	
3,252.50	41,474.00	57,705.00	91,503.00	
7,975.70	8,114.20	97,717.00	103,450.00	
16,417.00	72,323.00	123,110.00	185,410.00	
13,670.00	25,170.00	41,277.00	81,763.00	
11,855.00	119,310.00	173,480.00	225,370.00	
1,445.80	28,247.00	76,546.00	90,470.00	
7,334.90	43,357.00	62,688.00	150,100.00	
0.00	0.00	23,181.00	37,515.00	
823.78	82,795.00	43,812.00	135,430.00	
1,798.90	38,354.00	43,683.00	104,250.00	
4,860.20	15,565.00	47,854.00	77,867.00	
2,879.80	15,856.00	30,641.00	99,514.00	
13,433.00	51,394.00	90,989.00	131,940.00	
9,319.00	2,081.70	109,510.00	167,330.00	
8,389.50	32,684.00	51,406.00	69,000.00	
6,411.40	16,670.00	43,209.00	73,250.00	
6,925.50	4,508.40	31,206.00	70,314.00	

3,614.80	24,798.00	60,828.00	102,840.00
11,381.00	29,806.00	52,334.00	79,766.00
12,066.00	18,034.00	88,753.00	81,611.00
7,337.00	4,207.50	20,432.00	38,410.00
9,726.20	10,289.00	30,009.00	40,415.00
13,814.00	382,590.00	437,530.00	380,350.00
8,247.40	13,026.00	58,439.00	98,070.00
2,469.30	35,109.00	52,518.00	49,095.00
12,535.00	34,445.00	24,280.00	28,692.00
5,990.50	2,601.90	44,861.00	47,913.00
8,001.80	10,626.00	55,968.00	68,920.00
5,382.70	8,939.80	26,479.00	72,859.00
0.00	19,452.00	75,061.00	105,560.00
6,649.00	14,970.00	42,222.00	57,797.00
16,230.00	18,300.00	72,698.00	72,070.00
3,764.10	3,044.40	18,520.00	55,834.00
3,002.70	4,833.00	7,699.40	16,612.00
4,187.90	2,348.60	37,424.00	43,722.00
2,717.80	0.00	37,514.00	45,861.00
6,092.80	10,091.00	19,006.00	33,026.00
3,911.60	13,113.00	40,453.00	82,678.00
3,939.80	19,927.00	22,724.00	46,257.00
0.00	31,247.00	82,276.00	82,077.00
1,555.20	12,412.00	26,840.00	50,731.00
5,729.30	0.00	10,554.00	0.00
2,749.90	1,143.40	30,938.00	39,230.00
0.00	33,637.00	26,158.00	31,406.00
6,981.30	18,710.00	23,084.00	42,800.00
2,063.70	24,895.00	36,590.00	72,255.00
2,471.40	0.00	11,155.00	16,324.00
11,219.00	5,764.50	16,283.00	7,634.50
0.00	3,518.30	24,605.00	33,290.00
44,212.00	76,091.00	0.00	2,099.50
3,986.80	4,605.40	23,074.00	37,963.00
0.00	0.00	0.00	5,733.40
0.00	0.00	5,745.20	11,605.00
2,129.60	2,958.00	3,378.30	15,168.00
0.00	0.00	27,499.00	24,542.00
3,325.30	8,110.90	27,982.00	35,734.00
2,855.50	2,862.40	15,853.00	29,977.00
5,958.40	3,907.80	17,198.00	18,663.00
0.00	0.00	20,488.00	16,261.00
1,743.40	5,904.00	11,353.00	25,166.00
2,292.30	1,509.90	22,029.00	36,500.00
0.00	2,135.60	16,365.00	9,458.20
0.00	9,214.80	10,325.00	9,552.70
1,402.80	6,294.00	9,578.00	18,135.00

0.00	3,992.50	18,025.00	40,015.00
14,771.00	8,491.20	16,424.00	11,155.00
0.00	20,466.00	28,689.00	46,702.00
0.00	4,446.30	11,798.00	17,374.00
0.00	14,010.00	12,975.00	41,585.00
0.00	3,363.70	6,312.50	0.00
9,135.40	0.00	18,062.00	27,286.00
8,962.80	4,596.80	11,775.00	21,775.00
0.00	5,197.60	13,952.00	30,465.00
5,611.10	4,592.70	16,543.00	9,148.00
6,398.10	8,346.80	24,201.00	0.00
0.00	1,456.80	16,897.00	18,102.00
8,340.80	4,935.50	19,766.00	20,818.00
0.00	3,555.20	17,308.00	24,193.00
2,586.80	4,914.40	0.00	22,736.00
0.00	6,508.30	19,854.00	28,925.00
0.00	14,881.00	35,266.00	48,345.00
5,060.10	13,324.00	12,496.00	37,023.00
0.00	6,071.30	4,412.40	14,870.00
3,289.90	13,050.00	15,580.00	49,052.00
5,047.90	6,830.40	4,567.30	18,090.00
0.00	2,481.60	14,529.00	0.00
1,350.80	0.00	12,284.00	23,712.00
2,736.50	0.00	9,406.90	26,768.00
9,563.10	5,289.90	13,564.00	21,596.00
0.00	0.00	0.00	0.00
4,142.50	3,478.00	14,119.00	17,700.00
4,091.50	5,887.40	2,798.40	24,961.00
2,624.60	0.00	0.00	11,117.00
3,398.80	1,954.40	18,030.00	20,939.00
6,418.80	10,647.00	9,711.90	20,034.00
1,793.00	0.00	13,921.00	29,627.00
0.00	0.00	6,563.70	23,718.00
0.00	9,680.10	13,595.00	22,984.00
0.00	0.00	9,467.20	8,929.90
4,891.50	0.00	10,495.00	19,910.00
0.00	0.00	0.00	15,676.00
2,343.40	9,946.00	10,289.00	30,321.00
0.00	0.00	8,005.30	13,545.00
0.00	2,903.70	7,164.80	5,815.80
0.00	0.00	0.00	0.00
3,339.30	2,949.10	7,704.00	17,845.00
1,933.50	857.39	0.00	0.00
3,158.00	12,328.00	4,618.10	10,406.00
0.00	0.00	9,093.70	11,563.00
0.00	1,998.60	6,378.90	14,527.00
0.00	0.00	0.00	9,149.30

0.00	0.00	11,874.00	8,340.10
1,578.90	0.00	8,659.80	2,553.40
6,426.70	1,468.00	0.00	8,656.20
2,854.30	1,710.80	2,862.50	15,548.00
3,577.00	930.97	14,154.00	12,228.00
216,910.00	610,920.00	802,830.00	868,140.00
0.00	0.00	6,868.40	5,483.10
0.00	1,299.30	6,728.00	7,138.90
0.00	0.00	0.00	0.00
0.00	0.00	12,259.00	7,786.40
0.00	0.00	4,564.10	9,511.30
0.00	0.00	7,395.60	4,081.10
2,577.60	0.00	0.00	0.00
0.00	0.00	0.00	0.00
0.00	20,111.00	0.00	0.00
0.00	1,899.20	6,999.40	6,114.30
0.00	0.00	0.00	0.00
0.00	0.00	0.00	0.00
1,415.80	4,063.00	8,882.00	9,356.90
0.00	0.00	5,703.10	9,687.80
1,631.10	2,182.50	24,810.00	14,694.00
0.00	0.00	0.00	8,318.60
0.00	7,677.10	1,865.60	0.00
0.00	0.00	0.00	0.00
0.00	21,377.00	8,448.20	14,051.00
0.00	0.00	0.00	0.00
4,724.60	2,657.40	2,078.80	5,025.60
0.00	0.00	8,561.80	5,430.30
2,962.60	5,486.10	4,523.20	11,510.00
0.00	0.00	14,677.00	0.00
5,692.00	29,974.00	29,690.00	47,277.00
0.00	0.00	0.00	0.00
0.00	3,569.40	2,371.00	0.00
0.00	0.00	5,748.30	14,234.00
4,544.50	5,914.10	0.00	9,768.40
0.00	0.00	3,195.70	7,739.60
0.00	0.00	0.00	8,375.30
0.00	0.00	0.00	1,204.40
0.00	0.00	0.00	0.00
0.00	0.00	0.00	0.00
0.00	0.00	0.00	47,277.00
70,411.00	0.00	6,521.10	3,918.90
0.00	0.00	0.00	0.00
0.00	0.00	0.00	0.00
6,676.40	4,545.70	15,439.00	0.00
0.00	0.00	0.00	6,000.40
0.00	0.00	0.00	20,675.00

8,618.70	2,662.00	0.00	25,315.00
0.00	1,854.20	4,738.00	7,289.00
0.00	2,463.10	3,282.30	8,438.50
1,356.30	0.00	6,771.50	3,215.70
0.00	0.00	0.00	3,749.50
0.00	0.00	0.00	2,005.80
0.00	0.00	8,331.40	3,457.60
0.00	0.00	0.00	0.00
5,385.10	0.00	0.00	8,020.80
0.00	0.00	20,351.00	0.00
0.00	0.00	7,053.60	12,170.00
0.00	0.00	0.00	7,669.30
0.00	4,552.00	5,301.90	9,042.40
0.00	1,446.90	4,417.80	20,006.00
0.00	0.00	5,266.80	12,635.00
0.00	21,853.00	0.00	0.00
0.00	0.00	0.00	10,909.00
0.00	0.00	12,193.00	13,209.00
0.00	2,383.20	6,850.10	18,093.00
8,394.90	8,838.00	15,681.00	5,026.00
0.00	9,837.20	28,905.00	42,153.00
0.00	0.00	0.00	14,595.00
0.00	0.00	0.00	0.00
0.00	0.00	0.00	7,779.60
0.00	0.00	5,965.70	0.00
0.00	0.00	0.00	0.00
0.00	0.00	0.00	0.00
0.00	0.00	0.00	20,942.00
0.00	12,754.00	0.00	0.00
0.00	0.00	0.00	8,018.60
4,464.00	2,458.20	0.00	0.00
0.00	0.00	0.00	4,800.40
0.00	4,414.20	0.00	7,301.70
0.00	0.00	0.00	8,375.30
0.00	8,616.50	5,785.30	12,993.00
0.00	8,088.80	0.00	7,139.50
2,773.40	0.00	0.00	8,644.50

tensity

Intensity MIC01	Intensity MIC02	Intensity MIC03	MS/MS count	Species
275,610.00	213,380.00	257,400.00	415	
335,020.00	282,700.00	385,170.00	254	
199,720.00	136,680.00	200,100.00	281	
312,950.00	348,740.00	209,100.00	244	
623,000.00	522,000.00	565,720.00	308	
290,960.00	315,470.00	247,320.00	250	
282,940.00	207,170.00	159,820.00	180	
177,590.00	123,940.00	40,744.00	102	
54,120.00	32,378.00	75,379.00	97	
104,260.00	97,983.00	67,446.00	153	
40,890.00	28,912.00	14,240.00	68	
64,768.00	20,274.00	34,038.00	123	
70,028.00	95,754.00	96,701.00	101	
111,210.00	104,170.00	54,795.00	78	
101,140.00	74,935.00	102,750.00	109	
2,389.10	6,042.00	8,803.30	103	
129,600.00	97,477.00	156,590.00	88	
174,490.00	154,350.00	46,522.00	90	
113,330.00	106,580.00	64,539.00	71	
333,990.00	259,520.00	69,910.00	129	
113,710.00	18,214.00	125,340.00	66	
344,260.00	217,510.00	349,080.00	229	
61,816.00	37,957.00	35,053.00	64	
84,878.00	62,896.00	45,069.00	61	
94,094.00	54,282.00	75,764.00	83	
118,810.00	97,148.00	80,873.00	77	
46,778.00	24,989.00	19,713.00	62	
79,046.00	77,336.00	54,107.00	69	
24,621.00	33,842.00	17,714.00	26	
85,613.00	105,270.00	34,466.00	69	
112,220.00	72,145.00	97,046.00	109	
62,570.00	48,887.00	54,814.00	55	
59,284.00	49,693.00	87,125.00	91	
16,415.00	0.00	3,323.70	35	
45,226.00	14,927.00	42,766.00	56	
0.00	2,364.80	0.00	9	
3,113.20	1,587.80	8,566.20	42	
6,499.80	2,437.50	3,028.50	34	
71,297.00	73,972.00	25,196.00	53	
3,315.10	8,528.40	8,102.80	20	
157,040.00	132,540.00	75,260.00	91	
116,730.00	57,272.00	32,953.00	63	
41,074.00	9,504.20	47,162.00	36	
45,931.00	35,327.00	24,847.00	36	
76,650.00	97,904.00	32,203.00	44	

21,306.00	30,335.00	10,286.00	33
13,999.00	34,430.00	45,788.00	34
42,933.00	29,271.00	44,538.00	42
45,542.00	36,766.00	32,647.00	33
47,718.00	50,305.00	37,168.00	31
331,180.00	438,840.00	374,580.00	57
16,362.00	13,924.00	25,433.00	29
0.00	0.00	5,737.60	33
33,937.00	33,159.00	55,228.00	32
39,368.00	67,719.00	21,601.00	23
38,223.00	30,340.00	20,378.00	25
27,099.00	40,474.00	20,588.00	29
16,760.00	12,476.00	0.00	30
21,071.00	13,188.00	26,078.00	28
55,940.00	51,971.00	52,004.00	48
10,986.00	11,425.00	8,795.10	10
34,072.00	24,483.00	12,360.00	15
28,143.00	33,375.00	15,567.00	28
60,224.00	34,684.00	5,105.60	28
16,916.00	21,317.00	21,844.00	21
32,940.00	35,587.00	15,325.00	26
18,496.00	4,746.30	12,255.00	15
0.00	7,004.00	0.00	24
3,486.80	0.00	1,519.30	12
8,997.30	7,394.90	18,286.00	15
7,610.00	15,624.00	9,995.80	19
1,113.20	0.00	0.00	15
5,719.90	4,953.80	8,553.50	17
1,202.70	0.00	8,202.20	18
32,234.00	13,863.00	8,006.30	11
7,338.40	7,285.20	33,952.00	7
0.00	2,898.50	0.00	5
0.00	0.00	134,770.00	22
20,621.00	13,943.00	9,276.50	8
0.00	0.00	0.00	4
0.00	0.00	0.00	9
0.00	0.00	2,604.30	3
21,905.00	9,754.50	0.00	8
2,406.90	0.00	6,330.70	18
1,087.40	0.00	3,689.90	13
37,556.00	30,995.00	23,634.00	14
0.00	0.00	0.00	7
1,112.20	0.00	1,163.60	12
5,675.00	0.00	2,809.10	12
10,072.00	33,046.00	0.00	14
9,324.00	4,166.20	0.00	6
2,160.30	0.00	1,912.60	6

0.00	0.00	0.00	11
15,665.00	6,108.80	41,513.00	9
0.00	0.00	0.00	13
3,062.50	5,983.00	0.00	5
1,222.10	0.00	0.00	11
0.00	3,007.00	2,655.30	0
30,277.00	5,374.80	33,918.00	12
14,851.00	3,641.10	15,777.00	11
0.00	2,768.70	0.00	8
25,346.00	20,773.00	10,909.00	17
15,681.00	11,348.00	5,915.60	8
16,418.00	3,488.10	0.00	10
26,212.00	11,313.00	15,320.00	11
0.00	0.00	0.00	13
10,583.00	3,233.70	3,058.00	5
4,699.70	0.00	0.00	13
0.00	0.00	0.00	19
7,350.50	5,420.10	10,338.00	11
0.00	0.00	0.00	4
12,435.00	11,435.00	12,320.00	11
19,305.00	5,916.60	15,915.00	5
0.00	0.00	0.00	4
0.00	2,247.60	1,980.50	5
0.00	0.00	3,981.90	5
11,679.00	8,098.10	19,826.00	18
0.00	0.00	0.00	5
1,450.90	0.00	5,948.10	8
0.00	0.00	7,787.30	4
6,758.70	6,930.80	3,278.60	9
17,656.00	0.00	2,988.80	4
10,737.00	10,001.00	23,584.00	10
0.00	0.00	1,728.20	4
0.00	2,387.40	0.00	5
0.00	1,200.70	0.00	10
5,260.40	0.00	0.00	4
14,049.00	3,169.60	6,770.30	8
8,444.60	7,997.20	0.00	4
12,918.00	6,526.00	6,224.90	11
0.00	0.00	0.00	4
0.00	1,783.20	0.00	2
0.00	0.00	11,870.00	2
6,661.30	8,056.20	4,259.30	4
5,785.50	0.00	3,264.30	2
0.00	0.00	4,746.50	5
4,962.90	2,640.30	0.00	3
0.00	2,227.10	0.00	3
0.00	0.00	0.00	3

0.00	0.00	0.00	4
5,922.60	0.00	1,831.80	3
0.00	1,592.20	8,028.30	4
7,179.50	0.00	6,998.10	4
18,046.00	17,198.00	12,557.00	8
697,010.00	686,560.00	589,160.00	128
1,630.80	0.00	0.00	1
4,620.10	0.00	0.00	1
0.00	0.00	0.00	2
2,746.50	1,671.60	0.00	2
0.00	0.00	0.00	2
0.00	1,482.30	0.00	3
7,057.00	8,156.80	2,871.30	2
3,102.20	8,165.00	0.00	1
0.00	0.00	0.00	1
0.00	0.00	0.00	1
0.00	0.00	0.00	1
0.00	0.00	0.00	2
0.00	0.00	1,456.10	2
7,528.30	0.00	0.00	1
0.00	0.00	2,266.10	8
0.00	0.00	9,612.70	1
0.00	0.00	0.00	3
0.00	0.00	0.00	1
0.00	0.00	0.00	7
7,306.80	4,744.60	0.00	2
8,850.10	9,503.70	5,531.60	3
1,287.60	0.00	0.00	2
9,645.10	4,710.40	2,710.40	3
0.00	0.00	0.00	1
0.00	0.00	7,110.50	10
6,289.50	5,133.40	0.00	2
4,055.50	8,068.90	0.00	1
1,679.60	0.00	0.00	1
0.00	0.00	5,017.90	1
0.00	0.00	0.00	1
2,219.70	0.00	0.00	2
0.00	0.00	0.00	2
6,688.70	5,631.30	0.00	1
0.00	0.00	0.00	1
0.00	0.00	0.00	1
89,078.00	27,327.00	125,630.00	3
0.00	0.00	0.00	2
0.00	0.00	0.00	1
0.00	2,422.00	7,552.20	2
0.00	0.00	0.00	1
9,606.80	0.00	0.00	2

9,450.20	0.00	9,901.50	2
2,685.10	0.00	0.00	1
0.00	0.00	0.00	1
14,023.00	12,334.00	2,256.60	4
2,407.80	0.00	0.00	1
0.00	1,277.80	0.00	2
0.00	0.00	0.00	1
0.00	0.00	0.00	1
0.00	0.00	6,727.10	1
0.00	0.00	0.00	1
2,815.70	3,665.20	0.00	1
0.00	0.00	0.00	2
0.00	0.00	0.00	2
13,330.00	0.00	0.00	1
0.00	0.00	0.00	1
0.00	0.00	0.00	1
0.00	0.00	0.00	2
0.00	0.00	0.00	0
0.00	1,018.50	0.00	2
21,894.00	75,115.00	23,536.00	6
23,634.00	25,149.00	0.00	2
0.00	0.00	0.00	1
0.00	0.00	0.00	1
0.00	0.00	0.00	1
1,389.10	2,195.60	0.00	2
0.00	0.00	0.00	1
0.00	0.00	0.00	1
0.00	0.00	0.00	1
0.00	0.00	0.00	1
0.00	0.00	0.00	1
4,105.70	0.00	5,315.00	1
0.00	0.00	0.00	1
0.00	0.00	0.00	1
0.00	0.00	0.00	1
5,563.30	6,830.80	0.00	1
0.00	0.00	0.00	1
0.00	0.00	3,464.60	1

**Peptide sequences**	**Only iden**
AIELNQIWDFPIK;AIELNQIWDFPIKEFHPFPR;AISEHIQDDWCTPGNPR;ALLGVGAHDIIGVEAK;CDSIISVG	
AADLLSGPWR;AADLLSGPWREK;DFVLAHSSAHPDVLR;EVGANIDR;GAFDYQGQK;IAAATMLGQSK;IDVV	
AEIENSDSDYDREK;AGAATEVELK;AGAATEVELKER;DETTIVEGAGDAEAIQGR;DLLPLLEK;EGLRNVAAGA	
AGQWVADQYASATGPVNIVQLEGTTGADPAIDRK;APGSEGGSAPDGALTLGFAQVGAESGWR;AVDTQDTD	
ASVNIKPLEDK;ASVNIKPLEDKILVQANEAETTTASGLVIPDTAK;DVLAVVSK;EKPQEGTVVAVGPGR;EKPQE	
AAALNIVPTSTGAAK;AIGLVLPELK;FDSILGR;GKLDGYALRVPIPTGSVTDLTAELAK;LADLVALVGK;LPQDVS	
ALEGTDISAIK;DAGISVSDIDHVVLVGGSTR;EAGQIAGLNVLR;ELTGGKEPNKGVNPDEVVAVGAALQAGVLK;	
DATDPLELK;DLFEYQAK;EDATLVEVNPLVR;GVDLAFAR;HNVPTSPGR;ILAEANHPLVIQAETMDAGADKAA	
AANALTDLGLVAGDR;AASLKEAVDEAVADQPSVK;EAVDEAVADQPSVK;EIHIVPELPK;EIHIVPELPKTR;ELG	
ALDAAHAAAPAWGK;AQLGGDLNGGYYVAPTIFTGNNK;ETLNADIPLAIDHFR;IAFTGETTTGR;ILSYIEIGK;	
AFLAELNPGFASFVEDKIEQPIGR;ALLEATAFGTR;GLQILEEDPEIYAAIDR;HGVVTEALPGHPEVR;INALAAA	
AFDQIDNAPEER;EHVLLAR;ELLAAQDFDEEAPVVR;GITINISHVEYQTDKR;GQVVVKPGTTTPHTEFEGQVY	
AGVVPPAEDDDPAEWK;GCAESLAGQGAR;GVTEETTTGVLR;HSLIDGINR;HSLIDGINRGTDVLIGGKK;IHV	
AEEAINSGFFER;AGEGHAFISAGVETVSR;AVAVQLGYDFLPGTTVNR;AVLDKVPALDPR;EITPVTLPDGTVVS	
AAVDSGYYPGQFQVGQTGK;AEVLVLVEHADGALKK;AEVLVLVEHADGALKKVSAELLTAAR;ALGEPSAVVVG	
AELIDVLTTK;AVHKGDSVTITGFGVFEQR;AVISGAQKLPADGPAVK;AVISGAQKLPADGPAVKR;GDSVTITGF	
AASGAVLSAVGPK;AIPNLSVVRPGDPNETAYAWK;EFGFTPEAVAAAAER;ILGFDPEKNFEVR;KVTDKPSFIAL	
DYSTENASGGTSGPFYDGAVFHR;HTIFGEVVDEESQK;HTIFGEVVDEESQKVVDAIASTPTDR;IALFGNHAPK	
AVENFPISFR;CIDGLVANEER;DGLVEASGALR;ELAESSPSIVTPLNSAIGYEEAAK;GLIGDKLSLEELDR;HLIPA	
IPDEDLAGLR;LEEENPEAAAALR;TVGDVVAYIQK;TVGDVVAYIQKLEEENPEAAAALR;YGVKIPDEDLAGLR	
AAAESAGLPLFR;AALDLISSAIEATGLK;AAVPSGASTGEHEAVELR;AVEAVLDEIAPQVIGLSADDQR;GAANAL	
AKLEAAGASVTVK;AKLSTEELLDAFK;DLVDSAPKPLLEK;DLVDSAPKPLLEKVTK;EAADDAKAKLEAAGASVT	
AADLPPAVGVLVEYQAGDADKGK;ALLDEAGVTVTR;ANDIETLK;ATAEGLVAAK;DVVLLDQPSVSDNKK;EAA	
AAADVGAPAAVSR;ADLATLVTVEAGK;AIVFSAAGTAGQR;LGQLLVEHK;TANITTDK;TANITTDKPQGAGAA	
AVEEALLIK;AVHVKDDGLHGSDLIQTAWTLAR;DDGLHGSDLIQTAWTLAR;EAADAVLDEINER;EGGDSTVT	
ALAEQEEAIVSELNSVQGK;AVNPESTFSR;FAEAAGIDVK;GYNVPDYPANPK;ILAEFGDHLTEEQR;ILGSAVNP	
EGNDLWVELADANVLK;FTQAGSEVSTLLGR;GIFPAVDPLASSSTILDPAIVGDEHYR;GVEVTDTGASISVPVG	
EATLIVEEGVATK;EDVDTIVR;GWTGDFDDETK;GYHTLENAFGER;LLTDNVDAGR;LLTDNVDAGRYGTK;LQ	
APLVPGTPDPVKDADEVVAFAK;AVVSDPAFIGDGEK;AVVSDPAFIGDGEKFDVHTR;DAGLASVAVYAEPDAD	
GFGFIAPEDGSADVFVHYTEIQGSGFR;GPQATGVR;TLEENQK;TLEENQKVEFEVGQSPK;TLEENQKVEFEVG	
AALGAAELR;ALAERLDASADLPVAVR;AVYDVSDPEIDK;AVYDVSDPEIDKLVQEYEDTYAVAEELRR;EFGYIQ	
LIEENADLR;LKTYLESQLEELGQR;PLTPADVHNVAFSKPPIGK;QTAETTVSEAR;TYLESQLEELGQR;VAELDQ	
ALLEKFDQS;ALPQLTDEQR;ALPQLTDEQRAAALEK;GGTNLKQVLTDAETDEVLGK;QVLTDAETDEVLGK;VS	
AWGTIEELK;GGLILDIGLR;HDVDPNEVVSVGDEVEALVLTK;IIELDKNRNNVVLSR;LVPFGAFVR;SEESSGGT	
ALELQAPSVVQR;EAWLTGDPK;FESELLEHVK;HVLIVFDDLSK;LDLSQYR;QSVSEPLQTGIK;TAVCVDTILNQ	
ANYGPTIAAYR;TAALDEAIALLGDR;TLLGDGIEDFETQRR	
ALDNVKPALEVR;IVYGALEQAR;LANEILDASNGLGASVK;RPLVNDPVYGSQLVTQLVNK;VGGATYQVPVEVR	
ATVGEVGNAEQSNINWGK;GASVSDFAEITR;NIPAGTVIHAVELRPGGGAK;QGDVIESGANADIKPGNNLPLR;	
ALKIVIGGDNAGFNYK;ALLICGTGLGVAIAANK;EWLGLEFDPQSASAAK;IVIGGDNAGFNYK;VASVEDVGVG	
AFDTQAGPAITSAAR;GLVAANPDLLTEVPGGVVR;GVAGDLPVFK;HEGIDVR;IVTVSDDIASNSPENHR;SFGL	
AGTQIVGGVNAR;ANVTKPVVGYVAGFTAPEGK;DKDGNDIELPVFGSVAEAIK;EALEAAGVK;ETGADVSIAFV	
AEVPGIDPAK;AEVPGIDPAKDIDITVR;AEVPGIDPAKDIDITVRDGVLTIK;GILTISVPVTEPEAAAEK;GILTISV	
ALCAAQQELADR;ALSAEVAVIPR;ALVPVLPSIEEFPYAIR;IALIDGTWVAFPTVEQLER;IGEVIGPK;INAIANPQ	
ALGGGVVPLSAVVADR;ELSVALAER;FADTLAEVR;GFGPYTPGFR;GLWAGVDIDPALGTGK;GVAAGQSNIVV	
AVIDSAAAAFPAWR;EAGLPDGVFNVLQGDK;EAGLPDGVFNVLQGDKTAVDELLTNPK;GLEVVEFACGIPHLL	

AASSGATVLCVSK;AVVVIGADGNVVYSELVPEIGQEPDYEAALK;FCGAEGIENVTTASAFR;GNPINTVGELPAV
IITSPSFGGDR;LLAQNTGVIASFSR;NLDSLADDQK;SVIGAANPTGVAAVVAQQFEVAK;TIEGKPSATYLWEDK
AAGLDVIGADIAPYQDK;EIDPAANEDVR;HEEFFSNLVSYVLDK;HRDETVEAIAAR;IATDEER;ITAWGLAGEA
ALHSIGAPLEPGQK;LCAHLVEAGTFQK;LGDDGFFVK;LNPEKQPNSYLALSDPSDVAR;LPTGLKDEFDALK;TIA
DGQPLFHDESGYAGLSDIAR;GGYFPVAPYDHYVDLR;HYIGGILHHAPSLLAFTNPTVNSYKR;IEPLQPVDK;IPI
GTLTLDVAEPISVSR;KVGKTGK;QPVPVPSGVDVTINGQNLSVK;SRIGKQPVPVPSGVDVTINGQNLSVK;TLIA
DIQDGSFVK;EKAEEQGLEVDTPAEVAK;GPGHLVR;GVPCLIAIDQDPK;GVPCLIAIDQDPKGEGQALALSYAAA
AAGVETVVFDR;AAGVETVVFDRGGYTYGGR;IAALADAAR;VAGTAEVPR;VGQLIAER
AITTLDVVSR;HFDHLNVIAGFDELAR;SVVGTPEQIAEQVK;TENALANPR;VQLGTLVTGNTYR;VQLGTLVTGN
AAGFDVTVPFTPGR;DWWPNQLNLK;ILQKNPDVINPLDEDFDYR;NPDVINPLDEDFDYR;SAVQNLDVDALR;
IPVISYDR;LLVIAPIDGSSLTNTLQHAADLK;SAGYGNAAKPLPIVTGQDAELASVK;SIVAGEQTQTVFKDTR;VD
FFDGFPR;LGGDDELLDIAK;LPTIAAYAYK;QVELSTIR;SAITYIDGDAGILR;SQGGDVSDFVR;YPGGELDLEIV
EGVHTVGELVAR;ELNADSEHIEIGPSPAEADHIASFALPIDDLDLTVR;FVIEPLEPGFGYTLGNSLRR;IDGVLHE
ACDPVELEELR;ACNDPVADQLLQR;AVVIAVGGVYDPR;DYLVVTR;LVEELEDACDKFEVAK
AEAEQFTEFR;ALIGADPVLDER;DLAVSGPWYR;LDELGIAHGGIVDAPYGSGLSFR;VGLDHVSFGVGSR
AGIPLVVLGGK;AGLWPYLEK;AVITESFER;GVAEVPLANR;LNAVPGTEKLPYSLK;TDEQLALVEAYAK;VLAENL
IFFTNGGADAVEHAVR;VGAEVIGPGLAELAAK;VVAAIAEQAAK
AEGLTDGDVLLLENIR;GVAEAIIGATGK;LAVIENLATK;LSLAPVAAALGEK;TLDDLLAEGVQGR;VIASVPTLQA
AELDQLIANRPVIAAEINDR;QEGISDGKLEVYSPNSPLGGALLDAK;QLQELLNNAK;QSGVALPGSVVK;TDTQV
AALVTVDTYR;IADIADPLPR;IVLDLTDGQGKPSER;LAVGIYDDVDGK;TEVPELVGVSR
AIGEVFDLRPAAIVR;DLDLLRPIYAPTAAYGHFGR;IIVDTYGGWAR;ILDIGYDSSTK;LLVNPTGK;NVVAAGLAI
AAGLFNAPASQVAR;ALGDSATYTVAK;ALSVADVEK;ASTATVVTEYR;ATAVADIAEQFK;GLTVANLAELR;KA
ALILVQPEDRPALK;ALILVQPEDRPALKK;APLVTVDR;APLVTVDRVDQFDIYAHLDGGGPSGQAGALR;EPVIID
LAENYGAALDEVLR;LYTPLEAAK;TGTVTPDVAK;VAVFAVGEK;VTVSTTTGPGIPVDPSVTR
FLLDQPTTSAGR;LVFLTGPK;LVFLTGPKSDDSGSNA
IGSDEALALR;LAAIGIDLPDVFR;LQTGNLTELINTR;SVVGVTTNPSIFQAALSK;SWQELLDATQGQLDAAKK;T
AGPNVVTR;KVIKPVAGQFAAAGVNPR;NGGLVVVR;VDAENGVLLIK;VHKVDAENGVLLIK;VIKPVAGQFAAAG
HLDDATIAK;IATTVDPNSSTTALAGSTEEGQFH;LAADVADVPTVVIAR
ETAGERPIPSWLQVVGER;ILVHQLPER;LGVDLVGGDQSFEK;SLNTLPFQIAR;VSQYDIIDVK
EGGIEGIEEYLDTK;GLDPSSTLGPLINSK;GVISDPAAPFGGIK;GVTGALIDDGR;QVATVTELVSDAVSR
AALAWSVETAK;IDILAEYER;THVAPAVPGAPA;TVADPGSLGIVLGGSGNGEQIAANKVPGAR
DFTFVCPTEIAAFGK;ELTAACGVLNADGVADR;VLDALQSDELCACNWK;VLGVSVDNEFVHFQWR
ANGDHAAIFLNEK;NLAFHLGGHINHSIWWK;TSGLIFG
SEIPADLYYTSEHEWVLR;VEAGTLDEALGGLLDAEGYR;VVAVNGDLEGSPELVNSDPYGEGWLVDLR
AVAAALQAGYR;TPAQVLIR
DLTDDQVTVLR;DYIEGNLK;LVGVDLPR;SNEILAATGIDK;TKDLTDDQVTVLR
ALPTGILDALDAAGR;LVGVSVDSDGSPAYR
QPLVDLK;VADYLAANPQVK;VADYLAANPQVKDELTAVR
AEPATVSELDR;QLNLNESVLR;TVAPSLETFLNVIR;TVAPSLETFLNVIRK
IHAVTELVEGQTPSTK;TFLGTLTENK;VLVVIGR
DGGYNVTVPFAPGR;STVLDSGLSTGQLIK;TASTSESENPAIPSPKPK;THGAGDASLVGPEPEAAPIEQQGLGW
AQYWLGVGAQPTEPVLALLK;EEPSLIQIDSER;IIVADAR;LDLFNAALAEAESGTTAAATTPK
GDTVLVISGK;IDDETGK;VLVAYPDR;VLVEGVNR
LGLVGLINTLAQEGAK;TNAIAPIAATR;VVVVTGAGGGLGR
LVVELTPDEAAALGDELK;TGDGPLEATK
GIGATIAEVFAR;VDILVNNAGITR;VVVVGTTPAEAGSVHAQVVQR
AALPEDVNAPSGEAA;EQQLHSLTYAYR;NLAELAVSDPAAFTALVDVAR

DIILAPVISEK;SYGLIEDNVYTFVVHPDSNK
FTVEPNSPIVVPR;RVHLVADLR;SASPEIVAAAR;SATAFLADFHHVATFGATENQGVDR;SSLLADIAK;VAADL[illegible]
ALGFPFKEN;LINGAINDLALITGQKPEVR;LISIALPR
IIFLGSQVDDDIANR;LNAEFTGQPIER
LASAAATLLR;SWYVIDASDVVLGR;TIGELLEK
QLGTPDVIPPADVR
TYVIAEPCVDVK;TYVIAEPCVDVKDK;VGQTDNDPQAIK
ILLLDGLPNYQR
AEPAPAASSDGSPYVTPLVR;EGVNLTFLPFIAR;LAAENGIDLSTVK
FALNAANAR;VEDVLGLPANTLK;VVFINTGFLDR
ANTTAESLAGLKPAFR;DFSGTDLGAIAIR;TTSVIVAGAR
AGELINAIADGSIK;ITVGGTYPLAEASR
GLGSAWTTLHLLGEGEK;LPAEQFAHWDTW;QAAEVVGIPFDK;TLNLSVDELLTTTR
LVEILEK;TAIGYVVSDK;TIVVELEDRK
AGVLFNDLVASGK;VSAPIVIGR;YLDEVADNLDDAVTK
AIAQVATVSSR;EAVEDAVAAAK;EVGLEVLGSAR;LAGGVAVIK;LAKLAGGVAVIK
STVTEFPSATAK;SVEEALDILR;WAPQAASEPVAK
VIASAVAEGLQAR;VPSAVWVVDTNKEHIAVGEAR
SLVVQLK;TMTDPIADFLTR;VLGGLGVAIISTSSGLLTDR
ELWSGPATFVFTR;IAVDGGFLSVTEETVR;TTAGEIGILPR
ALDLRAPLTPAPATAAVVK;APLTPAPATAAVVK;DNALVVLTPDASAAIAK;VGVGPLAPSDVLHVAR
EILGQYGLHDTDTGSPEAQVALLTK;YVAQVDVAR
GNVIPVEITVYEDR;KADLNANDIDAAAK
EAITAAGGSATEL;FRTEYEVVNVGDINK;TEYEVVNVGDINK
LVVIKPYEASQLR;VAIPQLTEER
LEDAIGSAAK;LVVVADGPATSRPLK
ATPSVIVALSGVR;LTTAEAANGIGVR
GLSEVLR;LINLTHGEPITFGADGEYAVVK;SGYGLEIAK;SLVPTADQPQKAKDFTSDQEVR
GALSLSTAQK;VVDAAIEK
GAVLEQVVNDALPSR;YSEAVSTSDLKPLGQPEIEITK
GRPHLCFDTFNATQK;IGLDGIEDAFHK;SVVVLDR
LFGSVTAADVVNAIK;LFGSVTAADVVNAIKK
LEALDLVPANR;NAAGQIVAAGAVAALDKLAEDPPAK
EVALLPFGSSTR
AVVSIDGGNDLSK
ELEDGLAPELR;SLTDLVEQPAK
TVNAVGALDEVFAR;VVLLGPPGAGK
AVPEQIRPWVPGTYVTLGTDGFGFSDTRPAAR;IVPIIPDEAR;LTTDQLDGFR
IWVYCGNGKPGELGGTDLPAK
LIDILDPTPK;TGASVVGPVPLPTEK
LVLQDDRNLVLYAK
ADYGPLVTEAALNR;FIDLVNQNADELAR
DTTLDVPTR;LVTADWLASNLGR
LGGSSSHQSALLANLATSLFEHGR
EDEVISRKPSVDEPFSALAFK;LLETVAESDEALLEK
EYGIIDTVLQYR;ILTAEEAK
AGIADVEIER;QVQLNILEVK

DGEALFLR;EAAENVAESLTR
AEAEAELQR;LLCVPAGDPR
DVKSGWK;VLLLPLGPLAEHNIR
ALVNSAGIGWAQR;VVIADLQADKGEALAK
GFGFIAPDGGAPDVFVHYSEISGSGFR;VEFEITQGAK
KVLIMVK;LPAALIQGLR
ASDVADLLQK
TVGLSPEAQQDLVQK
VLFELSYPDEK;VWINIFPDRPLTK
AAQLGLNTAIVEPK;WDLTVNELAR
LIGPGGEQVGIVR
AVGTTPGELR
IAVEDDPDSPHLLADK
DVDHDAVGLNPDLGNLVR
AVEHASIVTALELLR
IAYVEIGAADVR
AVEQWESLVR
VAVTAEVLEHTKGPK
AWIADRPEIAEQLLR
TTYTSLDGVFAAGDLVDHTYR
AFPQGGTVGVDELVAK;EAITAAGGSATEL
ALLPVLTGDKSPAAQSDTSTDALVRR;WLTDALGDAAVAK
PTIQQLVR
LVTTTDAFTAEDLLDGAK
EAVDAGDKDKASELLHATSR;SALALALNKL
DSPGLPKPVQTPHPPIIIGGQGAK;SNSPLVGTSTEIVDK
VVELDDEAAQR
IGVTLLEAVPYGLHAQAVK;LQQELSTLR
LPADVPVYGLER;SNLGHLESAAGAASLAKMTLALANDK
IIQQEFPR
LAVNGILK
VQFQGGGPHAVYLLDGLR
AELENAIK
FVAGETVPEALDATAALR;LSALGQALPR
GLINTVHHLVEVEEVGK
LLAEVLTAASR
LLTSGPIR
GERRAGPGSAR;HDRGSGR
TQPELASR
LGLAFAADPSQQLAR
IALILER
VAEVVLETLDDTK;VLIAEDEALIR
FLLAADGTVVNR
LILADAIVR
ILAATKALR;TGASDPLLLGQR
LADVLQGR
TDFILWIDAPGAAGDAR;TGEADVPVAVR

MLKALYK;VTVFSPSEWELSFPR
VEAPELANFGAK
TLGPFTWLK
LTDLLATTR
FAEAQKEAK
KIVDALR;KSGIRIVR
IPADVIDAFHAAT
EILASRMLIR
IAPTFPSLPDVR
DLPLLTLPPDVPFR
GESIEFPLR
NYQVVTPTAWNIGPR
LGEFAPTR
VQFVDPQDAVTPQGSR
EFTDGVLTALR;TLDAGLSTPDVGGSAGTR
LALTVDSLAERANPQR
VTVVGLPDVPGYAAK
AAGRRVR
VIADGHR
LDAAGVPWGR
AFAPYGLPATAFK;NIANVGDSVAYRYLDYSHGELQVAELTWR
AVVDGDLVGPFTLQATDR
VAAVGRRR
LQVEAPPDLFSGQPLSS
AVIATEDQLK
LAGEVAFHLVRCGR
SRSPELFADGATAR
PQTVEVTLGK
LAEALAVSWNTANNAVLAEGQR
FSTVIDSTLFHSLPVEGR
SGLIIAGLEVPHLHVHVFPAR
LIAEDGELK
LWLAVLR
LLGEVGLR
LESAHAVGPWFR
VGLVDQVLPR
LDLPENATPAYLGFNLFGHAIAR

Reverse	Potential	id	Peptide IDs	Mod. peptide IDs
GGSSHDAAK;DYNVME		167	65;66;70;97;160;198;213;222	65;66;70;97;160;198;213;222
QPHR;IIDVVDTGAK;LA		135	3;4;170;264;294;378;399;415	3;4;170;264;294;378;399;415
NPLGLKR;EIELEDPYEK		12	34;50;51;167;181;227;233;23	34;50;51;167;181;227;233;23
YK;AVDTQDTDVYKTF		57	59;112;130;131;269;456;457;	59;112;130;131;269;456;457;
GTVVAVGPGRWDEDGE		51	122;123;192;244;245;437;44	122;123;192;244;245;437;44
LEGDDTIVIGDTK;NTD		86	2;68;277;316;486;573;649;69	2;68;277;316;486;573;649;69
;GAASDGGGSKVPEETL		9	82;163;206;254;292;337;374;	82;163;206;254;292;337;374;
ELANK;LWEVFVK;MDLF		150	165;180;215;343;366;425;61	165;180;215;343;366;425;61
DTSTLVDPSVFEAIR;FA		163	19;22;211;235;236;248;273;3	19;22;211;235;236;248;273;3
MEENLESIALAESWDNG		227	79;118;262;382;433;620;643;	79;118;262;382;433;620;643;
R;LPADYALQVPADYIDV		59	48;95;324;363;439;570;628;7	48;95;324;363;439;570;628;7
LSK;GVINVNEEVEIVG		20	46;231;250;315;334;345;473;	46;231;250;315;334;345;473;
EALGGTLTK;INIKPQVD		60	62;298;350;369;370;414;440;	62;298;350;369;370;414;440;
K;EITPVTLPDGTVVSKI		144	32;52;129;145;239;240;249;4	32;52;129;145;239;240;249;4
KPGTAAGLTDGLK;ALGI		68	24;40;41;85;86;257;409;458;	24;40;41;85;86;257;409;458;
GVFEQR;LPADGPAVK;		70	37;138;142;143;299;568;624;	37;138;142;143;299;568;624;
R;LLAGKLPEGWDADLP		89	21;69;220;428;480;550;572;6	21;69;220;428;480;550;572;6
;SDRPTEPVVIESITIA;T		0	199;371;372;384;699;815;81	199;371;372;384;699;815;81
LEVLHASLAAK;LGELAI		141	135;161;173;246;320;365;52	135;161;173;246;320;365;52
		120	443;517;830;831;1009	443;517;830;831;1009
LVK;IEETLGDAAR;IQL		146	1;15;25;132;291;400;448;592	1;15;25;132;291;400;448;592
K;EIVSGLGLK;EIVSGL		27	73;74;184;185;201;241;242;2	73;74;184;185;201;241;242;2
HAVALQIAALK;EDVPEI		77	5;94;107;124;193;203;218;62	5;94;107;124;193;203;218;62
GLPTADELR;VALVSATC		210	0;28;72;530;744;745;858;911	0;28;72;530;744;745;858;911
VLTAGPER;IAEYLVAQK		73	133;139;166;200;225;381;47	133;139;166;200;225;381;47
VLR;SGEEIVLKPEVK;T		209	76;148;271;355;426;429;705;	76;148;271;355;426;429;705;
DGVK;IALFGGAGVGK;I		132	228;286;311;344;383;452;63	228;286;311;344;383;452;63
YALLK;VCASARPDTIIG		185	209;217;352;353;558;559;58	209;217;352;353;558;559;58
APHVR;FVAPTGPGVR;I		63	114;152;153;164;289;388;54	114;152;153;164;289;388;54
QSPKGPQATGVR;VEFI		183	306;333;795;796;797;878;87	306;333;795;796;797;878;87
TR;HFLADGFGAFTTNF		61	16;77;156;157;221;362;506;6	16;77;156;157;221;362;506;6
ELAAAR;VLSLAQDTAD		112	536;548;656;679;838;851;91	536;548;656;679;838;851;91
ALLEALPK		84	96;100;101;309;683;934	96;100;101;309;683;934
LASDAQLAALR;SEFLN		108	158;308;357;416;607;700;70	158;308;357;416;607;700;70
R;TGEVLSVPVGDAFLG		134	83;214;278;376;512;678;749;	83;214;278;376;512;678;749;
		205	111;741;801	111;741;801
PDR;WLVNFSR		21	81;466;496;688;884;991	81;466;496;688;884;991
SAGVSIQLLGK;TANIAI		29	127;296;638;666;691;743;10	127;296;638;666;691;743;10
VDDTTSYPNVAVAAAE		182	93;98;267;459;861;962	93;98;267;459;861;962
AFEGCTLPGATEPLFHV		181	47;327;342;359;464;703;839;	47;327;342;359;464;703;839;
PAFSK;GPIGLVSK;IIG		151	60;110;177;208;261;332;418;	60;110;177;208;261;332;418;
PVTEPEAAAEKR;SVTLI		109	42;43;44;313;314;734	42;43;44;313;314;734
LPK;ITTIKVPVDKIGEV		80	78;103;106;385;406;438;454;	78;103;106;385;406;438;454;
AR;LQDLIGR;NPEIVAT		23	88;252;270;307;328;339;576;	88;252;270;307;328;339;576;
;GVSFVGSTPIAK;KEEV		71	141;204;205;318;349;470;75	141;204;205;318;349;470;75

GSSAPGFTLTGTDLGE\	99	23;155;275;329;733	23;155;275;329;733
;TIEGKPSATYLWEDKC	98	421;551;641;732;783;784;95	421;551;641;732;783;784;95
SLK;VANVAEAGIFDK	142	9;232;358;367;390;451;859	9;232;358;367;390;451;859
AGNTVFTNVAK;TTVPI	201	89;504;522;566;574;780;824;	89;504;522;566;574;780;824;
TGNNPK;LIKDENVEYV	116	174;310;377;401;444;539;60	174;310;377;401;444;539;60
NLVTGVTEGYTQK;VG(	36	338;477;676;724;800;887	338;477;676;724;800;887
IGGAR;LIVDLIYEGGIA	72	176;243;331;347;348;546;59(	176;243;331;347;348;546;59(
	35	12;13;379;853;888	12;13;379;853;888
ITYRNPTLLAK	102	71;360;735;757;931;932	71;360;735;757;931;932
THGNGDASLVGPEPEA	172	8;195;432;645;698;778;871;9	8;195;432;645;698;778;871;9
AVLSPYDGISR;VVPSY	56	447;561;692;713;869;971	447;561;692;713;869;971
HATEGADGIALGSLLAK	152	280;525;575;681;693;723;10	280;525;575;681;693;723;10
FTTVPGVK;IPVDSIYSP	52	229;251;290;397;446;538;62	229;251;290;397;446;538;62
	154	26;27;151;197;596	26;27;151;197;596
	117	31;90;178;507;885	31;90;178;507;885
LR;VVPPGTGIVHQVN	90	55;56;144;341;565;753;902;9	55;56;144;341;565;753;902;9
	74	404;883;950	404;883;950
LAEAGAK	87	33;340;502;585;793;895	33;340;502;585;793;895
TWLTQEAFDR;TYTVP	145	35;665;672;677;756;840	35;665;672;677;756;840
	193	18;380;460;501;759	18;380;460;501;759
ER;VEVQVAYAIGK;VVI	85	67;179;423;427;562;651;881;	67;179;423;427;562;651;881;
GEEAA;LAAALQEK	19	10;84;104;121;125;326;469;4	10;84;104;121;125;326;469;4
RPIQTVGR;LVPGTGQ	54	91;92;115;116;258;608;798	91;92;115;116;258;608;798
	18	489;617;773;864;948	489;617;773;864;948
	101	283;598;599	283;598;599
ILQAIELWK	211	408;485;578;736;738;787	408;485;578;736;738;787
VNPR;VVPVTVVK	25	58;478;635;868;892;898;972	58;478;635;868;892;898;972
	13	364;391;483	364;391;483
	42	260;436;532;715;939	260;436;532;715;939
	148	226;317;346;351;680	226;317;346;351;680
	127	14;398;779;826	14;398;779;826
	217	169;253;903;906	169;253;903;906
	173	108;640;814	108;640;814
	106	702;873;953	702;873;953
	69	128;809	128;809
	43	183;196;603;719;789	183;196;603;719;789
	195	102;604	102;604
	5	675;849;850	675;849;850
	187	39;670;827;828	39;670;827;828
	26	413;764;921	413;764;921
K;WLDHPEELSEAFAK	97	172;729;748;777;989	172;729;748;777;989
	75	119;219;422;510	119;219;422;510
	37	300;396;918;920	300;396;918;920
	2	529;807;978	529;807;978
	138	612;767	612;767
	6	312;870;977	312;870;977
	107	17;259;639	17;259;639

	31	175;740	175;740
;LSVRPIR	11	287;689;695;697;728;843	287;689;695;697;728;843
	44	87;541;545	87;541;545
	139	417;564	417;564
	47	499;739;785	499;739;785
	58	669	669
	137	841;842;889	841;842;889
	100	431	431
	119	38;230;484	38;230;484
	103	274;875;961	274;875;961
	131	109;168;822	109;168;822
	88	53;455	53;455
	208	319;571;660;804	319;571;660;804
	40	597;742;788	597;742;788
	16	61;935;1011	61;935;1011
	53	63;212;265;491;493	63;212;265;491;493
	28	730;731;982	730;731;982
	76	894;926	894;926
	33	718;806;905	718;806;905
	133	255;393;818	255;393;818
	196	80;113;187;890	80;113;187;890
	79	238;1015	238;1015
	17	330;468	330;468
	48	207;284;760	207;284;760
	78	613;855	613;855
	180	516;614	516;614
	177	126;588	126;588
	216	325;542;708;717	325;542;708;717
	45	295;954	295;954
	126	297;1014	297;1014
	215	335;407;737	335;407;737
	189	520;521	520;521
	121	515;626	515;626
	190	263	263
	188	154	154
	170	247;716	247;716
	50	834;966	834;966
	118	149;463;589	149;463;589
	171	467	467
	39	535;766	535;766
	104	606	606
	38	29;281	29;281
	62	189;610	189;610
	49	526	526
	22	216;554	216;554
	128	268;434	268;434
	46	54;685	54;685

186	171;202	171;202
161	30;552	30;552
91	191;912	191;912
4	105;963	105;963
8	305;877	305;877
105	479;567	479;567
174	120	120
176	832	832
41	904;981	904;981
14	20;985	20;985
213	537	537
34	137	137
81	394	394
184	190	190
175	134	134
64	395	395
55	136	136
125	866	866
162	159	159
191	825	825
194	49;207	49;207
149	99;990	99;990
24	659	659
10	611	611
123	210;694	210;694
203	188;721	188;721
92	958	958
168	412;577	412;577
192	569;720	569;720
95	420	420
179	503	503
111	929	929
32	36	36
136	288;583	288;583
207	321	321
157	549	549
212	560	560
218	301;356	301;356
3	811	811
114	528	528
83	386	386
93	852;908	852;908
220	282	282
115	540	540
206	424;765	424;765
178	487	487
65	755;769	755;769

67	623;947	623;947
140	874	874
166	799	799
214	587	587
159	272	272
7	471;474	471;474
160	442	442
15	237	237
169	389	389
122	182	182
153	302	302
66	652	652
30	523	523
158	930	930
94	224;792	224;792
129	494	494
165	949	949
199	11	11
202	893	893
110	505	505
96	45;636	45;636
124	150	150
219	846	846
226	579	579
200	140	140
197	490	490
82	725	725
147	658	658
1	488	488
164	285	285
156	706	706
143	534	534
155	616	616
198	555	555
204	518	518
130	886	886
113	511	511

Evidence IDs	MS/MS IDs	Best MS/MS
364;365;366;367;368;369;37	401;402;403;404;405;406;40	410;449;540;825;1207;1465;
38;39;40;41;42;43;44;45;46;	68;69;70;71;72;73;74;75;76;	83;102;1237;1907;2006;2388
211;212;213;214;215;216;21	249;250;251;252;253;254;25	253;338;355;1226;1301;1610
321;322;323;324;325;326;32	371;372;373;374;375;376;37	376;931;1052;1056;1945;299
781;782;783;784;785;786;78	951;952;953;954;955;956;95	952;1009;1439;1774;1782;27
11;12;13;14;15;16;17;18;19;	22;23;24;25;26;27;28;29;30;	26;473;1962;2107;3346;3850
543;544;545;546;547;548;54	644;645;646;647;648;649;65	656;1214;1520;1827;1993;22
1005;1006;1007;1008;1066;1	1221;1222;1223;1224;1293;1	1222;1294;1568;2236;2348;2
127;128;129;130;131;132;13	161;162;163;164;165;166;16	173;194;1539;1659;1660;178
520;521;522;523;524;525;52	627;628;629;630;631;632;94	628;946;1882;2433;2728;406
272;273;274;275;637;638;63	317;318;319;320;321;322;32	321;788;2140;2324;2786;383
262;263;264;265;266;267;13	309;310;311;312;313;314;16	314;1619;1809;2102;2180;22
348;349;350;351;352;353;35	393;394;395;396;397;398;20	395;2023;2285;2360;2363;25
203;204;205;206;302;303;30	235;236;237;238;239;240;24	243;360;1043;1130;1670;167
169;170;171;172;240;241;24	208;281;282;283;681;682;68	208;282;283;701;713;1848;2
226;227;228;229;230;231;89	262;263;264;265;266;267;26	275;1098;1114;1122;2024;38
140;141;142;143;144;145;14	178;179;180;181;182;183;18	185;510;1586;2670;3275;372
1193;1194;1195;1196;1197;1	1466;1467;1468;1469;1470;1	1467;2370;2371;2457;4634;5
882;883;884;885;886;887;88	1082;1083;1084;1085;1086;1	1090;1211;1249;1784;2117;2
2337;2338;2339;2340;2341;2	2801;2802;2803;2804;2805;2	2833;3613;5308;5319;6516
7;8;9;10;99;100;101;102;103	14;15;16;17;18;19;20;21;137	21;142;209;1059;1992;2497;
469;470;471;472;473;474;47	566;567;568;569;570;571;57	588;606;1343;1394;1494;170
60;61;62;63;626;627;628;62	104;105;768;769;770;771;77	104;775;886;1024;1458;1501
0;1;2;3;4;5;6;193;194;195;4	0;1;2;3;4;5;6;7;8;9;10;11;12;	6;229;559;3677;4880;4882;5
870;871;872;873;874;875;87	1060;1061;1062;1063;1064;1	1078;1100;1225;1484;1607;2
503;504;505;506;507;508;50	610;611;612;613;614;615;61	623;1137;1952;2306;2662;26
1316;1630;1631;1632;1633;1	1611;1981;1982;1983;1984;2	1611;1983;2078;2246;2447;2
1243;1274;1909;1910;1911;1	1536;1576;2291;2292;2293;2	1536;1576;2298;2300;3787;3
755;756;940;941;942;943;94	936;937;1147;1148;1220;198	936;1147;1148;1220;1988;24
1709;1710;1711;1712;1713;1	2047;2048;2049;2050;2051;2	2056;2178;5086;5096;5101;5
107;108;109;110;111;112;11	143;144;145;146;147;148;14	147;625;1157;1160;1593;232
3172;3173;3174;3200;3703;3	3684;3685;3686;3687;3688;3	3684;3722;4357;4511;5357;5
644;645;646;647;648;649;65	792;793;794;795;796;797;79	794;858;859;2070;4529;5995
961;962;1726;1926;2213;344	1175;2069;2308;2632;3994;3	1175;2069;2308;2632;3995;4
555;556;557;558;559;560;56	664;665;666;667;668;669;67	677;1563;1967;2381;3545;45
749;4135;4136;4137;4138;41	930;4863;4864;4865;4866;48	930;4869;5110
531;532;533;534;535;536;53	634;635;636;637;638;639;64	639;3083;3387;4581;5707;63
849;850;851;1671;1672;1673	1039;2013;2014;2015;2016;2	1039;2015;4223;4427;4600;4
624;625;678;679;1565;1566;	765;766;767;838;839;840;84	765;838;1929;3023;5542;617
268;269;270;271;1800;1801;	315;316;2144;2145;2146;214	315;2145;2235;2310;3071;46
329;330;331;332;333;334;33	378;379;380;381;382;383;38	382;911;1276;1533;1865;216
244;245;246;247;248;249;25	284;285;286;287;288;289;29	284;293;307;2082;2098;4820
517;518;519;697;698;699;70	626;862;863;864;865;866;86	626;863;882;2469;2530;2783
588;589;590;591;592;1475;1	717;718;719;720;721;722;72	724;1824;1951;2068;2148;22
901;902;903;904;905;1225;1	1103;1104;1105;1106;1107;1	1105;1503;1504;2113;2274;3

155;156;157;158;159;160;16	195;196;197;198;199;200;20	205;1153;1958;2156;4814
2225;2226;3205;3206;3599;3	2652;3727;4229;4230;4231;4	2652;3727;4229;4803;5059;5
75;76;77;78;79;80;81;82;83;	111;112;113;114;115;116;11	115;1624;2309;2351;2476;29
593;594;595;596;597;598;59	729;730;731;732;3446;3447;	731;3448;3630;3802;3859;50
1036;1037;1038;1039;1040;1	1252;1253;1254;1255;1256;1	1256;2075;2385;2500;2842;3
1848;1849;1850;1851;1852;1	2213;2214;2215;2216;2217;2	2218;3114;4495;4761;5104;5
1048;1395;1396;1397;1398;1	1272;1727;1728;1729;1730;1	1272;1736;2161;2268;2269;3
92;93;94;95;1996;1997;1998;	126;127;128;129;130;131;13	134;135;2392;5463;5720
455;456;457;458;459;460;46	547;548;549;550;551;552;55	554;2313;4842;4938;5955;59
72;73;74;1182;1183;2280;36	109;110;1460;2710;4266;426	110;1460;2710;4269;4628;50
2382;2383;3259;3260;3900;3	2905;2906;2907;2908;3794;3	2906;3794;4608;4715;5634;6
1614;1615;1616;3138;3139;3	1969;3643;3644;3645;3646;3	1969;3652;3860;4513;4614;4
1317;1318;1473;1474;1647;1	1612;1820;1989;1990;1991;2	1612;1820;1989;2486;2903;3
179;180;181;182;183;184;18	213;214;215;216;217;218;21	222;225;1146;1462;3932
199;200;201;202;600;601;60	234;733;734;735;736;737;73	234;748;1287;3494;5712
309;310;311;312;313;917;18	367;368;1126;2234;3801;493	367;368;1126;2234;3801;493
2115;2116;2117;4860;4861;4	2516;2517;5701;5702;5703;6	2517;5703;6088
207;208;209;210;1863;1864;	244;245;246;247;248;2227;2	247;2233;3435;3883;5083;57
219;220;221;3749;3750;3751	257;258;259;260;4418;4419;	259;4419;4477;4500;4936;53
124;125;126;2007;2008;2009	159;160;2402;2403;2404;240	159;2404;3035;3434;4943
405;406;407;408;1060;1061;	456;457;458;459;460;461;46	459;1291;2657;2666;3798;43
86;87;88;89;568;569;570;70	123;124;678;679;680;878;95	124;680;878;950;1028;2143;
619;620;621;622;623;757;75	758;759;760;761;762;763;76	760;762;938;940;1852;4003;
2921;2922;2923;2924;3481;3	3351;3352;3353;3354;4031;4	3354;4032;5006;5598;6084
1625;1626;3407;3408;3409;3	1975;1976;3947;3948;3949;3	1975;3949;3959
2137;2138;2139;2903;3321;3	2538;2539;2540;2541;2542;2	2540;3302;3868;4850;4856;5
320;2685;2686;3572;3573;47	370;3152;3153;4193;5630;57	370;3152;4193;5630;5737;57
1948;1949;1950;2074;2892;2	2328;2329;2330;2331;2477;3	2330;2477;3296
1516;1517;1518;1519;1520;1	1858;1859;1860;1861;1862;1	1858;2741;3680;4729;6050
1312;1313;1314;1761;1762;1	1608;1609;2108;2109;2110;2	1609;2110;2264;2290;4512
96;97;98;2092;2093;2094;20	136;2487;5035;5036;5037;50	136;2487;5037;5267
1018;1019;1482;1483;1484;1	1233;1234;1826;5803;5806	1233;1826;5803;5806
719;720;721;722;723;724;72	888;889;890;891;892;893;89	899;4226;5207
3941;3942;3943;3944;3945;3	4642;4643;5643;5644;5645;5	4642;5644;6117
852;853;4420	1040;1041;5172;5173	1040;5173
1077;1078;1079;1184;3435;4	1312;1313;1314;1461;3986;4	1312;1461;3986;4744;5073
695;696;3436;3437;3438	860;861;3987	860;3987
3809;3810;3811;3812;4639;4	4493;4494;5430;5431;5432;5	4494;5432;5434
236;237;238;239;3782;3783;	280;4456;4457;4458;4459;44	280;4456;5270;5272
2165;2166;2167;2168;2169;2	2578;2579;2580;2581;2582;2	2585;4955;5901
1027;1028;1029;1030;4068;4	1242;4775;4776;4777;4778;4	1242;4782;4892;5033;6374
772;773;1278;2227;2228;305	948;1578;2653;2654;2655;26	948;1578;2653;3543
1686;1687;1688;1689;2088;2	2035;2036;2485;5878;5879;5	2035;2485;5881;5896
3156;3157;4402;4403;4404;5	3673;3674;3675;3676;5148;5	3673;5149;6281
3460;3461;3462;3463;3464;3	4008;4009;4010;4011;4012;4	4011;4958
1736;1737;1738;4801;4802;5	2079;2080;5635;5636;6277;6	2079;5636;6277
122;123;1513;1514;1515;359	156;157;158;1856;1857;4225	156;1857;4225

1045;1046;1047;4133;4134	1265;1266;1267;1268;1269;1	1268;4861
1634;1635;1636;1637;3875;3	1985;4583;4584;4621;4622;4	1985;4584;4622;4625;4773;5
584;585;586;587;3184;3185;	715;716;3697;3698;3699;370	715;3705;3714
2214;2215;2216;2217;3264;3	2633;2634;2635;2636;3800	2635;3800
2965;2966;2967;2968;2969;2	3426;3427;3428;3429;3430;3	3426;4858;5062
3778;3779;3780;3781	4455	4455
4590;4591;4592;4593;4594;4	5365;5366;5367;5368;5369;5	5365;5374;5724
2271;2272;2273;2274;2275;2	2699;2700;2701;2702;2703;2	2709
232;233;234;235;1319;2901;	277;278;279;1613;1614;3298	279;1614;3299
1597;1598;4818;4819;4820;4	1957;5649;5650;5651;5652;5	1957;5653;6168
730;731;1015;1016;1017;449	906;1228;1229;1230;1231;12	906;1229;5257
305;306;307;2424;2425;2426	361;362;363;364;365;2962;	363;2962
1772;1773;1774;3296;3297;3	2116;3838;3839;4364;5129;5	2116;3839;4364;5129
3402;3403;3404;3405;3406;4	3941;3942;3943;3944;3945;3	3942;4876;5070
347;5139;5140;5141;5545;55	392;6008;6009;6010;6523	392;6008;6523
358;1249;1250;1251;1252;15	399;1543;1544;1545;1546;15	399;1544;1909;3369;3380
4073;4074;4075;4076;4077;4	4784;4785;4786;4787;4788;4	4784;4794;6322
4915;4916;4917;4918;4919;4	5742;5743;5744;5745;5746;5	5746;5947
4030;4031;4399;4400;4401;4	4742;4743;5147;5805	4742;5147;5805
1489;1490;1491;1492;1493;2	1828;2479;2480;2481;5217;5	1828;2481;5219
529;530;753;754;1122;1123;	633;934;935;1398;5725	633;934;1398;5725
1367;1368;5570	1663;1664;1665;6553	1664;6553
1813;1814;1815;1816;2535;2	2157;2158;2159;2160;3092	2158;3092
1239;1240;1627;1628;4222;4	1532;1977;1978;1979;4946	1532;1978;4946
3469;3470;3471;3472;3473;4	4018;4019;4020;4021;4022;4	4022;5489
3084;3085;3086;3474;3475	3576;3577;3578;4024;4025	3576;4025
844;845;846;847;848;3367;3	1032;1033;1034;1035;1036;1	1037;3909
1795;1796;3187;3188;3189;3	2142;3706;4680;4741	2142;3706;4680;4741
1667;1668;1669;1670;5244;5	2007;2008;2009;2010;2011;2	2008;6119
1674;1675;1676;1677;1678;5	2022;6550;6551;6552	2022;6551
1834;1835;1836;1837;1838;2	2194;2532;2533;2534;2535;2	2194;2536;4852
3119;3120;3121;3122;3123	3626;3627;3628;3629	3626;3629
3081;3082;3083;3532	3574;3575;4112;4113;4114	3575;4114
1537;1538;1539;1540;1541;1	1889;1890;1891;1892;1893;1	1889
945;946;947	1149;1150;1151;1152	1150
1442;1443;1444;4025;4026;4	1785;4734;4735;4736;4737;4	1785;4736
4554;4555;4556;5319;5320;5	5326;5327;5328;6214	5326;6214
932;933;934;935;2511;2512;	1142;3060;3061;3062;3063;3	1142;3068;3910
2533;2534	3088;3089;3090;3091	3090
3168;3169;3170;3171;4238	3683;4957	3683;4957
3446	3992;3993	3993
196;1617;1618;1619;1620;16	232;1970;1971;1972	232;1972
1127;1128;1129;3458	1400;4006	1400;4006
3147;3148;3149;3150	3659;3660;3661;3662;3663	3662
1273;3219;3220;3221	1575;3745;3746	1575;3746
1572;1573;1574;2288;2289	1936;2734;2735	1936;2734
308;3855	366;4552;4553	366;4553

1025;1026;1220;1221	1241;1498;1499;1500	1241;1499
197;198;3207;3208;3209	233;3728;3729	233;3729
1132;1133;5006;5007	1402;1403;5824;5825	1402;5824
707;708;709;710;5300;5301;5	879;6183;6184;6185	879;6184
1704;1705;1706;1707;1708;4	2045;2046;5663;5664;5665;5	2046;5667
2687;2688;2689;2690;2691;2	3154;3155;3156;3157;3158;3	3224;3806
774;775;776	949	949
4548;4549;4550;4551	5323	5323
4984;5403	5804;6321	5804;6321
136;137;138;139;5419	177;6332	177;6332
3175;3176	3689;3690	3690
890;891;892;893	1095;1096;1097	1095
2082;2083;2084	2482;2483	2482
1130;1131	1401	1401
881	1081	1081
2085;2086;2087	2484	2484
889	1094	1094
4771;4772	5602;5603	5603
963;964;965;966	1176;1177	1176
4510;4511;4512	5266	5266
276;277;278;279;280;1239;1	324;325;326;327;328;329;33	331;1532
680;5442	842;6375	842;6375
3716;3717	4361;4362;4363	4362
3459	4007	4007
1244;3910;3911;3912;3913;3	1537;4615;4616;4617;4618;4	1537;4619
1126;4037;4038	1399;4746	1399;4746
5270;5271;5272;5273;5274;5	6152;6153;6154;6155	6154
2164;3319;3320	2577;3865	2577;3865
3282;3283;3284;3285;4033;4	3828;3829;4745	3829;4745
2224	2651	2651
2980;2981;2982;2983;2984;2	3436;3437;3438;3439;3440;3	3442
5096;5097	5952;5953	5952
222;223;224;225	261	261
1638;1639;1640;3338	1986;3879	1986;3879
1777;1778;1779	2118	2118
3201;3202	3723	3723
3257;3258	3792;3793	3792
1690;1925	2037;2307	2037;2307
4439;4440	5201	5201
3155	3672	3672
2064	2470	2470
4663;4664;4665;4666;4667;4	5459;5460;5813	5459;5813
1623;1624	1973;1974	1973
3183	3696	3696
2231;4235;4236;4237	2658;4956	2658;4956
2919	3349	3349
4200;4257	4934;4977	4934;4977

3513;5205;5206;5207	4086;6083	4086;6083
4814;4815;4816;4817	5648	5648
4368;4369;4370	5103	5103
3359;3360;3361;3362;3363;3	3905;3906;3907;3908	3908
1592;1593;1594	1954;1955	1955
2541;2548	3095;3099	3095;3099
2335;2336	2800	2800
1366	1662	1662
2071;2072	2475	2475
1076	1311	1311
1691;1692;1693;1694	2038	2038
3657	4301;4302	4301
3125;3126;3127	3631;3632	3631
5098;5099;5100;5101	5954	5954
1307;4344	1603;5078	1603;5078
2940	3381	3381
5212	6086;6087	6086
90;91	125	125
4909;4910;4911;4912;4913;4	5740;5741	5741
2993;2994;2995;2996;2997;2	3450;3451;3452;3453;3454;3	3454
261;3574;3575;3576;3577	308;4194	308;4194
936	1143	1143
4617	5390	5390
3325	3869	3869
898;899;900	1101;1102	1101
2925	3355	3355
4051	4762	4762
3715	4360	4360
2920	3350	3350
1629	1980	1980
3971;3972;3973	4673	4673
3167	3682	3682
3479;3480	4030	4030
3222	3747	3747
3111;3112;3113;3114;3115	3624	3624
4875;4876	5714	5714
3059;3060	3544	3544

Oxidation (M) site IDs	Oxidation (M) site positions
1554;1596;1923;2041;2044;2199;2501;2514;3396;3425;3529;4221;4260;5113;6190;6208;6300;6318;6	
;2490;2622;3378;3983;4034;4080;4452;4927;6391;6481;6505;6514	
;1643;1658;1960;2127;2133;2304;2356;2524;3105;3277;3369;3380;4299;4411;4429;5058;5497;5502,	
0;3003;3109;4151;4550;4590;4656;4883;4891;4948;4953;4995;5004;5022;5029;5381;5387;6256	
81;2878;4567;6326;6331;6494;6497;6528	
;4286;4624;5734;5771;5832;5944;6245;6587	
12;2375;2377;2946;3046;3059;3080;3751;4103;4188;4190;4192;4275;4282;4393;4483;5253;5642;59	
659;4029;4054;4695;5190;5948;5949;6060;6076;6082;6402	
8;1956;2318;2372;2799;3669;3760;3989;4711;5478;6437	
0;4252;4748;4770;4772;5353;6435;6447;6460	
3;4122;4675;6485;6560	
61;3098;3710;3741;4520;5074;5229;5810	
94;2795;4702;5397;5428;5697;5814;5820	
7;1800;2914;4322	
556;3010;5970;6043	
25;4091;4384;5792	
4;3848;3961;3966;4492;5066;5216;5594;5797;6058	
211;5214;5333;6144	
342;3638;3878;4240;4907;5056;6157	
2910;3917;3928;4336;5905;6414	
7;1725;1838;2576;3565;3895	
;1577;4115;4166;4975	
513;5821;6241	
417;3096;3880;5155	
90;4666;5204;5275;5910	
925;4177;5137;5324;5535;6151	
788;3877;5611;5884	
73;3721;3872;3929;3930;4569;5084;5877	
689;5692	
2;3491;4470;5258;6360;6380	
452;5871;5876	
636;4637;5031;5658;5766;6465	
04;4897;4980;6215;6216	
78	
877;6521	
2	
46;5359;5601	
6;2645;2649;4697;5779	
;2959;3541;5225;5693	
22;3864;4270;4947	
094;4900;5864	

061;6103;6110
21;5531
48;5263;6158
694;4005;5202
715
717;3914

57
34;5638;6372
229
757;6549
691;4085;4933;4940;5123

2;5802;6224

56
62

00;5694;6221
3093;3285
5102

069
74;6233

[illegible]416;6420

;6013;6030;6056;6065;6271;6357

12
