# Supplementary material for: Elucidation of the Antimycobacterial Activity of D-Form Human Lactoferricin 1–11 (D-Form hLF 1–11) Against Mycobacterium smegmatis Through Proteomics and Imaging Analysis
Source: Antibiotics (Basel). 2026 Jun 15;15(6):607. doi: 10.3390/antibiotics15060607 (PMC13296235; doi:10.3390/antibiotics15060607)
Supplement: Supplementary file 1 [file antibiotics-15-00607-s001.zip › supplementary files-Figure S1-Figure S2 & Table S2.pdf]

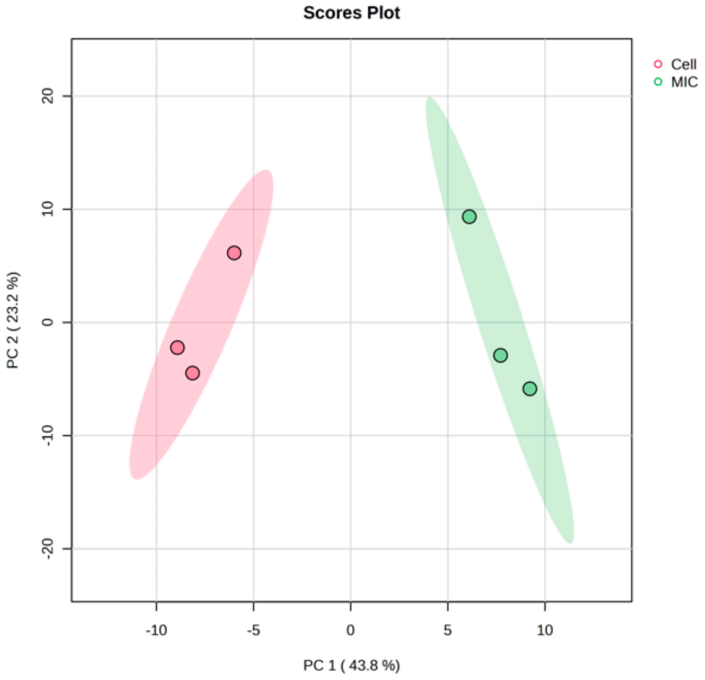
Scores Plot
Cell
MIC
PC 2 (23.2 %)
PC 1 (43.8 %)
20
10
0
-10
-20
-10
-5
0
5
10

(A)

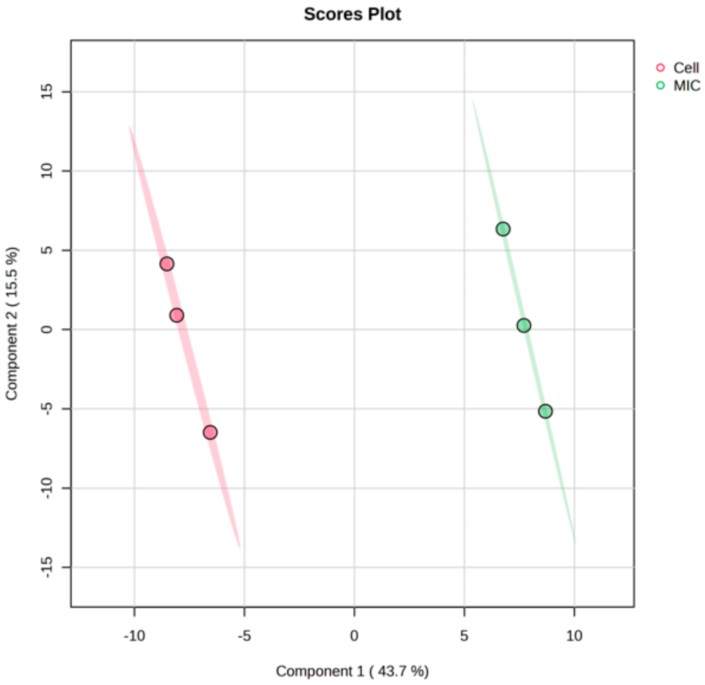
Scores Plot
Cell
MIC
Component 2 (15.5 %)
Component 1 (43.7 %)
15
10
5
0
-5
-10
-15
-10
-5
0
5
10

(B)

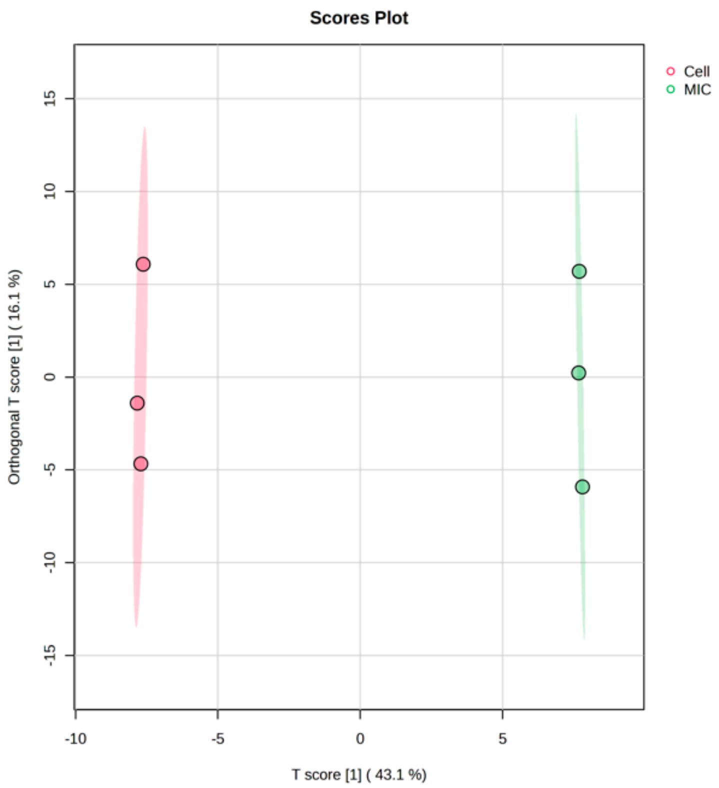

(C)

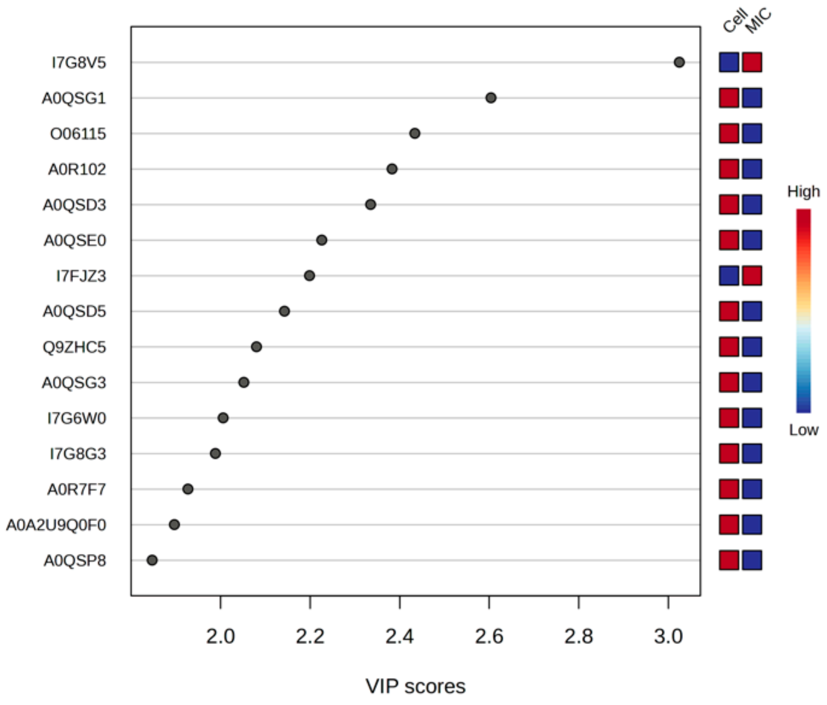

(D)

Figure S1. PCA and PLS-DA Analyses

Principal component analysis (PCA) score plot (A) revealed a clear separation between the cell control group and the MIC (peptide-treated) group along the x-axis (PC1), while intra-group variation was observed along the y-axis (PC2). PC1 and PC2 accounted for 43.8% and 23.2% of the total variance, respectively, at a 95% confidence interval. Partial least squares–discriminant analysis (PLS-DA) score plot (B) demonstrated

the covariance between component 1 (43.7%) on the x-axis and component 2 (15.5%) on the y-axis. Orthogonal partial least squares–discriminant analysis (OPLS-DA) score plot (C) further illustrated distinct sample discrimination. Fifteen proteins with significant variable importance in projection (VIP) scores identified by OPLS-DA are shown in panel (D). Among these, the transcriptional regulatory protein (I7G8V5) and dienelactone hydrolase (I7FJZ3) were highly expressed in the peptide-treated group. In contrast, the remaining proteins—including ribosomal proteins of the large subunit (rplE (A0QSG1), rplV (O06115) rplW (A0QSD3), rplM (A0QSP8)), ribosomal proteins of the small subunit (rpsT (A0R102), rpsQ (A0QSE0), rpsS (A0QSD5), rpsH (A0QSG3), rpsR2 (A0R7F7)), DNA-binding protein Hlp (Q9ZHC5), ATP-binding protein (I7G6W0), histidinal dehydrogenase (I7G8G3), and hydroxyaqualene dehydroxylase (A0A2U9Q0F0) were predominantly expressed in the cell control group. A color gradient ranging from red to blue represents high to low relative expression levels, respectively.

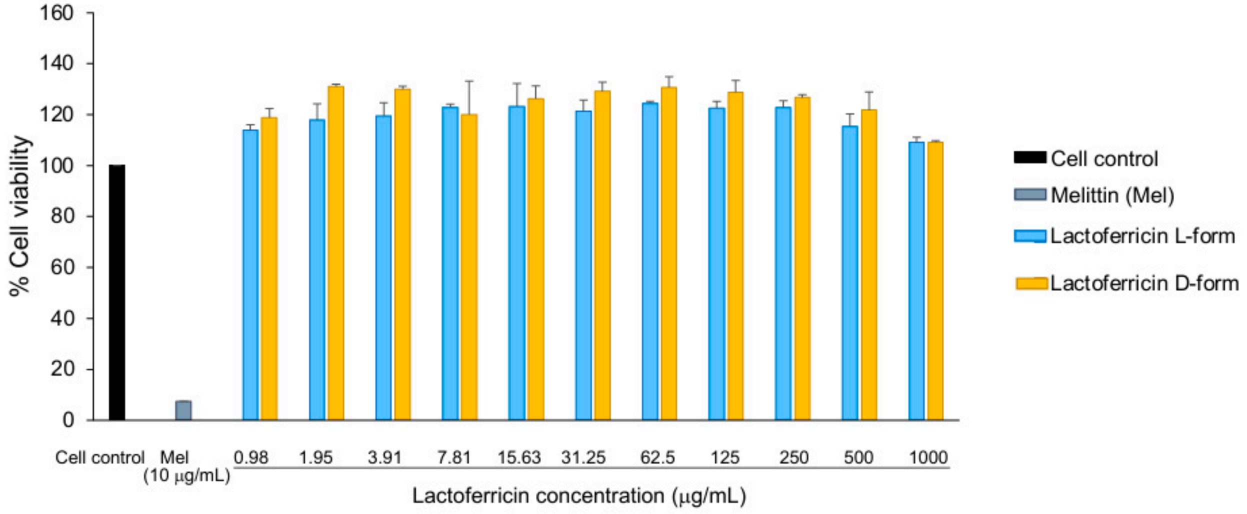

Figure S2. Cytotoxicity assessment of D-form and L-form hLF 1–11 against human alveolar epithelial cell lines (A549 cell lines)

The human lung carcinoma cell line A549 (1×10^4 cells/well) was seeded into a 96-well tissue culture plate and incubated at 37 °C in a humidified atmosphere containing 5% CO_2 for 24 h. The cells were subsequently treated with various concentrations of L-form, D-form hLF 1-11, or melittin (10 µg/mL) as a positive control. After 24 h of treatment, cell viability was assessed by adding 20 µL of 0.01% resazurin solution followed by incubation for 24 h. The reduction of resazurin (blue) to resorufin (pink) was then measured spectrophotometrically at 560 nm. Experiments were performed in independently repeated three times.

Table S2. Summary of 38 significantly different proteins (p value < 0.05) in bacterial control group and D-form hLF 1-11 treated group

Selected protein ID	Protein name	Gene name	Number of proteins	Razor + unique peptides	Sequence coverage (%)	Score	Fold change	Raw p-value	Avg. LFQ cell control	Avg. LFQ peptide treated
A0A2U9PZ50	Methanol:N,N-dimethyl-4-nitrosoaniline oxidoreductase (EC 1.1.99.37) (Methanol dehydrogenase (nicotinoprotein)) (Methanol:NDMA oxidoreductase)	D806_061380	4	26	62.9	182.9	2.206	0.000057282	39,253.00	17,676.33
A0A2U9PVX0	L-glutamate gamma-semialdehyde dehydrogenase (EC 1.2.1.88) (L-glutamate gamma-semialdehyde dehydrogenase)	D806_050100	4	18	40.6	155.98	0.6553	0.0065116	19,850.00	30,291.67
A0QQU5	Chaperonin GroEL 2 (EC 5.6.1.7) (60 kDa chaperonin 2) (Chaperonin-60 2) (Cpn60 2)	groEL2 groL2 MSMEG_0880 MSMEI_0859	10	30	50.8	149.83	1.279	0.028636	20,576.67	16,088.00
A0A2U9PQI5	Glyceraldehyde-3-phosphate dehydrogenase (EC 1.2.1.-)	D806_030480	3	14	55.0	95.425	0.76971	0.0048289	30,008.33	38,986.67
I7G5V6	Probable aldehyde dehydrogenase (EC 1.2.1.3)	MSMEI_1506	7	14	37.1	74.6	1.9369	0.011689	22,254.67	11,489.73
Q9LBQ3	Ribulokinase (EC 2.7.1.16)	araB	5	10	26.1	72.989	1.9755	0.01984	8,335.77	4,219.47
A0A2U9PKX4	Elongation factor Tu (EF-Tu) (EC 3.6.5.3)	tuf D806_014190	3	13	44.7	72.592	3.0968	0.0095671	19,491.33	6,294.10
I7FRX4	Acetyl-CoA acetyltransferase (EC 2.3.1.-)	fadA3 MSMEI_5134	4	9	27.7	61.817	0.51065	0.0042598	7,784.20	15,243.67
Q9ZHC5	DNA-binding protein HupB (HupB) (EC 1.16.3.1) (Cold induced protein) (CipMa) (DNA-binding protein HU) (Histone-like protein) (Hlp) (Mycobacterial DNA-binding protein 1) (MDP1)	hup cipMa hlp MSMEG_2389 MSMEI_2329	3	9	38.9	55.672	82.267	0.013523	36,279.67	399.89
I7GDR8	Fumarate hydratase class II (Fumarase C) (EC 4.2.1.2) (Aerobic fumarase) (Iron-independent fumarase)	fumC MSMEI_5102	4	12	34.4	52.256	0.57859	0.012421	6,713.07	11,602.43

A0QT96	aldehyde dehydrogenase (NAD(+)) (EC 1.2.1.3)	MSMEG_1762	3	9	25.4	47.21	0.74216	0.037628	7,752.53	10,445.87
A0QSZ3	Isocitrate dehydrogenase [NADP] (EC 1.1.1.42) (Oxalosuccinate decarboxylase)	MSMEG_1654	3	10	17.8	46.95	0.46218	0.00081764	7,572.77	16,385.00
A0R200	ATP synthase subunit beta (EC 7.1.2.2) (ATP synthase F1 sector subunit beta) (F-ATPase subunit beta)	atpD MSMEG_4936 MSMEI_4809	2	11	33.1	44.464	1.8004	0.046823	10,596.83	5,885.90
I7G6G9	Biotin-dependent acyl-coenzyme A carboxylase alpha3 subunit (EC 6.3.4.14)	accA3 MSMEI_1762	3	13	31.4	42.699	1.8613	0.01981	8,125.70	4,365.53
A0QWS8	Integration host factor (Mycobacterial integration host factor) (mIHF) (msIHF)	mIHF msihf MSMEG_3050 MSMEI_2975	2	6	52.4	33.485	2.8304	0.0025002	27,361.67	9,667.10
P41193	Small ribosomal subunit protein uS7 (30S ribosomal protein S7)	rpsG	3	6	52.6	32.313	25.884	0.0063601	12,196.10	471.18
A0QSD4	Large ribosomal subunit protein uL2 (50S ribosomal protein L2)	rplB MSMEG_1439 MSMEI_1403	2	7	38.5	32.158	6.8246	0.0032264	8,711.47	1,276.49
A0R758	L-erythrulose 1-kinase (EC 2.7.1.209)	lerK MSMEG_6788 MSMEI_6605	3	8	20.8	30.884	3.0646	0.036277	6,561.50	2,141.03
Q9AGJ6	Phosphoenolpyruvate carboxykinase [GTP] (PEP carboxykinase) (PEPCK) (EC 4.1.1.32) (GTP-dependent phosphoenolpyruvate carboxykinase) (GTP-PEPCK)	pckG pck	3	8	17.7	23.273	0.58314	0.032108	3,947.70	6,769.73
Q939T2	Glutamine synthetase (EC 6.3.1.2)	glnA1	3	8	24.3	22.615	0.46405	0.002099	5,161.50	11,122.73
A0QS62	Large ribosomal subunit protein uL10 (50S ribosomal protein L10)	rplJ MSMEG_1364 MSMEI_1325	2	8	48.0	18.179	1.7032	0.0033768	6,199.77	3,640.13
I7G4E1	Isocitrate lyase (EC 4.1.3.1)	icl MSMEI_0889	3	3	11.2	15.172	2.4352	0.039042	10,803.83	4,436.60
A0QXX7	Catalase-peroxidase 2 (CP 2) (EC 1.11.1.21) (Peroxidase/catalase 2)	katG2 katH MSMEG_3461 MSMEI_3380	3	5	11.8	10.55	0.43333	0.0056987	3,184.80	7,349.53

A0QSD3	Large ribosomal subunit protein uL23 (50S ribosomal protein L23)	rplW MSMEG_1438 MSMEI_1402	2	2	31.0	10.209	46.5	5.4223E-06	5,735.37	0.00
A0QSG1	Large ribosomal subunit protein uL5 (50S ribosomal protein L5)	rplE MSMEG_1467 MSMEI_1431	2	3	19.8	9.8384	90.811	3.5699E-08	11,200.67	0.00
A0QSP8	Large ribosomal subunit protein uL13 (50S ribosomal protein L13)	rplM MSMEG_1556 MSMEI_1519	3	3	21.8	9.7844	20.287	0.026929	6,799.53	252.94
A0QSE0	Small ribosomal subunit protein uS17 (30S ribosomal protein S17)	rpsQ MSMEG_1445 MSMEI_1409	2	3	27.6	7.8356	34.044	8.1495E-07	4,198.93	0.00
O06115	Large ribosomal subunit protein uL22 (50S ribosomal protein L22)	rplV	3	3	22.2	7.7038	58.804	1.0416E-06	7,252.87	0.00
A0QSG3	Small ribosomal subunit protein uS8 (30S ribosomal protein S8)	rpsH MSMEG_1469 MSMEI_1433	2	3	29.5	6.7854	21.411	3.6708E-08	2,640.87	0.00
A0R1Z9	ATP synthase epsilon chain (ATP synthase F1 sector epsilon subunit) (F-ATPase epsilon subunit)	atpC MSMEG_4935 MSMEI_4808	2	3	33.1	6.2134	1.377	0.019011	4,793.67	3,481.13
A0R7F7	Small ribosomal subunit protein bS18B (30S ribosomal protein S18 2)	rpsR2 rpsR1 MSMEG_6895 MSMEI_6711	2	1	14.3	5.2852	21.149	0.024365	7,966.27	294.44
I7FJZ3	Dienelactone hydrolase (EC 3.1.1.45)	MSMEI_2605	3	1	6.4	3.2011	0.029557	0.000028633	0.00	4,172.93
I7G6W0	ATP-binding protein	MSMEI_1892	3	1	15.2	3.0406	19.745	0.000014653	2,435.30	0.00
A0R102	Small ribosomal subunit protein bS20 (30S ribosomal protein S20)	rpsT MSMEG_4571 MSMEI_4459	2	2	34.9	2.5547	58.925	0.000070606	7,267.77	0.00
A0QXD8	Erythritol/L-threitol dehydrogenase (EC 1.1.1.-)	eltD MSMEG_3265 MSMEI_3181	2	1	3.0	2.5038	0.26167	0.0005285	1,105.38	4,224.27
I7G8V5	Transcriptional regulatory protein PdtaR	Transcriptional regulatory protein PdtaR	4	2	11.5	1.9469	0.0031733	0.00003121	0.00	38,867.67
I7G8G3	Histidinol dehydrogenase	MSMEI_5507	3	1	2.0	1.7699	18.548	4.6069E-06	2,287.67	0.00

A0QSD5	Small ribosomal subunit protein uS19 (30S ribosomal protein S19)	rpsS MSMEG_1440 MSMEI_1404	2	1	8.6	1.6052	27.359	5.0706E-07	3,374.50	0.00
